# Economic Cycles and Entry into Parenthood: Is the Association Changing and Does it Affect Macro-Level Trends? Micro-Level Hazard and Simulation Models of Belgian Fertility Trends, 1960–2010

**DOI:** 10.1007/s10680-024-09695-6

**Published:** 2024-03-29

**Authors:** Karel Neels, Leen Marynissen, Jonas Wood

**Affiliations:** Centre for Population, Family & Health, University of Antwerp, Antwerp, Belgium

**Keywords:** First births, Economic context, Recession, Hazard model, Microsimulation

## Abstract

The association between economic cycles—typically measured in terms of GDP growth or swings in unemployment—and macro-level fertility trends has received ample attention in the literature. Compared to studies that consider macro-level fertility, individual-based models can address the association between economic cycles and specific stages of family formation (e.g. entry into parenthood) more precisely while allowing for structural factors that contribute to fertility postponement. Using population-wide longitudinal microdata from the Belgian censuses we combine discrete-time hazard models of entry into parenthood for the period 1960 to 2010 with microsimulation models to assess whether economic cycles in tandem with educational expansion can account for year-to-year variation in the proportion of women entering parenthood and variation in the pace of fertility postponement at the macro-level. Results indicate that educational expansion has been a structural driver of fertility postponement, whereas the procyclical effect of economic cycles accounts for accelerations and decelerations of fertility postponement throughout the period considered. Microsimulation of macro-level fertility trends indicates that individual-based models predict the annual proportion of women entering parenthood and the mean age at first birth with average errors of prediction below 1 per cent and 3 months, respectively, while also showing strong correlations between first differences of observed and simulated time-series. Because the extended observation window encompasses several severe recessions, we test whether the association between economic cycles and entry into parenthood has changed over time and how this affected macro-level trends, discussing several mechanisms that may account for such temporal variation.

**Supplementary Information:**

The online version contains supplementary material available at 10.1007/s10680-024-09695-6.

## Introduction

Postponement of family formation and decline of period fertility levels below the level to replace generations have become structural features of Belgian fertility since the early 1970s, as in most countries across Europe (Neels et al., 2017). Whereas the decline of higher-order births was the most important driver of fertility decline between the mid 1960s and the mid 1970s, parity progression ratios to third- and higher-order births have remained stable at low levels since 1975 (Neels, 2006; Neels & Gadeyne, 2010). In contrast, the advancement of parenthood to increasingly younger ages that persisted throughout most of the 1960s reversed around 1972, and postponement of childbearing has since become the main driver of fertility decline with women’s mean age at first birth increasing from 24.0 years in 1972 to 29.2 years in 2020 (EUROSTAT, 2022).

Several factors have been suggested to drive the trend to later childbearing, such as the introduction and diffusion of efficient contraceptive technology; rising enrolment into tertiary education and educational attainment; increasing labour force participation; the shift to an individualistic family model, in tandem with the changing role and position of children; rising gender equity in education and the labour market; changing partnership patterns and declining real wages (Blossfeld et al., 2005; McDonald, 2013; Mills et al., 2011). Although a sizeable body of literature has documented the associations between these factors and fertility outcomes at the individual level, only a limited number of studies have attempted to quantify the contribution of such factors to macro-level trends in postponement of parenthood (Neels & De Wachter, 2010; Neels et al., 2017; Ni Bhrolchain & Beaujouan, 2012). For Belgium, France and the UK, a limited number of studies have quantified the contribution of increasing education to the aggregate trend of postponement of parenthood. These studies indicate that roughly 50 per cent of the increase in the period MAC1 between 1970 and 2000 is accounted for by the negative association between rising proportions of young adults who are enrolled in education and entry into parenthood, as well as changes in the distribution of social age, reflecting that young adults reach stages associated with the transition to adulthood at increasingly older ages as a result of protracted educational careers (Neels et al., 2017).

Although the mean age at first birth has now increased quasi-monotonically over a period of 40 years, the *pace* of postponement has been subject to variation over time—periods of accelerated postponement alternating with periods of deceleration—which cannot be accounted for in terms of educational expansion unfolding gradually over the period considered. As the timing of life course transitions in early adulthood is strongly linked to employment outcomes, (anticipated) earnings trajectories and household income (Becker, 1960; Hotz et al., 1996), a sizeable literature has considered the association between economic cycles and variation in the tempo of fertility (Sobotka et al., 2011). Although mechanisms leading to countercyclical fertility have been suggested (Butz & Ward, 1979; Vikat, 2004), the bulk of the empirical evidence has found procyclical fertility patterns where family formation is postponed and period fertility levels are deflated under adverse economic and labour market conditions (Bongaarts & Feeney, 1998; Sobotka et al., 2011). The empirical literature has typically considered the association between aggregate-level economic indicators and aggregate-level indicators of (order-specific) fertility (e.g. GDP and period TFR), or has included aggregate-level economic indicators in individual-based models which allow to incorporate a larger number of controls that affect fertility outcomes (Adsera & Menendez, 2011; Neels et al., 2013). Few studies, however, have attempted to quantify the added value of incorporating contextual economic indicators in individual-based microsimulation models (IBMs) to assess whether such models can effectively explain (and predict) macro-level trends, in line with the two-stage perspective on demographic inquiry recently advocated by Billari (2015). As the association between economic context and individual fertility behaviour may change over time, the predictive validity of individual-based models of aggregate fertility trends over subsequent economic cycles requires continuous monitoring. This is particularly relevant as recent studies have suggested that the association between economic context and fertility trends may in fact be weakening in Western and Northern European countries, although the association seems to persist in Southern European as well as Central and Eastern European countries (Comolli et al., 2021; Matysiak et al., 2021).

This paper uses population-wide longitudinal microdata from the Belgian censuses of 2001 and 2011 to study the association between economic cycles and entry into parenthood between 1960 and 2010 among women aged 15 to 50 years. We use discrete-time hazard models to assess whether and to what extent year-to-year variation in the proportion of women having a first child (synthetic parity progression ratio to a first child, SPPR1) and the synthetic mean age at entry into parenthood (SMAC1) is associated with economic cycles, allowing for increasing enrolment in education and the concomitant lengthening of educational careers that contributed structurally to the postponement of parenthood over the period considered (Research Question 1). In addition, we test whether the effect of economic cycles on women’s entry into parenthood has changed over subsequent recession periods, and we assess the added value of including period variation in the effect of economic cycles for the prediction of macro-level fertility trends (Research Question 2).

Our paper contributes to the literature in three ways. First, although a large number of factors affecting the hazard of entering parenthood have been identified using longitudinal microdata, only a limited body of research has used observation plans that allow to test whether and to what extent individual-based models (IBMs) are effectively capable of generating the observed macro-level fertility trends. This paper adds to the literature by including contextual indicators of economic context in micro-level hazard and simulation models of entry into parenthood. Simulated macro-level time-series of the synthetic parity progression ratio to a first birth (SPPR1) and the synthetic mean age at entry into parenthood (SMAC1) are evaluated against the observed time-series of these indicators to assess model performance. Second, using population-wide longitudinal microdata from the Belgian censuses of 2001 and 2011 we can assess the association between individual-level birth hazards and economic context over an extended period of 50 years and test whether the association has changed over subsequent recessions between 1960 and 2010. Only a limited number of papers have been able to study this association over subsequent recessions using consistent data that cover a sufficiently long time period (Ahn & Mira, 2001). Third and finally, by using hazard models as input for dynamic microsimulation models we add to the literature on fertility forecasting as hazard models allow to incorporate a larger set of determinants than is typically feasible in the decomposition of aggregate-level fertility rates such as the period TFR. The Belgian context seems appropriate to address these questions as both economic cycles and fertility trends in Belgium show strong communalities with economic and demographic trends in other European countries over the period considered.

## Economic Cycles and Fertility: Mechanisms and Previous Findings

The association between economic cycles and fertility is the outcome of countervailing forces where it may be advantageous for some individuals and households to have a child in economic uncertain times, resulting in a countercyclical fertility response, whereas others will postpone having a child or refrain from childbearing altogether, giving rise to a procyclical response (Sobotka et al., 2011). In addition to such population heterogeneity, the strength of the association and the time lag in the response will vary depending on the properties of the economic and demographic indicators considered. Economic indicators vary in terms of the speed at which they pick up changes in the economic conditions that affect households. Similarly, there is considerable variation between demographic indicators in the extent to which they are capable of accurately measuring changes in the fertility behaviour of the groups most strongly affected by an economic downturn. Finally, the opportunity costs of childbearing under economically adverse conditions and the resulting association between economic recession and fertility have been suggested to be strongly contingent on policy context (Butz & Ward, 1979; Comolli et al., 2021; Sobotka et al., 2011). We consider each of these aspects in turn.

### Procyclical Versus Countercyclical Mechanisms

Several mechanisms have been suggested that link variation in economic conditions to fertility outcomes. On the one hand economic downturns have been suggested to reduce women’s opportunity cost of childbearing resulting in countercyclical fertility (Butz & Ward, 1979; Vikat, 2004). In contrast, the negative effect of economic downturns on household income has been suggested to generate a procyclical association as women and couples will refrain from making long-term irreversible investments when financial and labour market insecurity increases, income levels drop and labour market prospects deteriorate (Becker, 1960; Comolli et al., 2019). Whereas countercyclical fertility responses have been documented (Vikat, 2004), the empirical evidence seems to suggest that mechanisms leading to procyclical responses prevail as most studies have found aggregate-level fertility to decline in response to worsening economic conditions, where the fertility response typically follows at a lag of one to two and a half years (Sobotka et al., 2011).

Economic downturn affects the income and employment position of young adults through various pathways, such as: (i) *increasing enrolment* in education as a result of deteriorating employment prospects for young adults, (ii) *increasing unemployment, deteriorating job conditions and overeducation* that negatively affect the income position of individuals and households, (iii) *reduction of job opportunities and delayed entry into anticipated career tracks*, and (iv) *reductions in public spending that affect labour market, social and family policies* (e.g., austerity measures affecting unemployment benefits, parental leave benefits and public childcare) that further affect the income position of households and the possibility to combine work and family (Sobotka et al., 2011). These adverse economic outcomes have in turn been shown to affect health outcomes (Burgard & Kalousova, 2015), union formation and dissolution (Cohen, 2014; Schneider & Hastings, 2015), home leaving (Bertolini & Goglio, 2019), living arrangements (Lee & Painter, 2013), as well as fertility intentions and outcomes (Guzzo, 2022; Matysiak et al., 2021).

Considering population heterogeneity in the opportunity cost of childbearing under economically adverse conditions, the association between economic recession and fertility is subject to variation by age, sex, ethnicity, socio-economic status and current family size. Economic downturn seems to affect fertility outcomes more strongly in lower birth-orders (Adsera & Menendez, 2011), younger age groups (Neels et al., 2013) and the higher educated (Adsera & Menendez, 2011; Neels et al., 2013; Sobotka et al., 2011). Finally, the literature has focused predominantly on short-term variation of fertility in response to economic conditions. The association between economic shocks and long-term fertility seems weaker, suggesting recuperation of fertility at longer time lags, although empirical evidence on recuperation effects remains scant (see, however, Neels (2010)).

### Measurement Issues

The empirical literature on the association between economic cycles and fertility is elaborate, but also diverse in a number of important respects. A first point of variation concerns the definition of economic recession and the economic indicators considered. A widely accepted definition of economic recession is lacking (Sobotka et al., 2011), but negative growth of the gross domestic product (GDP) over a number of consecutive quarters has frequently been used as an indicator. The short-lived character of recessions—negative GDP growth is typically confined to a limited number of quarters—raises questions on how the precise timing of negative GDP growth relates to trends in other economic and labour-market indicators leading up to and following the actual recession. The effect of recession on fertility will differ depending on whether negative GDP growth is a leading indicator of economic downturn or a lagging indicator of deteriorating economic conditions in the period preceding the recession (Buckles et al., 2021). The discussion extends to other indicators of economic conditions. The association with fertility (and the lag in the response) requires careful contextualisation depending on whether indicators are early signals or lagging indicators of the deteriorating economic and labour market conditions which households face. A variety of aggregate-level economic indicators have been considered, including (change in) gross domestic product, consumer confidence, (change in) consumer price indices and unemployment, but the empirical association between fertility outcomes and unemployment rates seems most consistent and robust empirically, suggesting that unemployment is a more tangible indicator of the impact of economic downturns on men and women of reproductive age (Sobotka et al., 2011). Regarding the time sequence of changes in period fertility and economic cycles, the majority of studies suggest that recession is a leading indicator with respect to fertility behaviour, but some studies have in contrast found fertility to be a leading economic indicator (Buckles et al., 2021; Verdickt, 2020).

Also with respect to demographic indicators the literature on the association between economic context and fertility is diverse. Although fertility indicators differ considerably in their capacity to isolate current changes in fertility behaviours from past trends, crude fertility measures have often been used, presumably reflecting a lack of detailed microdata on fertility histories. A large number of studies consider aggregate-level fertility measures such as the number of conceptions or births (Buckles et al., 2021; Verdickt, 2020), crude birth rates or general fertility rates (Schaller, 2016; Schneider, 2015), or age-specific fertility rates and associated measures such as period total fertility rates or period mean age at childbearing (Comolli, 2017). Research considering numbers of conceptions or births over time, crude birth rates or general fertility rates by definition fail to control for changes in the size and/or age distribution of the (female) population. Age-standardized indicators such as the period TFR and the period MAC remain sensitive to changes in the distribution of fertility by birth-order (Ryder, 1980). As a result, secular trends in parity progression that are unrelated to economic context will induce variation in the association between aggregate-level fertility measures and economic indicators when considering long observation periods. Differentiating fertility by birth order may provide a more accurate indication of the stages in family formation most affected by economic downturns, but the accuracy of indicators to pick up current changes in order-specific fertility will depend on whether indicators are capable of also controlling for the parity distribution of the (female) population. Non-decremental age-order-specific fertility rates that relate births of a given order to the (female) population regardless of parity fail to adequately control for past fertility behaviour, and will also introduce bias in the association between fertility and economic conditions as a result. Life table-based indicators and hazard models that link conceptions or births of a given order to the appropriate parity-specific risk set seem most appropriate to control for past fertility behaviour and effectively link current fertility behaviours to current economic conditions while controlling for other factors that affect tempo and quantum of fertility (Adsera & Menendez, 2011; Blossfeld & Rohwer, 2002; Comolli et al., 2021; Neels et al., 2013).

### Country and Period Contingencies: Variation in Nature of Recessions and Policy Context

The procyclical association between economic cycles and fertility seems to hold quite generally in different time periods across different settings (e.g. Silver (1965), Cherlin et al. (2013), Currie and Schwandt (2014), Schneider (2015) and Alam and Bose (2020) for the US; Adler (1997) and Ozcan et al. (2010) and Kreyenfeld (2010) for Germany; Andersson (2000) and Hoem (2000) for Sweden; Kravdal (2002) for Norway; Ahn and Mira (2001) and Puig-Barrachina et al. (2020) for Spain; Caltabiano et al. (2017) for Italy; Kotzamanis et al. (2017) for Greece; Adsera and Menendez (2011) for Latin America and Adsera (2011), Neels et al. (2013), Goldstein et al. (2013), Bellido and Marcen (2019) and Matysiak et al. (2021) for comparative studies encompassing a larger set of countries). Nevertheless, recent studies have suggested that the association between economic cycles and fertility may be weakening in Western and Northern European countries (Comolli et al., 2021; Matysiak et al., 2021) and the USA (Seltzer, 2019) as an increasing number of individuals end up in precarious labour market positions despite declining unemployment and solid economic growth, or as a result of increasing economic uncertainty from a global rather than local or national perspective. The latter findings suggest that continuous monitoring is needed of the association between unemployment, GDP growth and fertility, as well as the association between unemployment, GDP growth and alternative indicators of labour market precariousness.

## Belgian Context

**Fig. 1:**
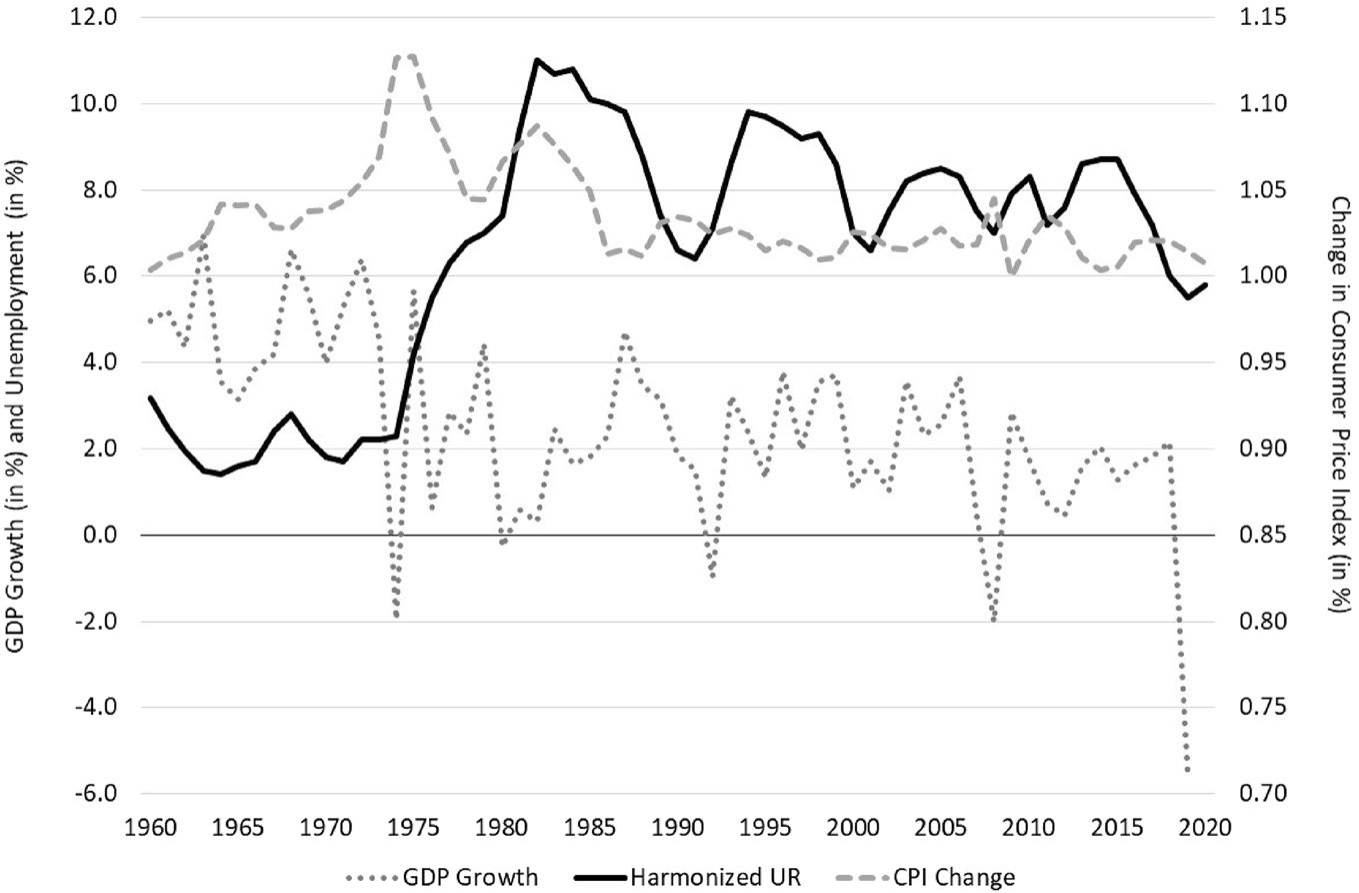
Aggregate-level indicators of economic context, Belgium, 1960–2020 Source: OECD (2022) and National Bank of Belgium (2022)

Following a baby boom around 1964 with a period TFR of 2.75 children per woman, several mechanisms contributed to the decline of Belgian fertility below the replacement level from the early 1970s onwards. A first mechanism is the decline of parity progression to third- and higher-order births after 1965 which largely stabilised by 1975 and seems at least partially related to the diffusion of efficient contraceptives that reduced parity failures among slightly older women (Neels, 2006). Synthetic parity progression ratios to third- and higher-order births declined without exception from 65 to 70 per cent in 1965 to much lower values in 1975—around 35–40 for third births and 30–35 per cent for fourth and higher-order births—and have remained stable around those values since (Neels, 2006; Neels & Gadeyne, 2010). The declining share of third- and higher-order births modified the composition of total fertility by birth-order, contributed to the decline of the period mean age at childbearing (Ryder, 1980), and initially concealed the onset of a second mechanism: the postponement of entry into parenthood to older ages. From the early 1970s onwards, the mean age at entry into parenthood started increasing rapidly as a result of increasing enrolment in education and increasing numbers of young adults attaining tertiary education (Fig. A.2 in annex), in tandem with deteriorating economic conditions affecting young adults’ entry into the labour market (Fig. 1 and Fig. A.3 in annex) (Neels & De Wachter, 2010; Neels et al., 2017). With first and second births now representing the bulk of fertility, the onset of fertility postponement induced considerable deflation in period fertility levels after 1975 (Bongaarts & Feeney, 1998), with the period TFR reaching a trough in 1985 with a value of only 1.51 children per woman. The period mean age at childbearing continued to increase after 1985, but the pace of postponement has been subject to variation over time, which translated into varying levels of deflation and TFR values fluctuating between 1.50 and 1.65 in the period between the mid 1980s until the early 2000s. Although the period TFR started recovering in the early 2000s, reaching a value of 1.85 children per woman in 2008, the Great Recession ushered in a new era of fertility decline with the period TFR again reaching a low value of 1.55 in 2020.

Throughout the 1960s Belgium experienced sustained economic growth (an annual GDP growth of 4.85 per cent on average between 1960 and 1969), resulting in a growth of employment (6.85 per cent between 1960 and 1969) that largely outpaced the growth in the labour force (only 5.81 per cent between 1960 and 1969), and resulted in unemployment levels reaching historically low values (between 2 and 4 per cent throughout the 1960s) (Fig. 1). Economic growth slowed down considerably after the early 1970s with recessions (spells of negative GDP growth) occurring in 1975, 1981 and 1993 that were typically accompanied by protracted spells of negative employment growth that lasted several years (1975–1978, 1980–1984 and 1991–1995). The combination of stalls in employment growth with a continued growth in the labour force (particularly among women, Fig. A.4 in annex) resulted in excess labour supply and unemployment levels increasing to 10.8 per cent (1985) and 9.8 per cent (1995) in the general labour force, while soaring up to levels of 24.4 per cent (1984) and 23.2 per cent (1996) in the youth labour force under age 25, and even higher levels among young women (Fig. A.3 in annex). After 2000, Belgium witnessed additional periods of limited GDP growth in 2001–2003, 2009 (the so-called ‘Great Recession’) and 2020 (the Covid pandemic), but these were not accompanied by protracted relapses in employment growth as had been the case in the 1970s, 1980s and 1990s. This resulted in more limited surges in unemployment levels around 8 per cent between both 2003–2006 and 2010–2016. As before, however, younger age groups and women have been more seriously affected with youth unemployment and women’s unemployment rates typically being considerably higher than unemployment levels in the general labour force (Figure A.3 in Annex). Throughout the observation window changes in the consumer price index have been below 5 per cent, barring periods of increased inflation around the mid 1970s and the early 1980s (Fig. 1).

## Data and Methods

### The 2001 and 2011 Belgian Censuses

The analyses combine retrospective longitudinal microdata from the Belgian censuses of 1 October 2001 and 1 January 2011. The 2001 Belgian census was the last conventional census and collected maternity histories for all women aged 14 and older, providing the year of birth of all children up to the 12th birth. As the census provides information on women’s year of birth and the year of birth of their children, women’s exact ages at the birth of a child are unknown and vary between exact ages $\left(a-1\right)$ and $\left(a+1\right)$ in any year $t$ (Neels, 2006; Wunsch & Termote, 1978). As a result, decremental first birth rates calculated retrospectively from the 2001 Census constitute parallelograms with vertical sides which are centred around exact age $a$ (Wunsch & Termote, 1978). Eliminating unit and item non-response, the 2001 Census provides quasi-exact retrospective estimates of the period TFR published in vital registration between 1960 and 2000 (Neels & Gadeyne, 2010). Being fully register-based, the 2011 Census no longer provides self-reported maternity histories and the maternity histories were constructed using data on descent available in the national register (coded ID of both parents). The register-based maternity histories in the 2011 Census provide quasi-exact retrospective estimates of the Period TFR for the period 1985–2010, reflecting the integration of the population registers and the national register from the mid 1980s onwards. The analysis uses 47,354,001 person-years of exposure to the risk of having a first child among childless women aged 15–50 between 1960 and 2010, where exposure between 1960 and 2000 is drawn from the 2001 Census and exposure between 2001 and 2010 is drawn from the 2011 Census.

Previous work on timing of parenthood in Belgium (Neels et al., 2017) has shown that first birth hazards are low during enrolment in education and subsequently follow an inverse U-curve as a function of duration since leaving education that has remained relatively stable over time. In view of estimating the probability of having a first child at a given age, the analyses consider different indicators that provide information on the precise level of education and the length of schooling it implies in addition to the duration since leaving education: (i) a time-varying dummy variable on *enrolment* in education, (ii) a time-varying variable reflecting the highest *level of education* obtained upon leaving education, and (iii) a time-varying variable reflecting *duration since leaving education* in period difference (quadratic specification). Information on education in the 2001 Census is self-reported and provides information on enrolment at the time of the census, the highest level of education obtained by October 1st 2001, as well as the number of years spent in primary education, secondary education and tertiary education which was used to estimate the year in which individuals left education (Deboosere & Willaert, 2004). Some overreporting of educational attainment in the 2001 Census results from reporting educational levels in which individuals were still enrolled at the time of the census. As a result, the highest level of education was adjusted considering the minimal age required to obtain the reported certificate. In contrast, the 2011 census provides information drawn from the registers of the Dutch, French and German-speaking educational systems in Belgium on enrolment at the time of the census and information on the highest level of education obtained by the time of the census. As the 2011 census provides no retrospective time-varying information on enrolment in education, the age at leaving education was randomly assigned considering the highest level of education obtained by the time of the 2011 census and the distribution of age at leaving education by level of education observed among school-leavers in the period 1995–2000 in the 2001 census, which may induce a slight underestimation of the duration of educational trajectories after 2000. In both censuses the highest level of education was coded using the ISCED97-classification (UNESCO, 2006), distinguishing (i) no education and primary education (ISCED97, 0&1), (ii) lower secondary education (ISCED97, 2), (iii) higher (post-)secondary education (ISCED97, 3&4), (iv) short-type tertiary education (ISCED97, 5B), and (v) long-type tertiary education (ISCED97, 5A&6).

### Economic Indicators

Contextual data on annual *unemployment* rates were obtained from the Centre for Social Policy (CSB) for the period 1947–1960 and the National Bank of Belgium (NBB) for the period 1960–2020 (NBB, 2022). The NBB time-series consists of harmonized data derived from the Labour Force Survey (group of 15–74 years), monthly adjusted by using the administrative national unemployment figures, in accordance with the Eurostat methodology. As the CSB time-series uses a slightly different definition of the labour force resulting in higher unemployment rates, the unemployment rates for 1947–1960 were adjusted to match the harmonised unemployment rate of the National Bank from 1960 onwards.

### Modelling First Birth Hazards

To model entry into parenthood, we follow childless women from the age of 15 up to and including the year in which they have their first child (event occurrence), the year in which they celebrate their 50th birthday (censoring) or the end of the observation period on 31 December 2010 (censoring). As a result of the retrospective design no censoring takes place for other reasons. In the age-time plane of the Lexis-chart, we select person-years in the rectangle delineated by ages 15–50 in the period 1960–2010 which gives rise to an orthogonal design allowing us to test age-variation in the impact of economic context on first birth hazards over time, as well as period variation in the age-specific effect of economic context on first birth hazards. In addition, the rectangular selection in the Lexis chart allows to simulate period fertility indicators from the models on an annual basis which are compared with the observed time-series to assess model performance.

Discrete-time hazard models with a complementary log–log link function are used to model the conditional probability ${q(a)}_{i}^{t}$ of having a first child around exact age $a$ in year $t$ for any woman $i$ who has remained childless up to January 1st of year $t$. Exponentiated parameter estimates reflect covariate effects on women’s cumulative hazard of having a first child in year $t$:

1 $$-{\text{ln}}\left[1-{\widehat{q}\left(a\right)}_{i}^{t}\right]={\int }_{t}^{t+1}h\left(a\right)da=\begin{array}{c}\begin{array}{c}{e}^{\widehat{\alpha }}.{e}^{\widehat{F}({A}_{ti})}.{e}^{\widehat{F}\left({E}_{ti}\right)+\widehat{F}\left({G}_{ti}\right)+\widehat{F}\left({L}_{ti}\right)+\widehat{F}\left({A}_{ti}.{L}_{ti}\right)}.\\ {e}^{\widehat{F}\left({{\text{UR}}1}_{ti}\right)+\widehat{F}\left({{\text{UR}}1}_{ti}.{{\text{A}}}_{ti}\right)+\widehat{F}\left({{\text{UR}}1}_{ti}.{{\text{L}}}_{ti}\right)+\widehat{F}\left({{\text{UR}}1}_{ti}.{{\text{A}}}_{ti}{.{\text{L}}}_{ti}\right)}.\end{array}\\ {e}^{\widehat{F}\left({{\text{UR}}10}_{ti}.{{\text{A}}}_{ti}\right)+\widehat{F}\left({{\text{UR}}10}_{ti}.{{\text{A}}}_{ti}.{{\text{L}}}_{ti}\right)}.\\ \begin{array}{c}{e}^{\widehat{F}\left({{\text{UR}}1}_{ti}.{{\text{P}}}_{ti}\right)+\widehat{F}\left({{\text{UR}}1}_{ti}.{{\text{A}}}_{ti}.{{\text{P}}}_{ti}\right)+\widehat{F}\left({{\text{UR}}1}_{ti}.{{\text{A}}}_{ti}.{{\text{L}}}_{ti}{.{\text{P}}}_{ti}\right)}.\\ {e}^{\widehat{F}\left({{\text{UR}}10}_{ti}.{{\text{P}}}_{ti}\right)+\widehat{F}\left({{\text{UR}}10}_{ti}.{{\text{A}}}_{ti}.{{\text{P}}}_{ti}\right)+\widehat{F}\left({{\text{UR}}10}_{ti}.{{\text{A}}}_{ti}.{{\text{L}}}_{ti}{.{\text{P}}}_{ti}\right)}\end{array}\end{array}$$

where ${A}_{ti}$ reflects *age* in years (period difference) centred at the age of 15 (baseline, cubic specification); ${E}_{ti}$ is a time-varying dummy variable indicating *enrolment in education* with a value of 1 up to and including the age at which women obtained their highest certificate and a value of 0 thereafter; ${G}_{ti}$ denotes *duration since graduation or leaving education* in years (period difference) since women obtained their highest certificate (quadratic specification) and ${L}_{ti}$ denotes the highest *level of education* that women obtained. To model recession-induced postponement (at younger ages) and subsequent recuperation of first births (at older ages), the models include the annual *unemployment rate* at lags of 1 year (${{\text{UR}}1}_{ti}$) and 10 years (${{\text{UR}}10}_{ti}$) respectively (Fig. A.7). Finally, ${P}_{ti}$ is a categorical variable that distinguishes three calendar time periods: the 1960–1973 period of economic growth; the 1970s and 1980s economic recessions during 1974–1991; and the 1992–2010 period encompassing the early 1990s recession and economic downturns with more limited repercussions regarding unemployment during the 2000s.

### Models of Entry into Parenthood

Four blocks of models (I–IV) are estimated to assess the impact of educational expansion and variation in economic context on first birth hazards in Belgium throughout the period considered (Table 1). Block I serves as a reference for further tests with *Model 0* only including centred age (${A}_{ti}$) as the baseline hazard function. Block II additionally includes different indicators of educational expansion. Based on previous research, *Model 1* includes time-varying enrolment in education (${E}_{ti}$), duration since graduation or leaving education (${G}_{ti}$) and educational level (${L}_{ti}$) as first birth hazards are typically quite low during spells of enrolment in education (Blossfeld & Huinink, 1991) and follow a quadratic function of duration since leaving education as women move through subsequent transitions in young adulthood (e.g. finding a partner, employment, forming their own household) that often precede entry into parenthood (Neels et al., 2017). First birth hazards are further differentiated by level of education due to its association with factors such as labour force participation and earning power, attitudes, values and preferences, social learning, knowledge of and access to contraception (Neels et al., 2017). As education is likely to affect the timetable of entry into parenthood net of differential length of educational careers, Model 2 additionally includes the interaction between the baseline and educational level (${A}_{ti}.{L}_{ti}$).

**Table 1:** Model specifications for the effects of educational expansion and variation in economic context on first birth hazard of childless women in Belgium between 1960 and 2010

						Model blocks (I-IV) and Model specifications (0–12)												
Covariates						I	II		III							IV		
						0	1	2	3	4	5	6	7	8	9	10	11	12
*Individual-level covariates*																		
${A}_{ti}$						+	+	+	+	+	+	+	+	+	+	+	+	+
${E}_{ti}$							+	+			+	+	+	+	+	+	+	+
${G}_{ti}$							+	+			+	+	+	+	+	+	+	+
${L}_{ti}$							+	+			+	+	+	+	+	+	+	+
${A}_{ti}.{L}_{ti}$								+			+	+	+	+	+	+	+	+
*Economic context*																		
Unemployment rate, lagged 1 year (UR Lag1)																		
${{\text{UR}}1}_{ti}$									+									
${{\text{UR}}1}_{ti}.{{\text{A}}}_{ti}$										+	+			+				
${{\text{UR}}1}_{ti}.{{\text{L}}}_{ti}$												+						
${{\text{UR}}1}_{ti}.{{\text{A}}}_{ti}{.{\text{L}}}_{ti}$													+		+			
Unemployment rate, lagged 10 years (UR Lag10)																		
${{\text{UR}}10}_{ti}.{{\text{A}}}_{ti}$														+				
${{\text{UR}}10}_{ti}.{{\text{A}}}_{ti}.{{\text{L}}}_{ti}$															+			
Period Variation in the effect of economic context																		
${{\text{UR}}1}_{ti}.{{\text{P}}}_{ti}$																+		
${{\text{UR}}10}_{ti}.{{\text{P}}}_{ti}$																+		
${{\text{UR}}1}_{ti}.{{\text{A}}}_{ti}.{{\text{P}}}_{ti}$																	+	
${{\text{UR}}10}_{ti}.{{\text{A}}}_{ti}.{{\text{P}}}_{ti}$																	+	
${{\text{UR}}1}_{ti}.{{\text{A}}}_{ti}.{{\text{L}}}_{ti}{.{\text{P}}}_{ti}$																		+
${{\text{UR}}10}_{ti}.{{\text{A}}}_{ti}.{{\text{L}}}_{ti}{.{\text{P}}}_{ti}$																		+

Source: Longitudinal microdata from the 2001 and 2011 Belgian censuses, calculations by authors

Block III introduces economic context. Model 3 introduces the harmonised unemployment rate with a one year lag as a proxy for short-term effects of economic downturn (${{\text{UR}}1}_{ti}$), implicitly assuming that all age groups are similarly affected by economic swings, whereas Model 4 allows the short-term effect of economic context to vary by 5-year age groups (${{\text{UR}}1}_{ti}.{{\text{A}}}_{ti}$). Model 5 combines the specification of the effect of economic context in Model 4 with the specification of educational expansion in Model 2. Model 6 allows the short-term effect of economic context to vary by level of education (${{\text{UR}}1}_{ti}.{{\text{L}}}_{ti}$), while Model 7 considers the three-way interaction between the short-term effect of economic context, age and level of education (${{\text{UR}}1}_{ti}.{{\text{A}}}_{ti}{.{\text{L}}}_{ti}$). Model 8 additionally introduces the harmonised unemployment rate with a lag of 10 years as a proxy for long-term effects of economic downturn (${{\text{UR}}10}_{ti}$) as previous research has shown that birth hazards of women aged 30 and older correlate positively with unemployment rates 8 to 12 years earlier, suggesting that women compensate for economic circumstances experienced at younger ages (e.g. recuperation of fertility forgone at younger ages) (Neels, 2010; Neels et al., 2013). Model 9 additionally considers the three-way interactions between age, educational level and both short-term (${{\text{UR}}1}_{ti}.{{\text{A}}}_{ti}{.{\text{L}}}_{ti}$) and long-term effects of economic context (${{\text{UR}}10}_{ti}.{{\text{A}}}_{ti}.{{\text{L}}}_{ti}$).

Finally, Block IV addresses the question whether the short-term and long-term effects of economic context vary over time by considering two-way interactions (${{\text{UR}}1}_{ti}.{{\text{P}}}_{ti}$ and ${{\text{UR}}10}_{ti}.{{\text{P}}}_{ti}$ in Model 10), three-way interactions (${{\text{UR}}1}_{ti}.{{\text{A}}}_{ti}.{{\text{P}}}_{ti}$ and ${{\text{UR}}10}_{ti}.{{\text{A}}}_{ti}.{{\text{P}}}_{ti}$ in Model 11), and four-way interactions (${{\text{UR}}1}_{ti}.{{\text{A}}}_{ti}.{{\text{L}}}_{ti}{.{\text{P}}}_{ti}$ and ${{\text{UR}}10}_{ti}.{{\text{A}}}_{ti}.{{\text{L}}}_{ti}{.{\text{P}}}_{ti}$ in Model 12) between period, age, level of education and economic context. Model estimates are included in Table A.1 in annex.

### Model-Based Time-Series of SPPR1 and SMAC1

For a nulliparous woman $i$ included in the risk set of a given hazard model, the event of having a first child centred around age $a$ in year $t$, denoted ${b(a)}_{ti}$, is simulated using a random generator, where the woman’s predicted conditional probability ${\widehat{q}(a)}_{ti}$ of having a first child in that year according to the hazard model considered is evaluated against a value ${r}_{ti}$ drawn from a *standard uniform distribution* $U\left(\mathrm{0,1}\right)$, where ${b(a)}_{ti}=1$ if ${{r}_{ti}\le \widehat{q}(a)}_{ti}$ and ${b(a)}_{ti}=0$ otherwise (Balakrishnan & Nevzorov, 2003). By using the simulated event log for individual women as input for the retrospective calculation of aggregate-level fertility indicators (Neels, 2006; Vergauwen et al., 2015), the different hazard models give rise to simulated time-series of macro-level fertility indicators under the model considered which can be readily compared to the observed trends. In this paper we focus on the synthetic parity progression ratio to a first birth (${\text{SPPR}}1$) and the corresponding synthetic mean age at first birth (${\text{SMAC}}1$) (Feeney & Yu, 1987; Neels, 2006; Ni Bhrolchain, 1987).

2 $${{\text{SPPR}}1}_{t}=1-\prod_{a=15}^{49}\left[1-{\overline{b }(a)}_{t}\right]$$

3 $${{\text{SMAC}}1}_{t}=\sum_{a=15}^{49}\frac{a\left({S}_{a}^{t}-{S}_{a+1}^{t}\right)}{{{\text{SPPR}}1}_{t}}$$

where ${S}_{a}^{t}$ is the period survivor function calculated from the simulated event occurrences. For the counterfactual time-series of both ${{\text{SPPR}}1}_{t}$ and ${{\text{SMAC}}1}_{t}$ we present the mean values of their distributions based on 120 simulations.

### Assessing Model Fit and Correspondence Between Observed and Simulated Time-Series

Different indicators are used to assess the fit of the hazard models, as well as the correspondence between observed trends and the simulated time-series of SPPR1 and SMAC1. Model fit is evaluated by comparing deviance statistics across models using likelihood ratio (LR) tests, and comparing associated AIC and BIC values. Although LR-tests indicate whether a covariate has a significant impact on women’s hazard to enter parenthood, the LR-test does not provide information on whether the change in the distribution of the covariate over time is such that it also accounts for *macro-level trends in SPPR1 and SMAC1* throughout the period considered. To assess correspondence between observed and counterfactual time-series, two additional indicators are used. First, we consider the *mean absolute deviation*, denoted $\left|e\right|$, between the observed and the simulated time-series of SPPR1 and SMAC1 derived from hazard models 0 to 12. Second, as zero-order correlations between time-series showing similar trends are typically high, we calculate the *correlation between first differences of both time-series*, denoted ${r}_{{\text{dif}}}$, reflecting the correlation between year-to-year change in both time-series (Stock & Watson, 2015).

## Results

### Descriptive Results

**Fig. 2:**
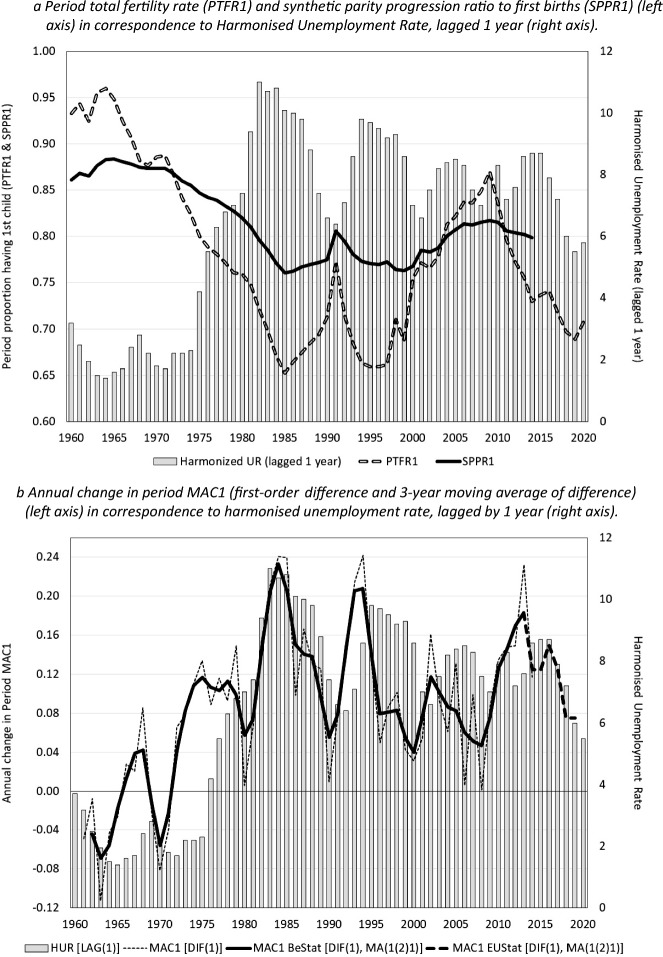
Tempo and quantum of first births in relation to harmonised unemployment rate, Belgium, 1960–2020. **a** Period total fertility rate (PTFR1) and synthetic parity progression ratio to a first birth (SPPR1) (left axis) in correspondence to Harmonised Unemployment Rate, lagged 1 year (right axis). **b** Annual change in period MAC1 (first-order difference and 3-year moving average of difference) (left axis) in correspondence to harmonised unemployment rate, lagged by 1 year (right axis). *Sources*: Statistics Belgium (PTFR1, SPPR1 and PMAC1 estimated retrospectively by authors from the 2001 and 2011 Belgian censuses and population registers 2011–2014), EUROSTAT (PTFR1 & PMAC1) and National Bank of Belgium (Harmonized Unemployment Rate)

Figure 2a plots the period TFR for first births (${\text{PTFR}}1$) and the synthetic parity progression ratio to a first birth (${\text{SPPR}}1$) against the harmonized unemployment rate between 1960 and 2020 (lagged by 1 year). The period TFR1 reached a peak value in 1964 with 95 per cent of the women supposedly having a first child under the prevailing age-specific birth rates, and subsequently declined to a trough in 1985 with only 65 per cent of the women having a first child. Apart from a temporary recovery to 75 per cent in 1991 these low PTFR1 values persisted well into the second half of the 1990s. After 1997, the period TFR1 recovered to a considerably higher value of 0.85 around 2008, but again dropped to a lower value of 70 per cent following the Great Recession of 2009.

The period TFR1 is sensitive, however, to changes in the mean age at first birth over the period considered, suffering inflation when first births are advanced to younger ages and deflation in periods of (accelerating) postponement of parenthood (Bongaarts & Feeney, 1998). The synthetic parity progression ratio—a life-table based estimate that relates first births to the appropriate risk set of nulliparous women—is more robust to changes in the tempo of fertility and provides a closer approximation of cohort fertility trends than the period TFR (Feeney & Yu, 1987; Neels, 2006; Neels & Gadeyne, 2010; Ni Bhrolchain, 1987). Figure 2b plots changes in the *mean age at first birth* in correspondence to the harmonised unemployment rate between 1960 and 2020 (lagged by 1 year). Throughout the 1960s and up to the early 1970s the mean age at first birth declined—barring a stagnation and even slight increase between 1966 and 1968—coinciding with historically low unemployment levels in the period considered. In contrast, the increase of unemployment between 1975 and 1985 was accompanied by accelerating postponement of fertility and severe deflation of the period TFR1 relative to SPPR1. Following a short lived economic recovery and a temporary deceleration of postponement in 1991—resulting in a temporary recovery of SPPR1—fertility postponement again accelerated with the onset of the 1990s recession, although postponement slowed down in the second part of the 1990s despite unemployment levels remaining relatively high until 1999. A similar pattern is found between 2000 and 2008, where the pace of postponement initially accelerated with the increase of unemployment in 2000, but slowed down again despite unemployment remaining high until the mid-2000s, resulting in an inflation of the period TFR1 relative to SPPR1. The onset of the Great Recession in 2009 induced acceleration of fertility postponement between 2009 and 2015, resulting in a strong deflation of the period TFR1 relative to SPPR1. Although SPPR1 is also subject to period variation corresponding to economic cycles over the period considered—with values fluctuating between 77 and 87 per cent of women entering parenthood when subject to the decremental first birth rates in the year considered—the SPPR1 is clearly less affected by changes in the tempo of fertility than the period TFR1.

Zero-order cross-correlations were calculated to assess the correspondence between aggregate-level time-series of fertility indicators and economic cycles. Between 1960 and 2014, the correlation between the harmonized unemployment rate (lagged by one year) and period TFR1 amounts to -0.837, but the correlation is even stronger with SPPR1 standing at -0.938. The correlation between first differences of the time-series of the harmonized unemployment rate and SPPR1 amounts to -0.481, indicating that there is also a moderately strong correlation between the year-to-year variation in both time-series. Similar to previous findings, the cross-correlations between SPPR1 and other economic indicators are considerably lower: $$r=-0.503$$ with GDP (adjusted to 2022 US$), $$r=-0.468$$ with GDP growth and $$r=-0.735$$ with variation in consumer price index (CPI), particularly when considering the correlations between first differences in both time-series (correlations of 0.128, 0.160 and − 0.290 respectively). Although the zero-order correlations suggest that unemployment is the strongest predictor of fertility trends, cross-correlations between aggregate-level time-series do not allow to address the association between age-specific birth hazards and (lagged) indicators of economic context while accounting for the effect of educational expansion on the tempo and quantum of first births.

### Hazard Models and Microsimulation of Aggregate Fertility Indicators

The section on multivariate results covers three large blocks: (i) models considering the effect of educational expansion on tempo (SMAC1) and quantum (SPPR1) of entry into parenthood, (ii) models considering the effect of variation in economic context on entry into parenthood, and (iii) models comparing the effect of variation in unemployment on entry into parenthood across subsequent periods of recession.

### Educational Expansion and Entry into Parenthood

Model 1 includes the set of indicators that capture the educational expansion over the period considered: the time-varying dummy variable for enrolment in education, the time-varying level of education upon leaving education and the duration since leaving education (quadratic specification). The inclusion of indicators related to education entails a significant improvement over the baseline model ($\Delta -2{\text{LL}}=\mathrm{745,954.4}, \Delta df=7, p<.001$), with all educational variables showing the expected effects: enrolment significantly lowers first birth hazards ($exp\left(b\right)=0.235$), while birth hazards after leaving education are a concave function of duration since leaving education ($exp\left(b\right)=1.190$ and $exp\left(b\right)=0.989$ for the linear and quadratic terms respectively). Finally, higher educational attainment is associated with the first birth hazard being increasingly lower compared to women with primary education ($exp\left(b\right)=0.794$, $exp\left(b\right)=0.611$, $exp\left(b\right)=0.567$ and $exp\left(b\right)=0.472$ for lower secondary education, higher secondary education, short type tertiary and long type tertiary education respectively). A second model additionally allowing interaction between educational level and the baseline hazard function (Table 2, Model 2) provides a further improvement in model fit ($\Delta -2{\text{LL}}=\mathrm{120,802.6}, \Delta df=8 , p<.001$).

**Table 2:** Model fit statistics and indicators of correspondence between observed and simulated time-series of SPPR1 and MAC1 as a function of UR, Belgium, 1960–2010 (*N* = 47,354,001 person-years)

Model	Model fit statistics:				Correspondence between time-series					
	-2LL	k	AIC	BIC	SPPR1:			SMAC1:		
					$\left|e\right|$	$r$	${r}_{{\text{dif}}}$	$\left|e\right|$	$r$	${r}_{{\text{dif}}}$
*Block *1* Baseline model*										
M0	17,391,092.6	4	17,391,100.6	17,391,163.3	0.0331	0.382	− 0.080	1.331	− 0.244	0.025
*Block *2* Models including educational expansion*										
M1	16,645,138.2	11	16,645,160.2	16,645,332.6	0.0193	0.846	0.165	0.515	0.971	− 0.049
M2	16,524,335.6	19	16,524,373.6	16,524,671.4	0.0211	0.841	0.196	0.515	0.960	− 0.188
*Block *3* Models including variation in economic context*										
M3	17,275,624.8	5	17,275,634.8	17,275,713.2	0.0277	0.940	0.437	1.037	0.743	0.506
M4	17,223,477.0	11	17,223,499.0	17,223,671.4	0.0131	0.944	0.445	0.866	0.731	0.501
M5	16,483,782.2	26	16,483,834.2	16,484,241.7	0.0093	0.961	0.477	0.448	0.939	0.336
M6	16,494,757.8	24	16,494,805.8	16,495,182.0	0.0120	0.957	0.453	0.456	0.950	0.238
M7	16,470,360.0	54	16,470,468.0	16,471,314.4	0.0097	0.960	0.472	0.438	0.944	0.339
M8	16,478,929.6	30	16,478,989.6	16,479,459.8	0.0121	0.926	0.500	0.369	0.962	0.361
M9	16,465,569.8	74	16,465,717.8	16,466,877.6	0.0121	0.929	0.493	0.363	0.965	0.364
*Block *4* Models allowing period variation in the effect of economic context*										
M10	16,461,877.4	35	16,461,947.4	16,462,496.0	0.0131	0.936	0.340	0.259	0.982	0.358
M11	16,448,614.2	52	16,448,718.2	16,449,533.2	0.0096	0.954	0.532	0.264	0.978	0.389
M12	16,416,859.4	184	16,417,227.4	16,420,111.3	0.0099	0.949	0.525	0.248	0.980	0.398

Column headings refer to: model deviance (-2LL), number of parameters estimated (*k*), Akaike Information criterion (AIC), Bayesian Information Criterion (BIC), mean absolute deviation ($\left|{\text{e}}\right|$), Pearson product-moment correlation (*r*) and Pearson product moment correlation after first-order differencing (${r}_{{\text{dif}}}$)

Source: Belgian censuses of 2001 and 2011, calculations by authors

**Fig. 3:**
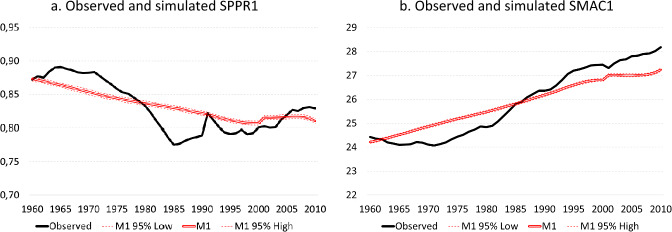
Educational expansion and entry into parenthood: observed and simulated SPPR1 and SMAC1, Model 1, Belgium, 1960–2010. **a** Observed and simulated SPPR1, **b** Observed and simulated SMAC1. Source: Longitudinal microdata from the 2001 and 2011 Belgian censuses, calculations by authors

Although tests indicate that the indicators with respect to the length and outcomes of women’s educational careers have a significant effect on their age-specific hazards of entering parenthood, it is unclear whether and to what extent these indicators effectively account for temporal variation in macro-level indicators such as ${\text{SPPR}}1$ and ${\text{SMAC}}1$. Figure 3 plots the observed time-series of ${\text{SPPR}}1$ and ${\text{SMAC}}1$ for the period 1960–2010, as well as the simulations based on Model 1. Similar to the observed trend, the simulated ${\text{SPPR}}1$ shows a decline in the proportion of women entering parenthood from 87.0 per cent in 1960 to 80.2 per cent in 2000 (Fig. 3a). Unlike the observed trend, however, the simulated ${\text{SPPR}}1$ suggest a steady decline reflecting the gradual expansion of education in the period considered, whereas in reality ${\text{SPPR}}1$ did not decline much between 1960 and 1970, showed a much more rapid decline between 1970 and the mid 1980s than suggested by the model allowing for educational expansion, and even a slight recovery between 1984 and 2000 with a temporary high in the early 1990s. In sum, educational expansion correctly accounts for the gradual decline in ${\text{SPPR}}1$, but fails to account for the observed accelerations and decelerations of this decline over the period considered. This also shows from the indicators measuring the correspondence between time-series. The observed and simulated time-series show a strong correlation owing to the declining trend ($r=.846$), but the correlation drops to a more modest value after first-order differencing (${r}_{{\text{dif}}}=.165$ for Model 1). Although prolonged enrolment in education has been suggested as one of the pathways though which variation in economic context may affect entry into parenthood, the results show that a model incorporating time-varying indicators for enrolment in education is not capable of capturing the accelerations and decelerations in the postponement of parenthood throughout the period considered. Despite the lack of a close correspondence between observed and simulated time-series in terms of year-to-year variation, the model allowing for educational expansion provides a better prediction of ${\text{SPPR}}1$ than the baseline model, with the mean annual absolute deviation $\left|e\right|$ dropping from 3.3 to 1.9 percentage points.

For ${\text{SMAC}}1$ the conclusions are similar to those for ${\text{SPPR}}1$. The models allowing for increased enrolment in education suggest a gradual increase in the mean age at entry into parenthood from 24.0 in 1960 to 27.4 in 2000 (Fig. 3b), but in reality the age at entry into parenthood continued declining throughout most of the 1960s until the early 1970s, after which fertility postponement took off at a much faster pace than suggested by Models 1 and 2. Despite deviations between both time-series, inclusion of the educational variables entails a considerable reduction in the mean annual absolute deviation—from a deviation exceeding 1.3 year in the baseline model to a deviation around half a year in Models 1 and 2 (Table 2)—but correspondence between year-to-year variations in both time-series is limited after detrending (${r}_{{\text{dif}}}$ equal to − 0.049 and − 0.188 in Models 1 and 2 respectively).

### Recession Induced Postponement and Recuperation

The models incorporating variation in the length of educational careers and the associated educational outcomes capture the structural increase of enrolment in education over time, as well as potential educational responses to period variation in economic conditions (e.g. prolonged enrolment in education during economic recessions), but clearly these models fail to provide an accurate account of period variation in both the proportion of women entering parenthood, as well as accelerations and decelerations in the trend of delayed parenthood. As a result, Models 3–9 additionally include temporal variation in unemployment rates, considering differential effects by age-group, level of education and different time lags of the effect.

**Fig. 4:**
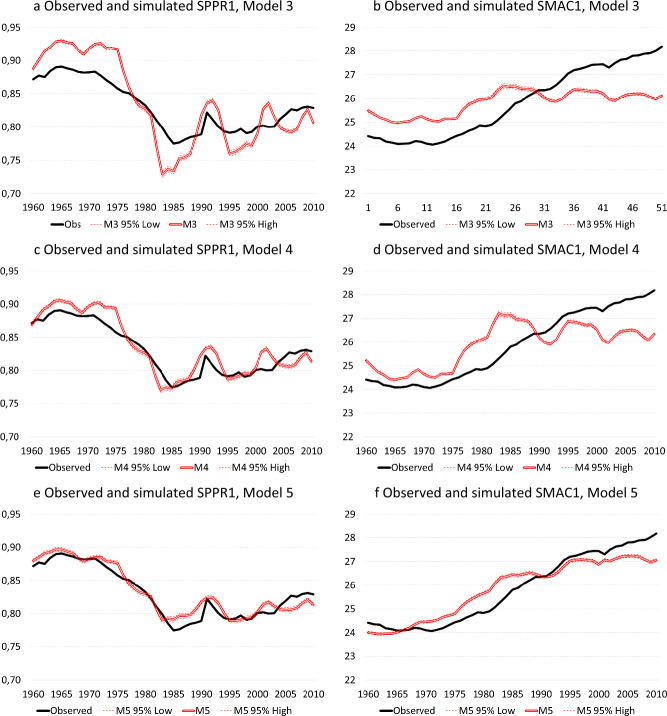
Educational expansion, economic context and entry into parenthood: observed and simulated SPPR1 and SMAC1, Models 3-5, Belgium, 1960–2010. **a** Observed and simulated SPPR1, Model 3. **b** Observed and simulated SMAC1, Model 3. **c** Observed and simulated SPPR1, Model 4, **d** Observed and simulated SMAC1, Model 4, **e** Observed and simulated SPPR1, Model 5. **f** Observed and simulated SMAC1, Model 5. Source: Longitudinal microdata from the 2001 Belgian censuses, calculations by authors

Model 3 only includes the harmonized unemployment rate with a lag of one 1 year, which constitutes a significant improvement over the baseline model ($\Delta -2{\text{LL}}=\mathrm{115,467.8}$, $p\le .001$). According to the model, a one percentage point increase in the unemployment rate reduces the hazard of having a first child by 7.2 per cent in the subsequent year (${\text{exp}}(b)=.928$). Model 3 introduces period variation in SPPR1 corresponding to economic cycles (Fig. 4a), but clearly the model overestimates the period variation induced by economic context compared to the observed time-series of SPPR1 as the model constrains the fertility behaviour of all women to be equally responsive to economic context regardless of age. The mean absolute deviation between the observed time-series and the time-series simulated under model 3 amounts to 0.0277 (an average annual prediction error of 2.77 percentage points in the proportion of women having a first child), whereas the correlation between first differences of both time-series amounts to 0.437 (Table 2). Model 3 performs more poorly with respect to ${\text{SMAC}}1$: the model captures period variation in the pace of postponement that is related to economic cycles, but largely fails to capture the monotonic increase in SMAC1 that is related to educational expansion (Fig. 4b). This is reflected in the indicators of correspondence between the observed and simulated time-series: The mean absolute deviation averages out to a sizeable annual prediction error of 1.04 years, whereas the correlation between year-to-year variation in both time-series—reflecting acceleration/deceleration of postponement in response to unemployment rates—amounts to 0.506 (Table 2).

Model 4 incorporates unemployment rates with a lag of one year similar to Model 3, but allows the effect to vary over subsequent five-year age groups between ages 15 and 49. Model 4 constitutes a significant improvement over Model 3 ($\Delta -2{\text{LL}}=\mathrm{52,147.8}$, $\Delta {\text{d}}f=6$, $p\le .001$), showing that the effect of recession on first birth hazards is negative and procyclical among women under age 35, with the negative effect ranging from $exp\left(b\right)=0.882$ among women aged 15–19 to $exp\left(b\right)=0.988$ among women aged 30–34. Among women aged 35 and older the effect is counter-cyclical among women ages 35–39 ($exp\left(b\right)=1.030$) and 40–44 ($exp\left(b\right)=1.005$), while turning negative again among women aged 45–49 ($exp\left(b\right)=0.792$). Allowing the effect of economic context to differ by age group reduces the mean absolute deviation between the observed time-series of SPPR1 and the series simulated under Model 4 to 0.0131 (an average annual prediction error of 1.3 percentage points in the proportion of women having a first child), whereas the correlation between both time-series equals 0.445 after first-order differencing (Table 2 and Fig. 4c). Model 4 still performs poorly with respect to the mean age at first birth: the model captures year-to-year variation in SMAC1 related to economic cycles (Fig. 4d), but again fails to capture the gradual increase in SMAC1 since 1972 induced by educational expansion (Fig. 4b). The mean absolute deviation between the observed time-series of SMAC1 and the time-series simulated under Model 4 equals 0.866 years, whereas the correlation between first differences of both series equals 0.501, similar to Model 3 (Table 2).

Model 5 combines the effect of educational expansion (enrolment in education, educational attainment, duration since leaving with education and the interaction between the baseline and level of education) with the effect of unemployment lagged by 1 year and differentiated by age, resulting in a significant improvement compared to both Model 2 ($\Delta -2{\text{LL}}=\mathrm{40,553.4}, \Delta {\text{d}}f=7, p\le .001$) and Model 4 ($\Delta -2{\text{LL}}=\mathrm{739,694.8}, \Delta {\text{d}}f=15, p\le .001$). The absolute mean deviation between the observed time-series of SPPR1 and the simulated time-series under Model 5 has declined to 0.0093 (an average annual prediction error below 1 percentage point in the proportion of women having a first child), while the correlation between first differences of both series amounts to 0.477 (Table 2 and Fig. 4e). With respect to ${\text{SMAC}}1$, the mean average deviation between the observed time-series and the time-series simulated under Model 5 declines to 0.448, whereas the correlation between first differences of both time-series is somewhat lower than Model 4 at 0.336. Although Model 5 captures the structural increase in SMAC1, as well as acceleration/deceleration of postponement induced by economic cycles, the simulated time-series overestimates the pace of postponement between 1975 and 1985, while underestimating the pace of postponement after 1990.

Model 6 considers the effect of educational expansion and allows the effect of unemployment (lagged by one year) to differ by level of education rather than age. Model 6 results in a significant improvement over Model 2 ($\Delta -2{\text{LL}}=\mathrm{29,577.8}, \Delta {\text{d}}f=5, p\le .001$), but the absolute mean deviation between observed and simulated time-series is somewhat larger than for model 5, both for SPPR1 (0.0120 vs 0.0096 under Model 5) and SMAC1 (0.4560 vs 0.4481 under Model 5). Also correlations between first differences of observed and simulated time series are lower for Model 6 than Model 5, both for SPPR1 (0.453 vs 0.477 under Model 5) and SMAC1 (0.238 vs 0.336 under Model 5). Model 7 additionally considers the 3-way interaction between unemployment (lagged by one year), age and level of education. While significantly outperforming Model 5 ($\Delta -2{\text{LL}}=\mathrm{13,422.2}, \Delta {\text{d}}f=28, p\le .001$) and Model 6 ($\Delta -2{\text{LL}}=\mathrm{24,397.8}, \Delta {\text{d}}f=30, p\le .001$) in terms of model fit, the mean absolute deviation and the correlation between first differences of observed and simulated time-series of SPPR1 and SMAC1 do not generally outperform Model 5, making the latter model preferable for reasons of parsimony.

**Fig. 5:**
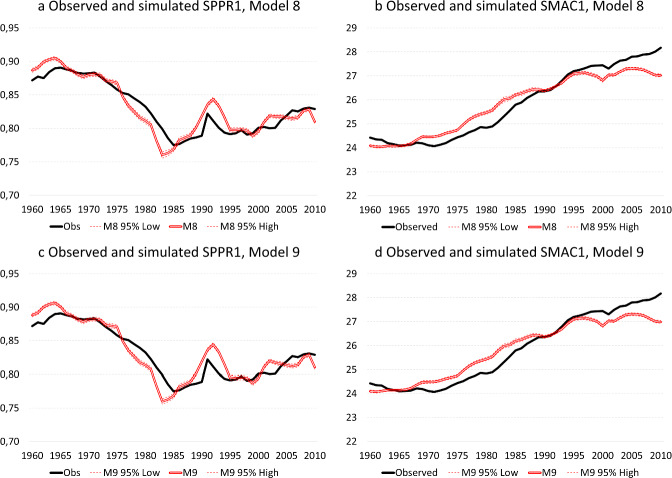
Educational expansion, economic context and entry into parenthood: observed and simulated SPPR1 and SMAC1, Models 8-9, Belgium, 1960–2010. **a** Observed and simulated SPPR1, Model 8. **b** Observed and simulated SMAC1, Model 8. **c** Observed and simulated SPPR1, Model 9. **d** Observed and simulated SMAC1, Model 9. Source: Longitudinal microdata from the 2001 and 2011 Belgian censuses, calculations by authors

Model 8 introduces a recuperation effect into Model 5 by allowing first birth hazards after the age of 30 to compensate for the economic cycles experienced earlier in the life course with a lag of 10 years. Although Model 8 constitutes a significant improvement over Model 5 ($\Delta -2{\text{LL}}=\mathrm{4,852.6}, \Delta {\text{d}}f=4, p\le .001$) and the correlation of 0.500 between first-order differences of the observed and simulates time-series of SPPR1 is higher than for Model 5, the mean absolute deviation between both time-series is again slightly higher at 0.0121. With respect to ${\text{SMAC}}1$, the mean absolute deviation between the observed and the simulated time-series under Model 8 decreases to 0.369 years, while the correlation between first-order differences of both time-series increases to 0.361. Compared to Model 5 the average annual prediction error has decreased and the correlation between year-to-year changes in both time-series has strengthened, but the pace of fertility postponement is still somewhat overestimated between 1975 and 1985, while the acceleration of postponement is underestimated after the mid-1990s (Fig. 5b). Model 9 additionally considers the three-way interactions between age, level of education and the harmonized unemployment rate at lags of 1 and 10 years, respectively (Fig. 5c and d). Although the model is a significant improvement over Model 8 ($\Delta -2{\text{LL}}=\mathrm{13,359.8}, \Delta {\text{d}}f=44, p\le .001$), the performance of Model 9 is similar to that Model 8 in terms of simulating macro-level time-series of both SPPR1 and SMAC1.

### Recession and Entry into Parenthood: has the Association Changed Over Time?

**Fig. 6:**
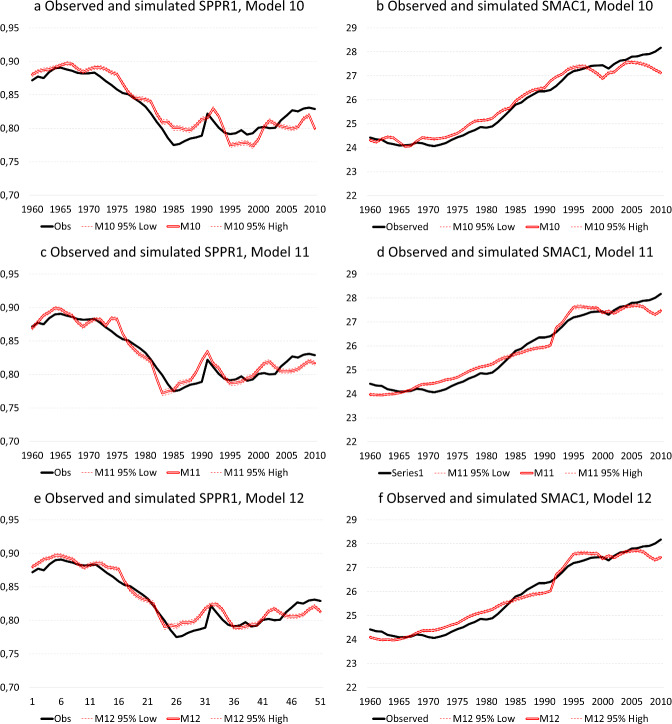
Educational expansion, economic context and entry into parenthood: observed and simulated SPPR1 and SMAC1, Models 10-12, Belgium, 1960–2010. **a** Observed and simulated SPPR1, Model 10. **b** Observed and simulated SMAC1, Model 10. **c** Observed and simulated SPPR1, Model 11. **d** Observed and simulated SMAC1, Model 11. **e** Observed and simulated SPPR1, Model 12. **f** Observed and simulated SMAC1, Model 12. Source: Longitudinal microdata from the 2001 and 2011 Belgian censuses, calculations by authors

Finally, Models 10, 11 and 12 address the question whether the effect of economic cycles—as measured under the form of the harmonised unemployment rate—on the proportion of women entering parenthood and the mean age at first birth has changed over successive recession periods, distinguishing the periods 1960–1973, 1974–1991 and 1992–2010. Whereas Model 10 considers two-way interactions between period and the harmonized unemployment rate at lags of 1 and 10 years, Model 11 considers three-way interactions between period, age (in 5-year age-groups) and the harmonized unemployment rate (at lags of 1 and 10 years) and Model 12 considers four-way interactions between period, age, level of education and the harmonized unemployment rate (at lags of 1 and 10 years). Although Model 10 constitutes a significant improvement over Model 8 ($\Delta -2{\text{LL}}=\mathrm{17,052.2}, \Delta {\text{d}}f=5, p\le .001$), the mean absolute deviation between the observed and simulated time-series of SPPR1 is larger than for models omitting period variation in the effect of economic cycles (cf. $\left|e\right|=0.0131$ in Model 10 versus $\left|e\right|=0.0093$ in Model 5) and also the correlation between first differences of both time-series is weaker (cf. $r=0.340$ in Model 10 versus $r=0.500$ in Model 8) (Table 2 and Fig. 6a). This holds regardless of whether the fit between observed and simulated time-series is considered over the entire observation period or whether successive recession periods are considered separately (Table 3). With respect to the mean age at first birth, the mean absolute deviation in Model 10 ($\left|e\right|=0.259$) outperforms models that constrained the effect of economic cycles to be similar across recession periods, whereas the correlation between first-order differences is roughly similar in Model 10 ($r=0.358$) to Model 8 (Table 2 and Fig. 6b).

**Table 3:** Correspondence between observed and simulated time-series of SPPR1 and SMAC1 over successive periods of recession, Belgium, 1960–2010 (*N* = 47.354.001 person-years)

Periods:	Blocks & models												
	I	II		III							IV		
	M0	M1	M2	M3	M4	M5	M6	M7	M8	M9	M10	M11	M12
A. *Mean absolute deviation between observed and simulated time-series of SPPR1, *|*e*|:													
1960–1973	0.0496	0.0212	0.0264	0.0363	0.0137	0.0063	0.0120	0.0075	0.0081	0.0086	0.0079	0.0054	0.0051
1974–1991	0.0299	0.0247	0.0254	0.0274	0.0131	0.0110	0.0103	0.0106	0.0161	0.0156	0.0148	0.0125	0.0131
1992–2010	0.0240	0.0128	0.0132	0.0216	0.0127	0.0098	0.0137	0.0105	0.0112	0.0113	0.0152	0.0100	0.0104
1960–2010	0.0331	0.0193	0.0211	0.0277	0.0131	0.0093	0.0120	0.0097	0.0121	0.0121	0.0131	0.0096	0.0099
B. *Correlation between first differences of observed and simulated time-series of SPPR1, *(${r}_{dif}$):													
1960–1973	0.0268	0.4346	-0.4918	0.5898	0.5856	0.5775	0.5950	0.5832	0.5475	0.5284	0.4749	0.6044	0.5675
1974–1991	-0.0103	-0.3577	-0.3905	0.5146	0.5101	0.4904	0.5159	0.5044	0.5951	0.5948	0.4497	0.5239	0.5396
1992–2010	-0.3898	0.3482	0.5660	0.2458	0.2482	0.3702	0.3117	0.3427	0.2977	0.2941	0.2410	0.5141	0.4798
1960–2010	-0.0802	0.1651	0.1958	0.4374	0.4451	0.4774	0.4532	0.4721	0.4997	0.4926	0.3395	0.5323	0.5253
C. *Mean absolute deviation between observed and simulated time-series of SMAC1, *|*e*|:													
1960–1973	1.7145	0.4791	0.4661	0.9488	0.4764	0.2724	0.3096	0.2726	0.2238	0.2263	0.1720	0.2469	0.2121
1974–1991	0.7665	0.4099	0.4182	0.7919	0.9546	0.5561	0.4921	0.5299	0.4153	0.3975	0.2442	0.2833	0.2852
1992–2010	1.5840	0.6411	0.6426	1.3345	1.0680	0.4753	0.5297	0.4738	0.4316	0.4313	0.3357	0.2583	0.2401
1960–2010	1.3313	0.5150	0.5149	1.0371	0.8656	0.4481	0.4560	0.4384	0.3688	0.3631	0.2585	0.2640	0.2483
D. *Correlation between first differences of observed and simulated time-series of SMAC1, *(${r}_{dif}$):													
1960–1973	-0.0820	0.5668	0.5892	0.7145	0.7058	0.7067	0.7029	0.6971	0.6332	0.6134	0.2592	0.6725	0.6485
1974–1991	-0.1218	0.0739	0.3215	0.2163	0.2512	0.2861	0.2590	0.3212	0.3564	0.3917	0.3563	0.3900	0.3088
1992–2010	0.2405	-0.0941	-0.2880	0.7033	0.6899	0.3094	0.1332	0.3357	0.2443	0.2713	0.2012	0.4806	0.4432
1960–2010	0.0247	-0.0495	-0.1885	0.5056	0.5011	0.3361	0.2377	0.3392	0.3610	0.3637	0.3583	0.3887	0.3982

Source: Longitudinal microdata from the 2001 and 2011 Belgian censuses, calculations by authors

The inclusion of three-way interactions in Model 11 constitutes a significant improvement over Model 10 ($\Delta -2{\text{LL}}=\mathrm{13,263.2}, \Delta {\text{df}}=17,\mathrm{ p}\le .001$) (Table 2 and Fig. 6c and d). The correlation between first differences of the observed and the simulated time-series of SPPR1 is slightly higher under Model 11 (${r}_{dif}=0.532$) than for previous models, which holds for both the entire observation period as for successive recession periods (Table 3). In contrast, the mean absolute deviation between observed and simulated time-series of SPPR1 is similar in Model 11 ($\left|e\right|=0.0096$) compared to Model 5. With respect to the mean age at first birth, the absolute mean deviation between observed and simulated time-series of SMAC1 for Model 11 ($\left|e\right|=0.264$) is similar to Model 10, while the correlation between first differences of the observed and simulated time-series of SMAC1 is similar to that of models assuming the effect of economic cycles on entry into parenthood to be similar over successive recession periods (e.g. Model 8) (Table 2).

Finally, Model 12 (Figs. 6e and f) that considers the four-way interactions between period, age, level of education and harmonized unemployment rate (at lags of 1 and 10 years) constitutes a significant improvement over Model 11, but the mean absolute deviation between observed and simulated time-series of SPPR1 ($\left|e\right|=0.0099$) is nevertheless similar to that in Model 5, although the correlation between first differences of observed and simulated time-series (${r}_{dif}=.525$) is somewhat higher than in Models 5 and 8 (Table 2). With respect to ${\text{SMAC}}1$, the mean absolute deviation between observed and simulated time-series under Model 12 ($\left|e\right|=.248$) is lower than in models that assume the effect of economic cycles to be constant over time, although the correlation between first differences of SMAC1 is only slightly higher than in Models 5 and 8 (Table 2).

### Sensitivity Analyses

The analyses were replicated using consumer price index (CPI, Models 3–12 in Table A.2) and gross domestic product (GDP, Models 3–7 in Table A.3 only because a harmonized time-series of GDP was not available prior to 1960) as indicators of economic context. Although the cyclical component of different macroeconomic time series may lead or lag the business cycle by intervals of varying length (Hodrick & Prescott, 1997), lags of 1 and 10 years were maintained for the sake of comparison. Models using GDP show larger mean absolute deviations between observed and simulated time-series than models using the harmonised unemployment rate (UR) and also correlations between first differences of observed and simulated time-series are substantially lower, both for SPPR1 and SMAC1, making these models less performant (Figs. A.5 and A.6 in annex). Models using CPI also show larger mean absolute deviations between observed and simulated time-series of SPPR1 for models M3-M7, and similar mean absolute deviations for models M8-M12, but correlations between first differences of observed and simulated time-series are generally lower for models using CPI than for models using UR. In contrast, mean absolute deviations between observed and simulated time-series of SMAC1 are somewhat lower for models using CPI than models using UR, but correlations between first differences of observed and simulated time-series are substantially higher for models M5-M12 using UR.

## Discussion and Conclusion

In his two-stage perspective on demographic inquiry Billari argues that explaining macro-level demographic change requires i) recognizing that demographic events are driven by human (inter)actions, embedded in their macro-level context, and ii) specifying how macro-level population patterns re-emerge from the (inter)action of individual life courses (Billari, 2015). Several factors have been suggested to explain the trend to later childbearing and a sizeable body of literature has documented the associations between these factors and fertility outcomes at the individual level (first stage), but only a limited number of studies have attempted to quantify the contribution of these factors to macro-level fertility trends (second stage). Previous studies considering the effect of educational expansion on timing of entry into parenthood suggest that around 50 percent of the increase in the aggregate-level mean age at first birth between 1970 and 2000 can be accounted for by the lengthening of educational careers and the associated shift in social age where adult roles are assumed later in the life course (Neels & De Wachter, 2010; Neels et al., 2017; Ni Bhrolchain & Beaujouan, 2012), but also show that considerable variation in age-specific first birth hazards over time cannot be accounted for in terms of educational expansion. Based on population-wide longitudinal microdata from the Belgian censuses in tandem with harmonized contextual data on economic conditions, this paper uses hazard and microsimulation models to assess whether and to what extent economic cycles can account for accelerations and decelerations in the macro-level trend of fertility postponement and variation in the annual proportion of women entering parenthood in the period 1960–2010, allowing for educational expansion (Research Question 1). In addition, we test whether the effect of economic cycles on women’s entry into parenthood has changed over subsequent recession periods, and we assess the added value of including period variation in the effect of economic cycles for the prediction of aggregate-level fertility trends (Research Question 2). In addition to standard indicators of model fit, we compare observed and simulated time-series of the synthetic parity progression ratio to a first child (SPPR1) and the synthetic mean age at entry into parenthood (SMAC1) to assess model performance. The mean absolute deviation is used as an indicator of the average annual difference between observed and simulated time-series, whereas the correlation between first differences of observed and simulated time-series provides an indication of the correspondence in year-to-year variation of both time-series.

The results show that year-to-year variation in the proportion of women entering parenthood and acceleration/deceleration in fertility postponement are closely associated with variation in unemployment rates, with predominantly the timing of family formation among women under age 30 being closely associated with economic cycles. Models jointly considering the effects of educational expansion and economic cycles predict synthetic parity progression ratios to a first birth with an accuracy of less than 1 percentage point for the period 1960–2010 (an average error of prediction below 1 per cent in the annual proportion of women entering parenthood), while additionally showing a moderately strong correlation between year-to-year variations in observed and simulated time-series, even when the effect of economic cycles is constrained to be identical across successive recession periods. In addition, the mean age at first birth in each year can be predicted with an accuracy of 0.363 years (approximately 4,3 months) on average, while the correlation between first differences in observed and simulated time-series is somewhat lower for SMAC1 than SPPR1. Considering research question 1, it thus seems vital to consider economic cycles to account for the acceleration of fertility postponement between the early 1970s and the late 1980s, the deceleration in the early 1990s and the acceleration again from the mid 1990s onwards.

In view of answering the second research question, more elaborate models were estimated that include up to four-way interactions between period, age, level of education and the harmonized unemployment rates at lags of 1 and 10 years. Although models allowing period variation in the effect of economic cycles yield a significant improvement in model fit over models that constrain the effect of economic conditions to be constant over time, indicating that there is variation in the age-education-specific effect of economic context on first birth hazards over successive recessions, the improvement is marginal when mean absolute deviations and correlations between first differences of observed and simulated time-series of SPPR1 and SMAC1 are considered at the macro-level. The latter suggests that hazard-based microsimulation models that take into account the effect of recent trends in enrolment in education as well as the effects of economic cycles may be useful to generate accurate short-term estimates of fertility trends. The accuracy of long-term extrapolations of fertility trends is expected to be lower as developments with respect to education and economic cycles which serve as important inputs for the microsimulation model become increasingly uncertain in the long-run.

The results in no way imply that other factors than those considered in this paper are not relevant to explain variation in timing of fertility between individuals. Variation between women in their timing of entry into parenthood is likely associated with a large number of factors at any point in time. However, to the extent that such factors have not been subject to large-scale structural changes or temporal variation over time, they may be less relevant to account for period variation in macro-level parity progression ratios and the acceleration or deceleration of fertility postponement. When focussing on temporal variation in macro-level fertility indicators, our results show that individual-based models allowing for the gradual extension of school careers in tandem with adaptation of timing of parenthood to variation in economic context provide accurate annual estimates of period fertility indicators such as SPPR1 and SMAC1. Although economic and demographic trends in Belgium show considerable communalities with trends in other European countries over the period considered, the specificities of the Belgian policy context—e.g. in terms of level and duration of unemployment benefits (OECD, 2024), employment protection (OECD, 2020) and wage indexation (Geis, 2023)—most likely shape the association between economic cycles, labour market outcomes and fertility responses. Replication of the hazard and microsimulation models developed in this paper for other country contexts can indicate whether and to what extent the findings for the Belgian case apply more generally.

Although the individual-based models discussed in this paper allow accurate predictions of macro-level trends in SPPR1 and SMAC1 over a 50-year observation period, the analyses are subject to several limitations, suggesting avenues for future research. A first limitation concerns the measurement of economic cycles and their impact on income and labour market positions at the individual and household level. The harmonized unemployment rate used in this paper could not be consistently differentiated by age-group or educational level throughout the observation period considered, although unemployment rates in most periods are strongly differentiated in terms of these characteristics (UNIA & Fod WASO, 2017; UNIA & FOD Waso, 2020). The analyses included interactions between the harmonized unemployment rate and individual characteristics (e.g. age and education), but the incorporation of profile-specific unemployment rates would allow to further explore population heterogeneity in the association between economic cycles and fertility. A further enhancement would be to additionally incorporate longitudinal microdata on income and labour market positions of both women and their partners. This would allow to identify mediating mechanisms in the association between macro-level economic indicators and fertility, while explicitly considering (changing) gender dynamics in households under varying economic conditions (Távora & Rodriguez-Modroño, 2018). To the extent that unemployment rates decline as a result of dwindling labour supply in European countries, variation in unemployment rates at the macro level may also become a less accurate proxy of precarious income and employment conditions faced by households, potentially changing the association between macro-level unemployment levels and birth hazards. Particularly in Northern and Western European countries the changing association between economic cycles and fertility trends requires further attention in this respect (Comolli et al., 2021; Matysiak et al., 2021). A second limitation of the analyses presented in this paper is that population heterogeneity in terms of migration background has not been considered, despite the fact that European societies have become increasingly diverse and that income and labour market positions are typically differentiated in terms of migration background in countries across Europe (Andersson & Scott, 2005; Lundstrom & Andersson, 2012; UNIA & Fod WASO, 2017; UNIA & FOD Waso, 2020; Wood & Neels, 2017). Women and/or households with a migration background may be less affected by variation in economic conditions, as they do not have the same anticipated increase in earnings profile (Cigno & Ermisch, 1989; Gustafsson, 2001) or may not consider economic factors to the same extent in the decision to have children (Edin & Kefalas, 2005; Friedman et al., 1994). A third limitation is that the analyses presented in this paper have been confined to first births. Additional research is needed to assess whether and to what extent the hazard-based microsimulation models discussed in this paper can be successfully extended to also include second and higher-order births. Considering successive birth intervals in hazard models for repeated events allows to additionally address issues of population heterogeneity and selection that are not feasible in the analysis of macro-level fertility indicators and trends, while the microsimulation approach presented in this paper provides a straightforward approach to map the aggregate-level implications of the individual-based models considered.

## Notes

In an earlier version of the paper the aggregate-level SPPR1 and SMAC1 time-series corresponding to the different hazard models were calculated directly from the estimated conditional probabilities ${\widehat{q}}_{ti}$:

$${\widehat{SPPR}}_{t}=1-\prod_{a=15}^{49}\left(1-{\widehat{q}(a)}_{t}\right)$$

where $t$ denotes calendar year and $a$ denotes age in period difference. Although the direct calculation provides equivalent results, we prefer to procedure using a random generator which we consider more general as the latter not only allows to calculate aggregate-level fertility indicators that are not based on a synthetic life table, but also allows to generate counterfactual risk sets as well as out-of-sample simulations.

## Supplementary Information

Below is the link to the electronic supplementary material.

Supplementary file1 (DOCX 677 KB)

## Data Availability

The data that support the findings of this study are available from Statistics Belgium but restrictions apply to the availability of these data, which were used under license for the current study, and so are not publicly available.
