# Supplementary material for: Economic Cycles and Entry into Parenthood: Is the Association Changing and Does it Affect Macro-Level Trends? Micro-Level Hazard and Simulation Models of Belgian Fertility Trends, 1960–2010
Source: Eur J Popul. 2024 Mar 29;40(1):13. doi: 10.1007/s10680-024-09695-6 (PMC10980675; doi:10.1007/s10680-024-09695-6)
Supplement: Supplementary file 1 — Supplementary file1 (DOCX 677 KB) [file 10680_2024_9695_MOESM1_ESM.docx]

**ANNEX**

*Figure A.1 Retrospective estimates of the period total fertility rate based on the 2001 and 2011 Belgian censuses and validation against vital registration, Belgium, 1960-2020.*


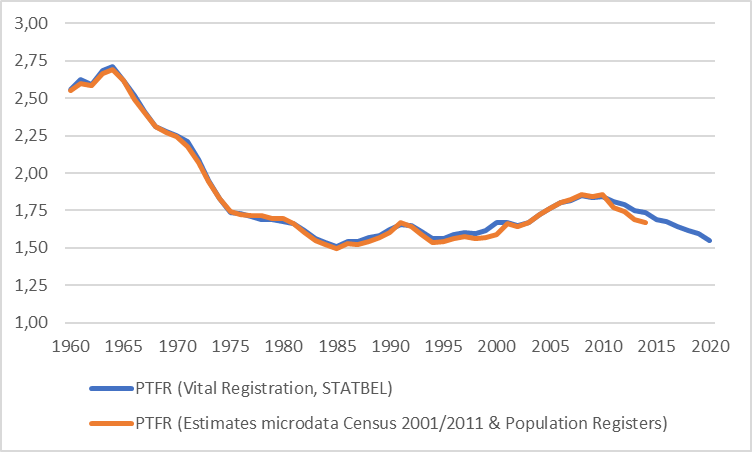


Sources: Retrospective estimation for 1960-2000 based on maternity histories in the 2001 Census, Retrospective estimation for 2001-2010 based on descent data in 2011 Census, prospective estimation for 2011-2014 based on 2011 Census and register follow-up, calculations by authors.

*Figure A.2 Enrolment in education and level of education among nulliparous women aged 15-50, Belgium, 1960-2010.*

| 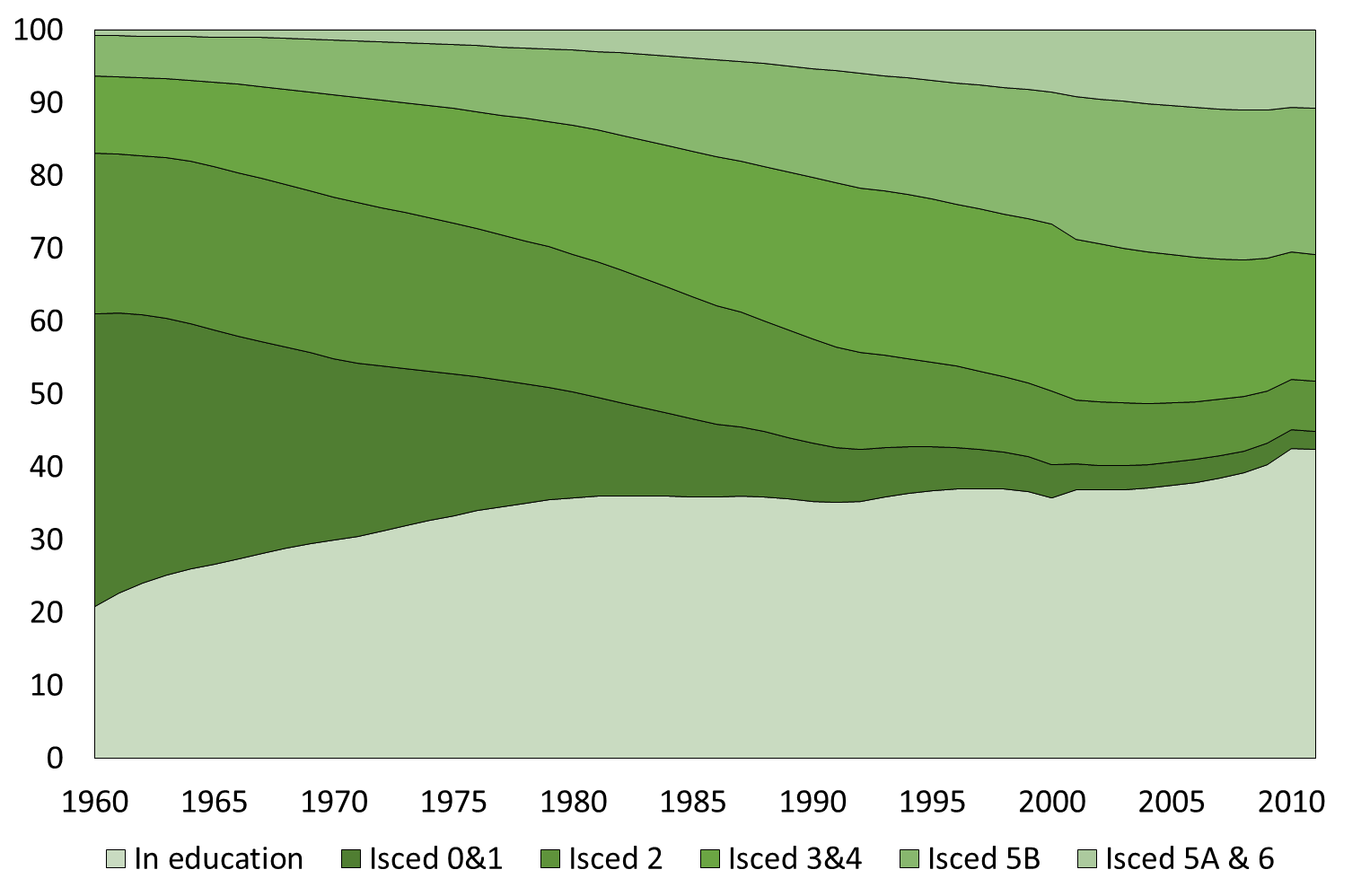 |
| --- |
| Source: Belgian censuses of 2001 and 2011, calculations by authors. |

*Figure A.3 Harmonized unemployment rate by age and sex, Belgium, 1960-2022.*

**
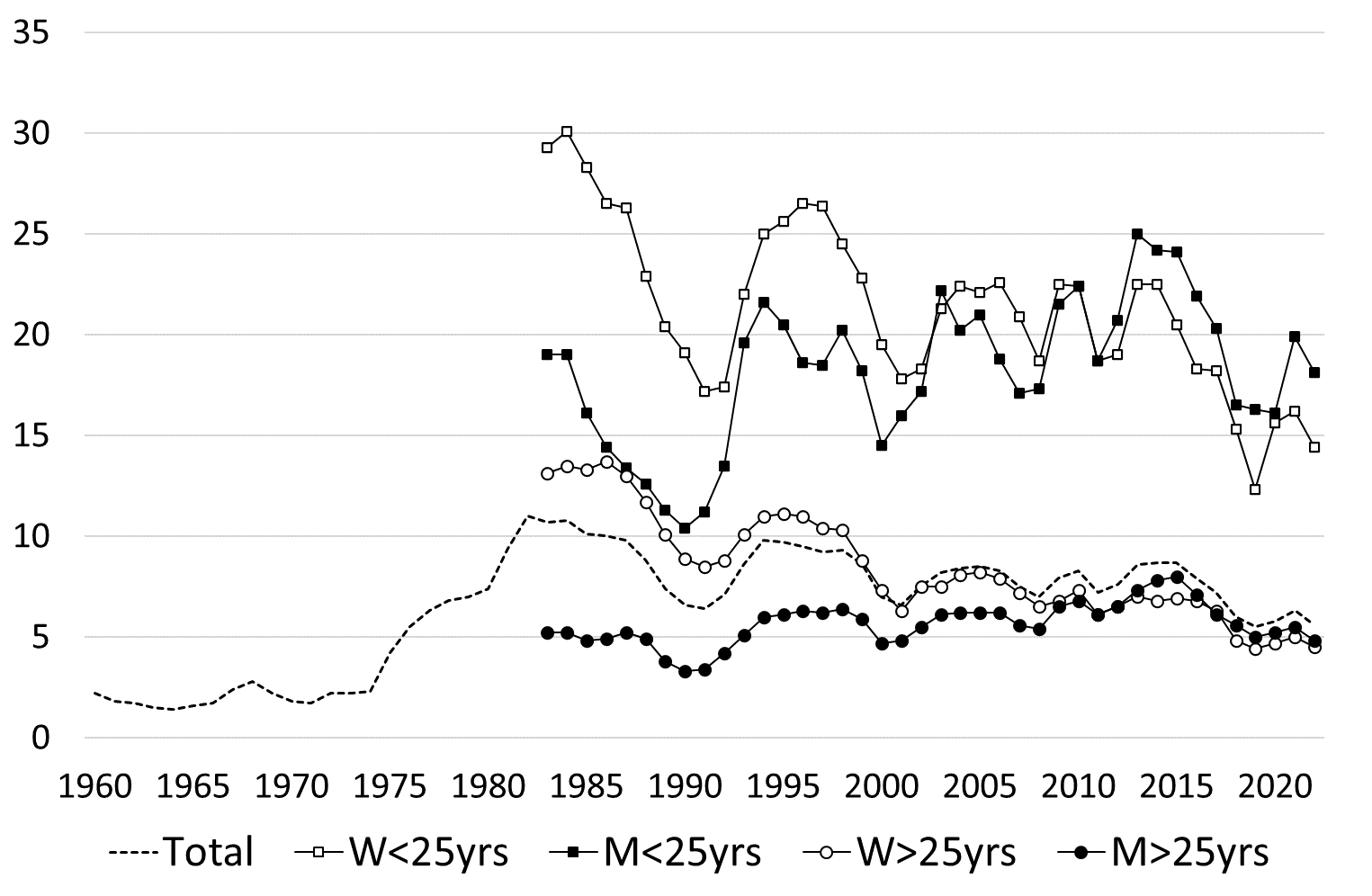
**

Source: Belgium, National Bank (accessed 13/07/2023)

*Figure A.4 Activity rates (top) and inactivity rates (bottom) by gender,*

*Population aged 20-64 years, Belgium, 1983-2022.*

| 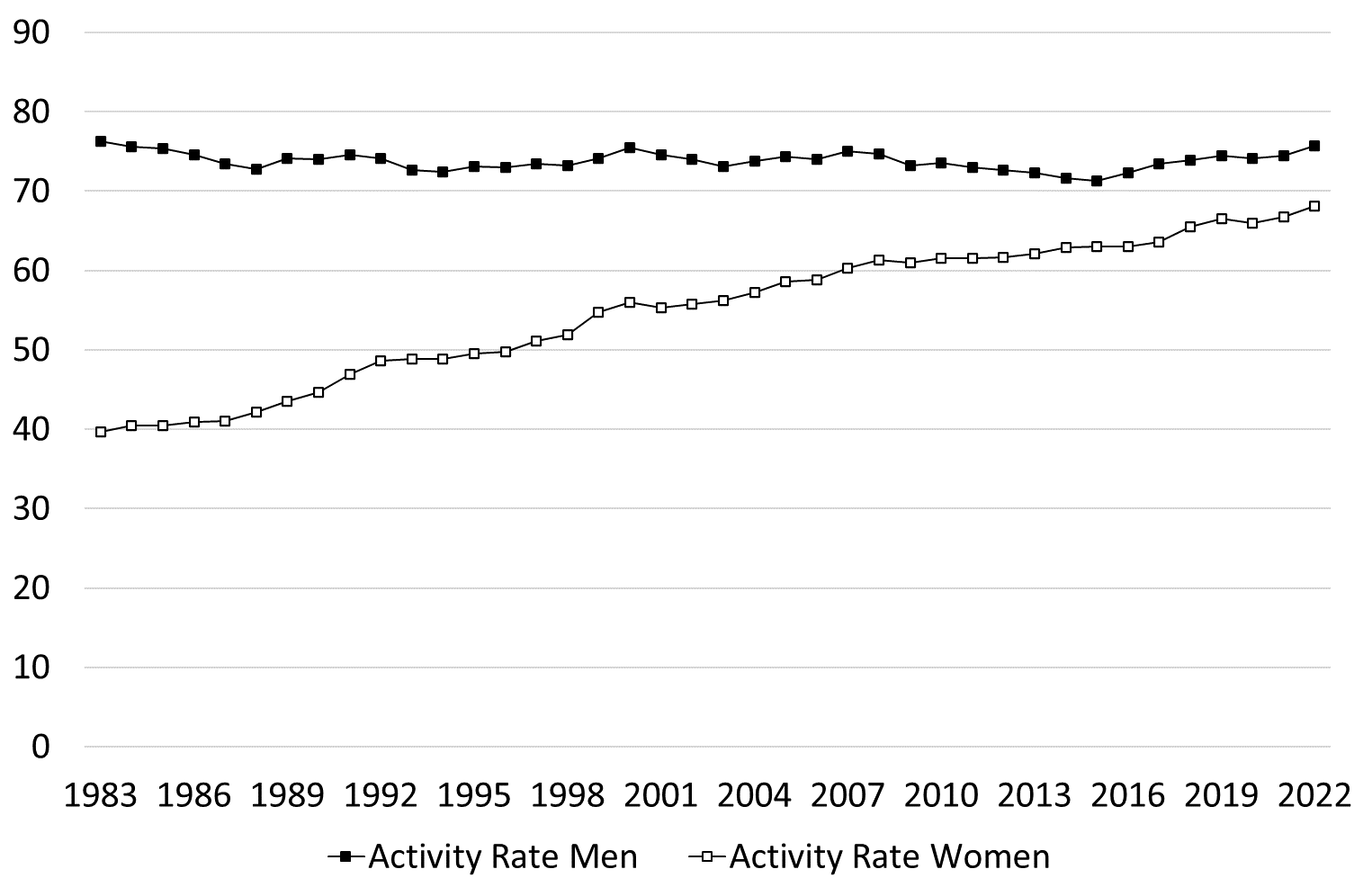 |
| --- |
| 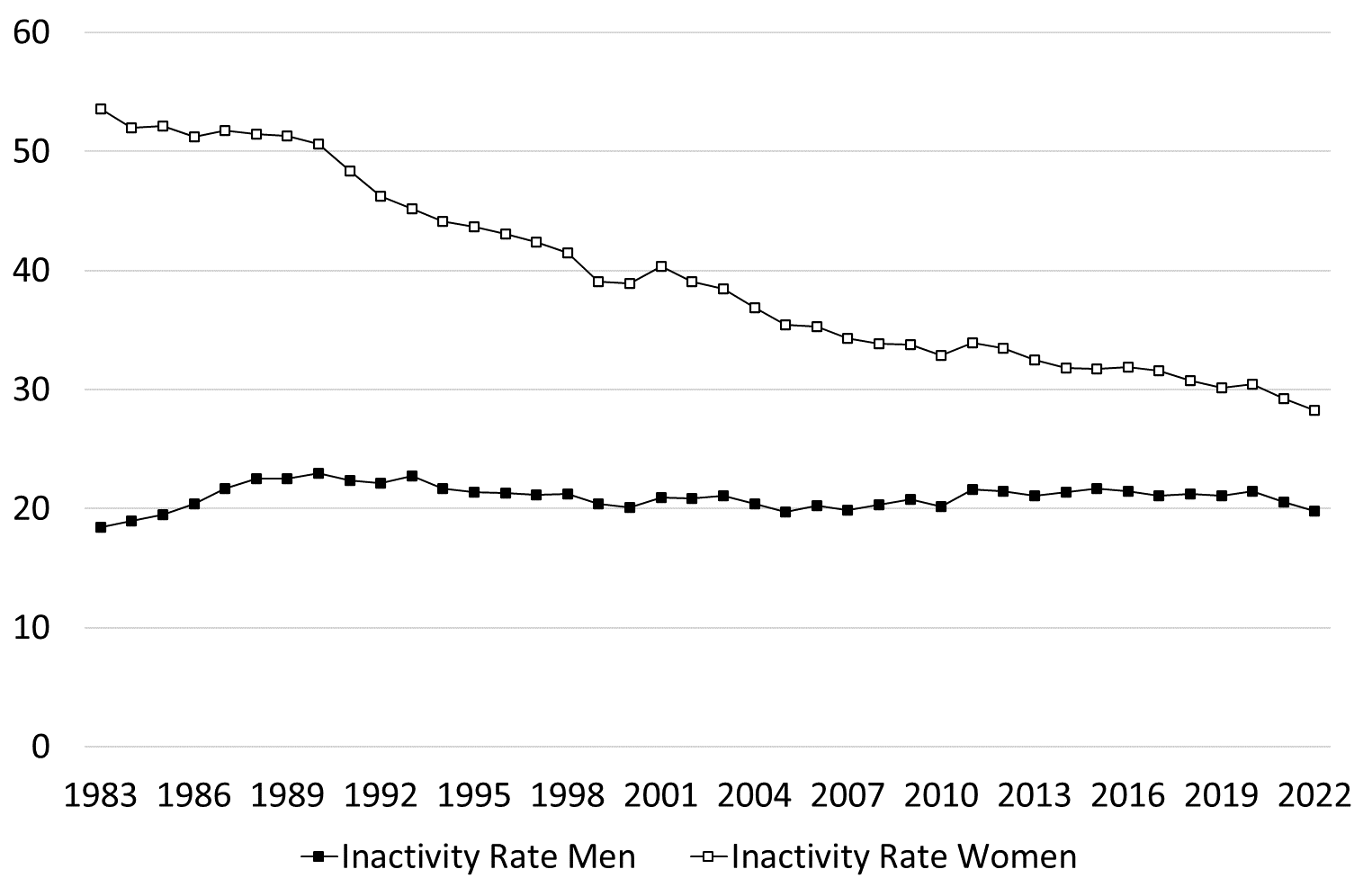 |
| Source: Statistics Belgium, based on Labour Force Surveys (accessed 13/07/2023) |

*Figure A.5 Mean absolute deviation between observed and simulated time-series of SPPR1 (left ordinate) and correlation between first differences of observed and simulated time-series of SPPR1 (right ordinate) using harmonized unemployment rate (UR, Model 0-12), consumer price index (CPI, Models 0-12) and gross domestic product (GDP, Models 0-7) as indicators of economic context, Belgium, 1960-2010.*

| 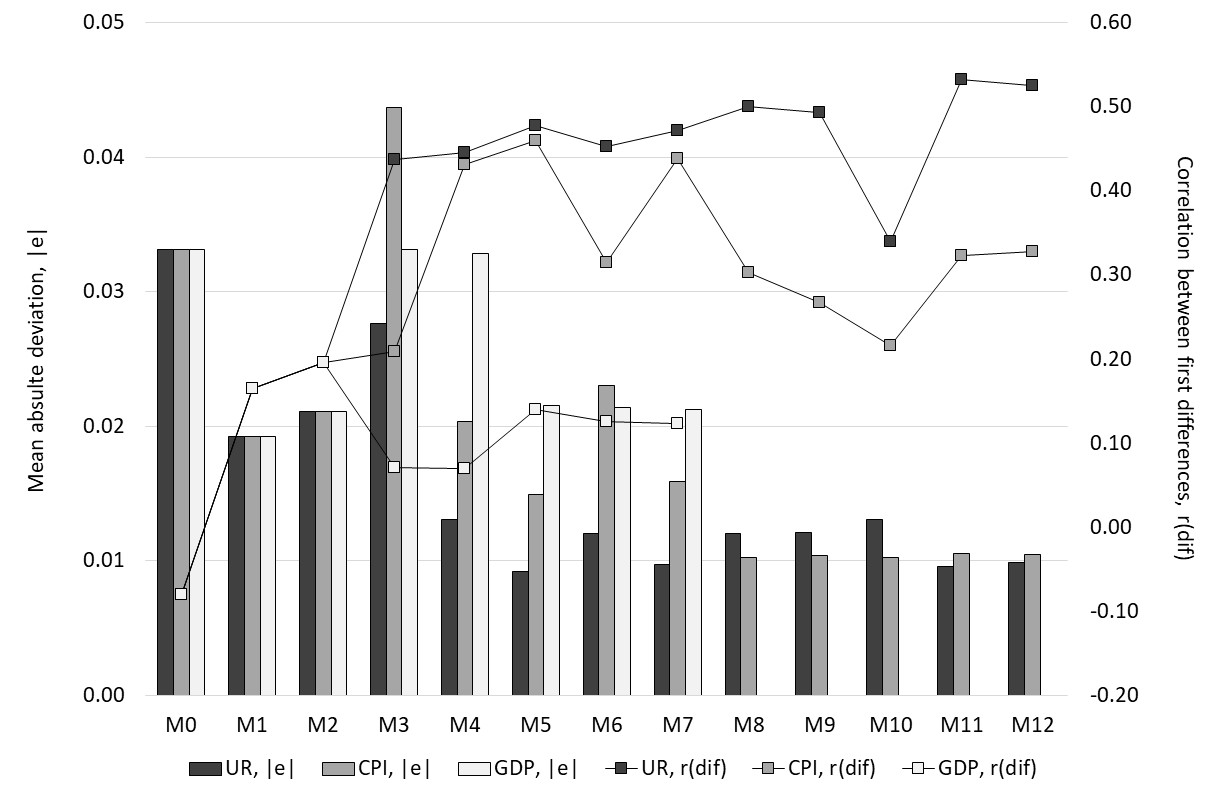 |
| --- |
| Source: Belgian censuses of 2001 and 2011, calculations by authors. |

*Figure A.6 Mean absolute deviation between observed and simulated time-series of MAC1 (left ordinate) and correlation between first differences of observed and simulated time-series of MAC1 (right ordinate) using harmonized unemployment rate (UR, Model 3-12), consumer price index (CPI, Models 3-12) and gross domestic product (GDP, Models 3-7) as indicators of economic context, Belgium, 1960-2010.*

| 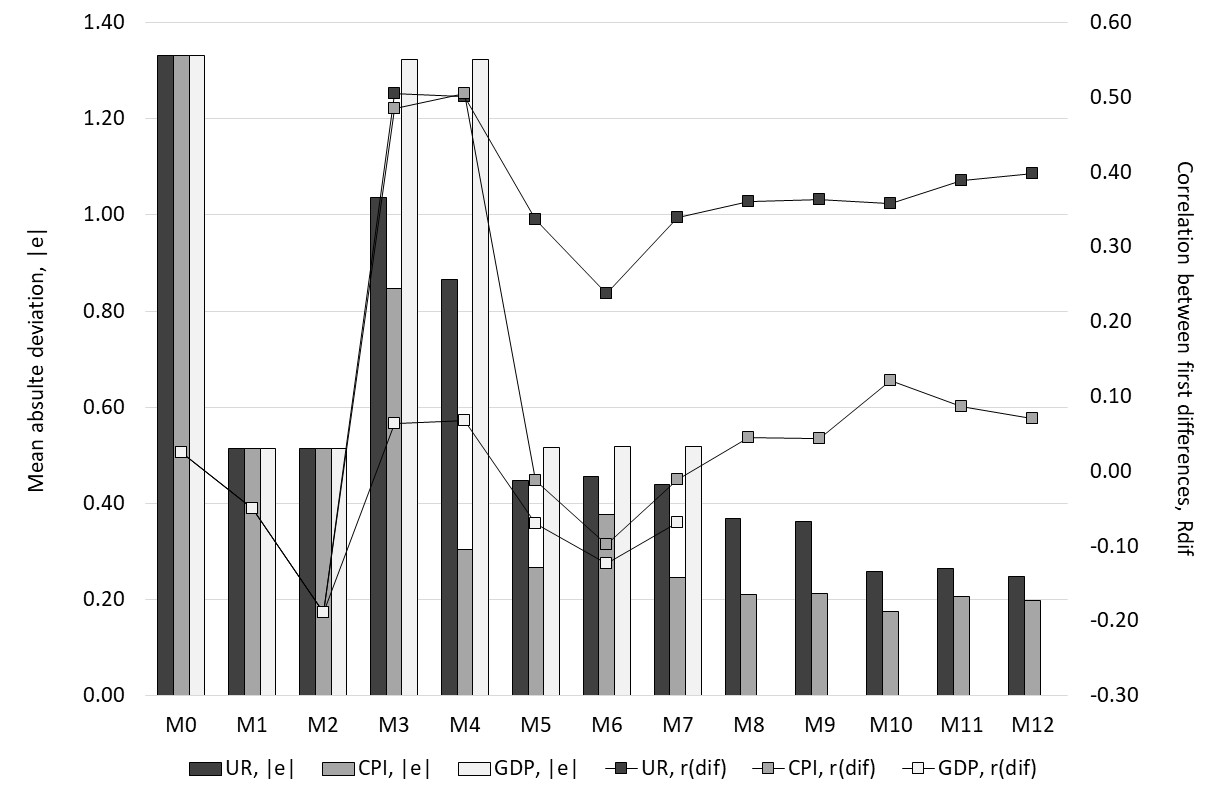 |
| --- |
| Source: Belgian censuses of 2001 and 2011, calculations by authors. |

*Figure A.7 Cross-correlations between time-series of first birth hazards (at various ages) and the harmonized unemployment rate considering different lags, Belgium, 1970-2000.*

| *Cross-correlations between first birth hazards at age 20 and unemployment rate at different lags, Belgium, 1970-2000.* | *Cross-correlations between first birth hazards at age 30 and unemployment rate at different lags, Belgium, 1970-2000.* |
| --- | --- |
| ** | ** |
| Source: Author (2010). | |

*Table A.1 Models 0-12 using harmonized unemployment rate as macroeconomic indicator*

|  | |  | **Model 0** | | **Model 1** | | **Model 2** | | **Model 3** | | **Model 4** | |
| --- | --- | --- | --- | --- | --- | --- | --- | --- | --- | --- | --- | --- |
|  | |  | **b** | **sig.** | **b** | **sig.** | **b** | **sig.** | **b** | **sig.** | **b** | **sig.** |
| Baseline | | |  |  |  |  |  |  |  |  |  |  |
|  | time (linear) | | .8234 | *** | .5310 | *** | .6668 | *** | .8422 | *** | .9314 | *** |
|  | time (quadratic) | | -.0399 | *** | -.0285 | *** | -.0438 | *** | -.0407 | *** | -.0521 | *** |
|  | time (cubic) | | .0005 | *** | .0004 | *** | .0007 | *** | .0005 | *** | .0007 | *** |
| Education | | |  |  |  |  |  |  |  |  |  |  |
|  | LSE | |  |  | -.2308 | *** | -.5171 | *** |  |  |  |  |
|  | HSE | |  |  | -.4920 | *** | -1.8751 | *** |  |  |  |  |
|  | HEST | |  |  | -.5672 | *** | -4.2562 | *** |  |  |  |  |
|  | HELT | |  |  | -.7517 | *** | -7.1673 | *** |  |  |  |  |
| In education | | |  |  | -1.4492 | *** | -.9474 | *** |  |  |  |  |
| Years graduated | | |  |  |  |  |  |  |  |  |  |  |
|  | Linear | |  |  | .1739 | *** | .0842 | *** |  |  |  |  |
|  | Quadratic | |  |  | -.0110 | *** | -.0036 | *** |  |  |  |  |
| Education*Baseline | | |  |  |  |  |  |  |  |  |  |  |
|  | LSE*time (linear) | | |  |  |  | .0611 | *** |  |  |  |  |
|  | LSE*time (quadratic) | | |  |  |  | -.0019 | *** |  |  |  |  |
|  | HSE*time (linear) | | |  |  |  | .2118 | *** |  |  |  |  |
|  | HSE*time (quadratic) | | |  |  |  | -.0055 | *** |  |  |  |  |
|  | HEST*time (linear) | | |  |  |  | .4897 | *** |  |  |  |  |
|  | HEST*time (quadratic) | | |  |  |  | -.0124 | *** |  |  |  |  |
|  | HELT*time (linear) | | |  |  |  | .7522 | *** |  |  |  |  |
|  | HELT*time (quadratic) | | |  |  |  | -.0175 | *** |  |  |  |  |
| Unemployment Rate (UR, Lag 1) | | | |  |  |  |  |  | -.0742 | *** |  |  |
| Age*Unemployment Rate | | | |  |  |  |  |  |  |  |  |  |
|  | Age1519*URLag1 | | |  |  |  |  |  |  |  | -.1261 | *** |
|  | Age2024*URLag1 | | |  |  |  |  |  |  |  | -.1078 | *** |
|  | Age2529*URLag1 | | |  |  |  |  |  |  |  | -.0610 | *** |
|  | Age3034*URLag1 | | |  |  |  |  |  |  |  | -.0118 | *** |
|  | Age3539*URLag1 | | |  |  |  |  |  |  |  | .0294 | *** |
|  | Age4044*URLag1 | | |  |  |  |  |  |  |  | .0045 | ** |
|  | Age4549*URLag1 | | |  |  |  |  |  |  |  | -.2334 | *** |
| Constant | | | -6.9078 | *** | -5.1109 | *** | -5.0979 | *** | -6.5225 | *** | -6.4431 | *** |
|  | | |  |  |  |  |  |  |  |  |  |  |
| N | | | 47,354,001 | | 47,354,001 | | 47,354,001 | | 47,354,001 | | 47,354,001 | |
| Log Likelihood | | | -8695546.3 | | -8322569.1 | | -8262167.8 | | -8637812.4 | | -8611738.5 | |

*Table A.1 (con’t) Models 0-12 using harmonized unemployment rate as macroeconomic indicator*

|  | |  | **Model 5** | | | **Model 6** | | **Model 7** | | **Model 8** | |
| --- | --- | --- | --- | --- | --- | --- | --- | --- | --- | --- | --- |
|  | |  | **b** | | **sig.** | **b** | **sig.** | **b** | **sig.** | **b** | **sig.** |
| Baseline | | |  | |  |  |  |  |  |  |  |
|  | time (linear) | | .7905 | | *** | .6818 | *** | .8042 | *** | .8030 | *** |
|  | time (quadratic) | | -.0537 | | *** | -.0442 | *** | -.0538 | *** | -.0549 | *** |
|  | time (cubic) | | .0009 | | *** | .0007 | *** | .0010 | *** | .0010 | *** |
| Education | | |  | |  |  |  |  |  |  |  |
|  | LSE | | -.5249 | | *** | -.5196 | *** | -.4739 | *** | -.5344 | *** |
|  | HSE | | -1.9194 | | *** | -1.7834 | *** | -1.4799 | *** | -1.9424 | *** |
|  | HEST | | -4.4525 | | *** | -4.2028 | *** | -3.6264 | *** | -4.4882 | *** |
|  | HELT | | -7.4399 | | *** | -7.0168 | *** | -6.3590 | *** | -7.4815 | *** |
| In education | | | -.8833 | | *** | -.9231 | *** | -.8162 | *** | -.8785 | *** |
| Years graduated | | |  | |  |  |  |  |  |  |  |
|  | Linear | | .0778 | | *** | .0737 | *** | .0866 | *** | .0779 | *** |
|  | Quadratic | | -.0036 | | *** | -.0034 | *** | -.0040 | *** | -.0035 | *** |
| Education*Baseline | | |  | |  |  |  |  |  |  |  |
|  | LSE*time (linear) | | .0707 | | *** | .0640 | *** | .0610 | *** | .0726 | *** |
|  | LSE*time (quadratic) | | -.0023 | | *** | -.0019 | *** | -.0021 | *** | -.0023 | *** |
|  | HSE*time (linear) | | .2359 | | *** | .2183 | *** | .1886 | *** | .2405 | *** |
|  | HSE*time (quadratic) | | -.0065 | | *** | -.0056 | *** | -.0056 | *** | -.0067 | *** |
|  | HEST*time (linear) | | .5352 | | *** | .4975 | *** | .4549 | *** | .5418 | *** |
|  | HEST*time (quadratic) | | -.0142 | | *** | -.0126 | *** | -.0128 | *** | -.0144 | *** |
|  | HELT*time (linear) | | .8092 | | *** | .7607 | *** | .7201 | *** | .8168 | *** |
|  | HELT*time (quadratic) | | -.0197 | | *** | -.0177 | *** | -.0180 | *** | -.0200 | *** |
| Age*Unemployment Rate (Lag 1) | | | | |  |  |  |  |  |  |  |
|  | Age1519*URLag1 | | -.0324 | | *** |  |  |  |  | -.0314 | *** |
|  | Age2024*URLag1 | | -.0488 | | *** |  |  |  |  | -.0486 | *** |
|  | Age2529*URLag1 | | -.0391 | | *** |  |  |  |  | -.0363 | *** |
|  | Age3034*URLag1 | | -.0203 | | *** |  |  |  |  | -.0352 | *** |
|  | Age3539*URLag1 | | .0197 | | *** |  |  |  |  | -.0209 | *** |
|  | Age4044*URLag1 | | -.0007 | |  |  |  |  |  | -.0425 | *** |
|  | Age4549*URLag1 | | -.3287 | | *** |  |  |  |  | -.2530 | *** |
| Education*Unemployment Rate (Lag 1) | | | | |  |  |  |  |  |  |  |
|  | LE*URLag1 | |  | |  | -.0333 | *** |  |  |  |  |
|  | LSE*URLag1 | |  | |  | -.0310 | *** |  |  |  |  |
|  | HSE*URLag1 | |  | |  | -.0454 | *** |  |  |  |  |
|  | HEST*URLag1 | |  | |  | -.0436 | *** |  |  |  |  |
|  | Helt*URLag1 | |  | |  | -.0576 | *** |  |  |  |  |
| Education*Age*Unemployment Rate (Lag 1) | | | | | |  |  |  |  |  |  |
|  | LE*Age1519*URLag1 | | |  |  |  |  | .0038 | ** |  |  |
|  | LE*Age2024*URLag1 | | |  |  |  |  | -.0345 | *** |  |  |
|  | LE*Age2529*URLag1 | | |  |  |  |  | -.0580 | *** |  |  |
|  | LE*Age3034*URLag1 | | |  |  |  |  | -.0218 | *** |  |  |
|  | LE*Age3539*URLag1 | | |  |  |  |  | .0186 | *** |  |  |
|  | LE*Age4044*URLag1 | | |  |  |  |  | -.0677 | *** |  |  |
|  | LE*Age4549*URLag1 | | |  |  |  |  | -.6806 | *** |  |  |
|  | LSE*Age1519*URLag1 | | |  |  |  |  | -.0127 | *** |  |  |
|  | LSE*Age2024*URLag1 | | |  |  |  |  | -.0328 | *** |  |  |
|  | LSE*Age2529*URLag1 | | |  |  |  |  | -.0419 | *** |  |  |
|  | LSE*Age3034*URLag1 | | |  |  |  |  | -.0161 | *** |  |  |
|  | LSE*Age3539*URLag1 | | |  |  |  |  | .0180 | *** |  |  |
|  | LSE*Age4044*URLag1 | | |  |  |  |  | -.0549 | *** |  |  |
|  | LSE*Age4549*URLag1 | | |  |  |  |  | -.4652 | *** |  |  |
|  | HSE*Age1519*URLag1 | | |  |  |  |  | -.0742 | *** |  |  |
|  | HSE*Age2024*URLag1 | | |  |  |  |  | -.0534 | *** |  |  |
|  | HSE*Age2529*URLag1 | | |  |  |  |  | -.0424 | *** |  |  |
|  | HSE*Age3034*URLag1 | | |  |  |  |  | -.0167 | *** |  |  |
|  | HSE*Age3539*URLag1 | | |  |  |  |  | .0218 | *** |  |  |
|  |  | | |  |  |  |  |  |  |  |  |
|  | *continued on next page* | | |  |  |  |  |  |  |  |  |

*Table A.1 (con’t) Models 0-12 using harmonized unemployment rate as macroeconomic indicator*

|  | |  | **Model 5** | | | **Model 6** | | **Model 7** | | **Model 8** | |
| --- | --- | --- | --- | --- | --- | --- | --- | --- | --- | --- | --- |
|  | |  | **b** | | **sig.** | **b** | **sig.** | **b** | **sig.** | **b** | **sig.** |
|  | | |  | |  |  |  |  |  |  |  |
|  | HSE*Age4044*URLag1 | | |  |  |  |  | -.0270 | *** |  |  |
|  | HSE*Age4549*URLag1 | | |  |  |  |  | -.3378 | *** |  |  |
|  | HEST*Age1519*URLag1 | | |  |  |  |  | -.1065 | *** |  |  |
|  | HEST*Age2024*URLag1 | | |  |  |  |  | -.0840 | *** |  |  |
|  | HEST*Age2529*URLag1 | | |  |  |  |  | -.0381 | *** |  |  |
|  | HEST*Age3034*URLag1 | | |  |  |  |  | -.0166 | *** |  |  |
|  | HEST*Age3539*URLag1 | | |  |  |  |  | .0310 | *** |  |  |
|  | HEST*Age4044*URLag1 | | |  |  |  |  | .0400 | *** |  |  |
|  | HEST*Age4549*URLag1 | | |  |  |  |  | -.1403 | *** |  |  |
|  | HELT*Age1519*URLag1 | | |  |  |  |  | -.0620 | *** |  |  |
|  | HELT*Age2024*URLag1 | | |  |  |  |  | -.1369 | *** |  |  |
|  | HELT*Age2529*URLag1 | | |  |  |  |  | -.0622 | *** |  |  |
|  | HELT*Age3034*URLag1 | | |  |  |  |  | -.0416 | *** |  |  |
|  | HELT*Age3539*URLag1 | | |  |  |  |  | -.0097 | *** |  |  |
|  | HELT*Age4044*URLag1 | | |  |  |  |  | .0059 |  |  |  |
|  | HELT*Age4549*URLag1 | | |  |  |  |  | -.1459 | *** |  |  |
| Age*Unemployment Rate (Lag 10) | | | | |  |  |  |  |  |  |  |
|  | Age3034*URLag10 | | |  |  |  |  |  |  | .0255 | *** |
|  | Age3539*URLag10 | | |  |  |  |  |  |  | .0608 | *** |
|  | Age4044*URLag10 | | |  |  |  |  |  |  | .0697 | *** |
|  | Age4549*URLag10 | | |  |  |  |  |  |  | -.0442 | *** |
| Constant | | | -5.2933 | | *** | -4.9823 | *** | -5.4954 | *** | -5.3306 | *** |
|  | | |  | |  |  |  |  |  |  |  |
| N | | | 47,354,001 | | | 47,354,001 | | 47,354,001 | | 47,354,001 | |
| Log Likelihood | | | -8241891.1 | | | -8247378.9 | | -8235180.0 | | -8239464.8 | |

*Table A.1 (con’t) Models 0-12 using harmonized unemployment rate as macroeconomic indicator*

|  | |  | **Model 9** | | | **Model 10** | | **Model 11** | | **Model 12** | |
| --- | --- | --- | --- | --- | --- | --- | --- | --- | --- | --- | --- |
|  | |  | **b** | | **sig.** | **b** | **sig.** | **b** | **sig.** | **b** | **sig.** |
| Baseline | | |  | |  |  |  |  |  |  |  |
|  | time (linear) | | .8245 | | *** | .8147 | *** | .7502 | *** | .7986 | *** |
|  | time (quadratic) | | -.0558 | | *** | -.0555 | *** | -.0509 | *** | -.0550 | *** |
|  | time (cubic) | | .0010 | | *** | .0010 | *** | .0009 | *** | .0010 | *** |
| Education | | |  | |  |  |  |  |  |  |  |
|  | LSE | | -.4809 | | *** | -.5688 | *** | -.6425 | *** | -.5838 | *** |
|  | HSE | | -1.5070 | | *** | -2.0058 | *** | -2.1214 | *** | -1.9613 | *** |
|  | HEST | | -3.6990 | | *** | -4.5842 | *** | -4.6491 | *** | -4.5153 | *** |
|  | HELT | | -6.4695 | | *** | -7.5438 | *** | -7.5046 | *** | -7.9043 | *** |
| In education | | | -.8080 | | *** | -.8537 | *** | -.8735 | *** | -.7674 | *** |
| Years graduated | | |  | |  |  |  |  |  |  |  |
|  | Linear | | .0870 | | *** | .0685 | *** | .0579 | *** | .0747 | *** |
|  | Quadratic | | -.0039 | | *** | -.0033 | *** | -.0026 | *** | -.0033 | *** |
| Education*Baseline | | |  | |  |  |  |  |  |  |  |
|  | LSE*time (linear) | | .0603 | | *** | .0742 | *** | .0892 | *** | .0741 | *** |
|  | LSE*time (quadratic) | | -.0019 | | *** | -.0023 | *** | -.0029 | *** | -.0022 | *** |
|  | HSE*time (linear) | | .1903 | | *** | .2482 | *** | .2721 | *** | .2405 | *** |
|  | HSE*time (quadratic) | | -.0054 | | *** | -.0068 | *** | -.0078 | *** | -.0063 | *** |
|  | HEST*time (linear) | | .4625 | | *** | .5523 | *** | .5675 | *** | .5521 | *** |
|  | HEST*time (quadratic) | | -.0128 | | *** | -.0147 | *** | -.0153 | *** | -.0143 | *** |
|  | HELT*time (linear) | | .7296 | | *** | .8232 | *** | .8250 | *** | .8723 | *** |
|  | HELT*time (quadratic) | | -.0179 | | *** | -.0201 | *** | -.0204 | *** | -.0208 | *** |
| Age*Unemployment Rate (Lag 1) | | | | |  |  |  |  |  |  |  |
|  | Age1519*URLag1 | |  | |  | -.0357 | *** |  |  |  |  |
|  | Age2024*URLag1 | |  | |  | -.0519 | *** |  |  |  |  |
|  | Age2529*URLag1 | |  | |  | -.0359 | *** |  |  |  |  |
|  | Age3034*URLag1 | |  | |  | -.0532 | *** |  |  |  |  |
|  | Age3539*URLag1 | |  | |  | -.0352 | *** |  |  |  |  |
|  | Age4044*URLag1 | |  | |  | -.0528 | *** |  |  |  |  |
|  | Age4549*URLag1 | |  | |  | -.2603 | *** |  |  |  |  |
| Education*Age*Unemployment Rate (Lag 1) | | | | | |  |  |  |  |  |  |
|  | LE*Age1519*URLag1 | | | .0038 | ** |  |  |  |  |  |  |
|  | LE*Age2024*URLag1 | | | -.0349 | *** |  |  |  |  |  |  |
|  | LE*Age2529*URLag1 | | | -.0520 | *** |  |  |  |  |  |  |
|  | LE*Age3034*URLag1 | | | -.0368 | *** |  |  |  |  |  |  |
|  | LE*Age3539*URLag1 | | | -.0267 | *** |  |  |  |  |  |  |
|  | LE*Age4044*URLag1 | | | -.0828 | *** |  |  |  |  |  |  |
|  | LE*Age4549*URLag1 | | | -.0119 | *** |  |  |  |  |  |  |
|  | LSE*Age1519*URLag1 | | | -.0119 | *** |  |  |  |  |  |  |
|  | LSE*Age2024*URLag1 | | | -.0331 | *** |  |  |  |  |  |  |
|  | LSE*Age2529*URLag1 | | | -.0392 | *** |  |  |  |  |  |  |
|  | LSE*Age3034*URLag1 | | | -.0290 | *** |  |  |  |  |  |  |
|  | LSE*Age3539*URLag1 | | | -.0177 | *** |  |  |  |  |  |  |
|  | LSE*Age4044*URLag1 | | | -.0799 | *** |  |  |  |  |  |  |
|  | LSE*Age4549*URLag1 | | | -.3625 | *** |  |  |  |  |  |  |
|  | HSE*Age1519*URLag1 | | | -.0727 | *** |  |  |  |  |  |  |
|  | HSE*Age2024*URLag1 | | | -.0534 | *** |  |  |  |  |  |  |
|  | HSE*Age2529*URLag1 | | | -.0406 | *** |  |  |  |  |  |  |
|  | HSE*Age3034*URLag1 | | | -.0292 | *** |  |  |  |  |  |  |
|  | HSE*Age3539*URLag1 | | | -.0164 | *** |  |  |  |  |  |  |
|  | HSE*Age4044*URLag1 | | | -.0650 | *** |  |  |  |  |  |  |
|  | HSE*Age4549*URLag1 | | | -.2572 | *** |  |  |  |  |  |  |
|  | HEST*Age1519*URLag1 | | | -.1023 | *** |  |  |  |  |  |  |
|  | HEST*Age2024*URLag1 | | | -.0829 | *** |  |  |  |  |  |  |
|  | HEST*Age2529*URLag1 | | | -.0356 | *** |  |  |  |  |  |  |
|  | HEST*Age3034*URLag1 | | | -.0333 | *** |  |  |  |  |  |  |
|  |  | | |  |  |  |  |  |  |  |  |
|  | *continued on next page* | | |  |  |  |  |  |  |  |  |

*Table A.1 (con’t) Models 0-12 using harmonized unemployment rate as macroeconomic indicator*

|  | |  | **Model 9** | | | **Model 10** | | **Model 11** | | **Model 12** | |
| --- | --- | --- | --- | --- | --- | --- | --- | --- | --- | --- | --- |
|  | |  | **b** | | **sig.** | **b** | **sig.** | **b** | **sig.** | **b** | **sig.** |
|  | HEST*Age3539*URLag1 | | | -.0054 | * |  |  |  |  |  |  |
|  | HEST*Age4044*URLag1 | | | -.0062 |  |  |  |  |  |  |  |
|  | HEST*Age4549*URLag1 | | | -.1163 | *** |  |  |  |  |  |  |
|  | HELT*Age1519*URLag1 | | | -.0546 | *** |  |  |  |  |  |  |
|  | HELT*Age2024*URLag1 | | | -.1333 | *** |  |  |  |  |  |  |
|  | HELT*Age2529*URLag1 | | | -.0575 | *** |  |  |  |  |  |  |
|  | HELT*Age3034*URLag1 | | | -.0551 | *** |  |  |  |  |  |  |
|  | HELT*Age3539*URLag1 | | | -.0457 | *** |  |  |  |  |  |  |
|  | HELT*Age4044*URLag1 | | | -.0318 | *** |  |  |  |  |  |  |
|  | HELT*Age4549*URLag1 | | | -.1948 | *** |  |  |  |  |  |  |
| Age*Unemployment Rate (Lag 10) | | | | |  |  |  |  |  |  |  |
|  | Age3034*URLag10 | | |  |  | .0506 | *** |  |  |  |  |
|  | Age3539*URLag10 | | |  |  | .0872 | *** |  |  |  |  |
|  | Age4044*URLag10 | | |  |  | .0979 | *** |  |  |  |  |
|  | Age4549*URLag10 | | |  |  | -.0135 |  |  |  |  |  |
| Age*Unemployment Rate (Lag 10) | | | | |  |  |  |  |  |  |  |
|  | LE*Age3034*URLag10 | | | .0367 | *** |  |  |  |  |  |  |
|  | LE*Age3539*URLag10 | | | .0852 | *** |  |  |  |  |  |  |
|  | LE*Age4044*URLag10 | | | .0672 | *** |  |  |  |  |  |  |
|  | LE*Age4549*URLag10 | | | -.1462 | *** |  |  |  |  |  |  |
|  | LSE*Age3034*URLag10 | | | .0261 | *** |  |  |  |  |  |  |
|  | LSE*Age3539*URLag10 | | | .0588 | *** |  |  |  |  |  |  |
|  | LSE*Age4044*URLag10 | | | .0499 | *** |  |  |  |  |  |  |
|  | LSE*Age4549*URLag10 | | | -.0760 | ** |  |  |  |  |  |  |
|  | HSE*Age3034*URLag10 | | | .0211 | *** |  |  |  |  |  |  |
|  | HSE*Age3539*URLag10 | | | .0539 | *** |  |  |  |  |  |  |
|  | HSE*Age4044*URLag10 | | | .0558 | *** |  |  |  |  |  |  |
|  | HSE*Age4549*URLag10 | | | -.0658 | * |  |  |  |  |  |  |
|  | HEST*Age3034*URLag10 | | | .0252 | *** |  |  |  |  |  |  |
|  | HEST*Age3539*URLag10 | | | .0527 | *** |  |  |  |  |  |  |
|  | HEST*Age4044*URLag10 | | | .0682 | *** |  |  |  |  |  |  |
|  | HEST*Age4549*URLag10 | | | -.0018 |  |  |  |  |  |  |  |
|  | HELT*Age3034*URLag10 | | | .0229 | *** |  |  |  |  |  |  |
|  | HELT*Age3539*URLag10 | | | .0518 | *** |  |  |  |  |  |  |
|  | HELT*Age4044*URLag10 | | | .0576 | *** |  |  |  |  |  |  |
|  | HELT*Age4549*URLag10 | | | .0687 |  |  |  |  |  |  |  |
| Period*Unemployment Rate (Lag 1) | | | | |  |  |  |  |  |  |  |
|  | 19601973*URLag1 | | |  |  | .0213 | *** |  |  |  |  |
|  | 19741991*URLag1 | | |  |  | .0188 | *** |  |  |  |  |
|  | 19922010*URLag1 | | |  |  | .0000 |  |  |  |  |  |
| Period*Unemployment Rate (Lag 10) | | | | |  |  |  |  |  |  |  |
|  | 19601973*URLag10 | | |  |  | -.0246 | *** |  |  |  |  |
|  | 19741991*URLag10 | | |  |  | -.0325 | *** |  |  |  |  |
|  | 19922010*URLag10 | | |  |  | -.0171 | *** |  |  |  |  |
| Period*Age*Unemployment Rate (Lag 1) | | | | | |  |  |  |  |  |  |
|  | 19601973*Age1519*URLag1 | | | |  |  |  | -.1181 | *** |  |  |
|  | 19601973*Age2024*URLag1 | | | |  |  |  | -.0214 | *** |  |  |
|  | 19601973*Age2529*URLag1 | | | |  |  |  | -.0587 | *** |  |  |
|  | 19601973*Age3034*URLag1 | | | |  |  |  | -.0912 | *** |  |  |
|  | 19601973*Age3539*URLag1 | | | |  |  |  | -.0260 | ** |  |  |
|  | 19601973*Age4044*URLag1 | | | |  |  |  | -.1509 | *** |  |  |
|  | 19601973*Age4549*URLag1 | | | |  |  |  | -1.3462 | *** |  |  |
|  | 19741991*Age1519*URLag1 | | | |  |  |  | -.0297 | *** |  |  |
|  | 19741991*Age2024*URLag1 | | | |  |  |  | -.0353 | *** |  |  |
|  | 19741991*Age2529*URLag1 | | | |  |  |  | -.0291 | *** |  |  |
|  | 19741991*Age3034*URLag1 | | | |  |  |  | -.0579 | *** |  |  |
|  |  | | | |  |  |  |  |  |  |  |
|  | *continued on next page* | | | |  |  |  |  |  |  |  |

*Table A.1 (con’t) Models 0-12 using harmonized unemployment rate as macroeconomic indicator*

|  | |  | **Model 9** | | **Model 10** | | **Model 11** | | **Model 12** | |
| --- | --- | --- | --- | --- | --- | --- | --- | --- | --- | --- |
|  | |  | **b** | **sig.** | **b** | **sig.** | **b** | **sig.** | **b** | **sig.** |
|  | 19741991*Age3539*URLag1 | | |  |  |  | -.0571 | *** |  |  |
|  | 19741991*Age4044*URLag1 | | |  |  |  | -.1189 | *** |  |  |
|  | 19741991*Age4549*URLag1 | | |  |  |  | -.5144 | *** |  |  |
|  | 19922010*Age1519*URLag1 | | |  |  |  | -.0403 | *** |  |  |
|  | 19922010*Age2024*URLag1 | | |  |  |  | -.0754 | *** |  |  |
|  | 19922010*Age2529*URLag1 | | |  |  |  | -.0513 | *** |  |  |
|  | 19922010*Age3034*URLag1 | | |  |  |  | -.0037 | ** |  |  |
|  | 19922010*Age3539*URLag1 | | |  |  |  | .0390 | *** |  |  |
|  | 19922010*Age4044*URLag1 | | |  |  |  | .0328 | *** |  |  |
|  | 19922010*Age4549*URLag1 | | |  |  |  | -.1474 | *** |  |  |
| Period*Age*Unemployment Rate (Lag 10) | | | |  |  |  |  |  |  |  |
| 19601973*Age3034*URLag10 | | |  |  |  |  | .0076 | *** |  |  |
| 19601973*Age3539*URLag10 | | |  |  |  |  | .0170 | *** |  |  |
| 19601973*Age4044*URLag10 | | |  |  |  |  | .0170 | * |  |  |
| 19601973*Age4549*URLag10 | | |  |  |  |  | .0193 |  |  |  |
| 19741991*Age3034*URLag10 | | |  |  |  |  | .0178 | *** |  |  |
| 19741991*Age3539*URLag10 | | |  |  |  |  | .0358 | *** |  |  |
| 19741991*Age4044*URLag10 | | |  |  |  |  | .0477 | *** |  |  |
| 19741991*Age4549*URLag10 | | |  |  |  |  | .0075 |  |  |  |
| 19922010*Age3034*URLag10 | | |  |  |  |  | -.0109 | *** |  |  |
| 19922010*Age3539*URLag10 | | |  |  |  |  | -.0100 | *** |  |  |
| 19922010*Age4044*URLag10 | | |  |  |  |  | -.0208 | ** |  |  |
| 19922010*Age4549*URLag10 | | |  |  |  |  | -.1140 | *** |  |  |
| Period*Age*Education*Unemployment Rate (Lag 1) | | | | | | | | | | |
| LE*Age1519*19601973*URLag1 | | | |  |  |  |  |  | -.1023 | *** |
| LE*Age1519*19741991*URLag1 | | | |  |  |  |  |  | -.0127 | *** |
| LE*Age1519*19922010*URLag1 | | | |  |  |  |  |  | .0111 | *** |
| LE*Age2024*19601973*URLag1 | | | |  |  |  |  |  | -.0486 | *** |
| LE*Age2024*19741991*URLag1 | | | |  |  |  |  |  | -.0432 | *** |
| LE*Age2024*19922010*URLag1 | | | |  |  |  |  |  | -.0393 | *** |
| LE*Age2529*19601973*URLag1 | | | |  |  |  |  |  | -.0566 | *** |
| LE*Age2529*19741991*URLag1 | | | |  |  |  |  |  | -.0521 | *** |
| LE*Age2529*19922010*URLag1 | | | |  |  |  |  |  | -.0487 | *** |
| LE*Age3034*19601973*URLag1 | | | |  |  |  |  |  | .0059 |  |
| LE*Age3034*19741991*URLag1 | | | |  |  |  |  |  | -.0347 | *** |
| LE*Age3034*19922010*URLag1 | | | |  |  |  |  |  | .0402 | *** |
| LE*Age3539*19601973*URLag1 | | | |  |  |  |  |  | .1152 | *** |
| LE*Age3539*19741991*URLag1 | | | |  |  |  |  |  | -.0336 | *** |
| LE*Age3539*19922010*URLag1 | | | |  |  |  |  |  | .0881 | *** |
| LE*Age4044*19601973*URLag1 | | | |  |  |  |  |  | -.0858 | ** |
| LE*Age4044*19741991*URLag1 | | | |  |  |  |  |  | -.1135 | *** |
| LE*Age4044*19922010*URLag1 | | | |  |  |  |  |  | .0431 |  |
| LE*Age4549*19601973*URLag1 | | | |  |  |  |  |  | -1.3094 | *** |
| LE*Age4549*19741991*URLag1 | | | |  |  |  |  |  | -.5373 | *** |
| LE*Age4549*19922010*URLag1 | | | |  |  |  |  |  | -.2286 | ** |
| LSE*Age1519*19601973*URLag1 | | | |  |  |  |  |  | -.1250 | *** |
| LSE*Age1519*19741991*URLag1 | | | |  |  |  |  |  | -.0218 | *** |
| LSE*Age1519*19922010*URLag1 | | | |  |  |  |  |  | -.0236 | *** |
| LSE*Age2024*19601973*URLag1 | | | |  |  |  |  |  | -.0430 | *** |
| LSE*Age2024*19741991*URLag1 | | | |  |  |  |  |  | -.0350 | *** |
| LSE*Age2024*19922010*URLag1 | | | |  |  |  |  |  | -.0472 | *** |
| LSE*Age2529*19601973*URLag1 | | | |  |  |  |  |  | -.0508 | *** |
| LSE*Age2529*19741991*URLag1 | | | |  |  |  |  |  | -.0346 | *** |
| LSE*Age2529*19922010*URLag1 | | | |  |  |  |  |  | -.0499 | *** |
| LSE*Age3034*19601973*URLag1 | | | |  |  |  |  |  | .0003 |  |
| LSE*Age3034*19741991*URLag1 | | | |  |  |  |  |  | -.0338 | *** |
|  | | | |  |  |  |  |  |  |  |
| *continued on next page* | | | |  |  |  |  |  |  |  |

*Table A.1 (con’t) Models 0-12 using harmonized unemployment rate as macroeconomic indicator*

|  |  | **Model 9** | | **Model 10** | | **Model 11** | | **Model 12** | |
| --- | --- | --- | --- | --- | --- | --- | --- | --- | --- |
|  |  | **b** | **sig.** | **b** | **sig.** | **b** | **sig.** | **b** | **sig.** |
| LSE*Age3034*19922010*URLag1 | | |  |  |  |  |  | .0113 | * |
| LSE*Age3539*19601973*URLag1 | | |  |  |  |  |  | .0750 | *** |
| LSE*Age3539*19741991*URLag1 | | |  |  |  |  |  | -.0196 | *** |
| LSE*Age3539*19922010*URLag1 | | |  |  |  |  |  | .0440 | *** |
| LSE*Age4044*19601973*URLag1 | | |  |  |  |  |  | -.0263 |  |
| LSE*Age4044*19741991*URLag1 | | |  |  |  |  |  | -.1140 | *** |
| LSE*Age4044*19922010*URLag1 | | |  |  |  |  |  | .01439 |  |
| LSE*Age4549*19601973*URLag1 | | |  |  |  |  |  | -1.3307 | *** |
| LSE*Age4549*19741991*URLag1 | | |  |  |  |  |  | -.5492 | *** |
| LSE*Age4549*19922010*URLag1 | | |  |  |  |  |  | -.2974 | *** |
| HSE*Age1519*19601973*URLag1 | | |  |  |  |  |  | -.0489 | *** |
| HSE*Age1519*19741991*URLag1 | | |  |  |  |  |  | -.0497 | *** |
| HSE*Age1519*19922010*URLag1 | | |  |  |  |  |  | -.0801 | *** |
| HSE*Age2024*19601973*URLag1 | | |  |  |  |  |  | .0014 |  |
| HSE*Age2024*19741991*URLag1 | | |  |  |  |  |  | -.0323 | *** |
| HSE*Age2024*19922010*URLag1 | | |  |  |  |  |  | -.0711 | *** |
| HSE*Age2529*19601973*URLag1 | | |  |  |  |  |  | -.0740 | *** |
| HSE*Age2529*19741991*URLag1 | | |  |  |  |  |  | -.0308 | *** |
| HSE*Age2529*19922010*URLag1 | | |  |  |  |  |  | -.0526 | *** |
| HSE*Age3034*19601973*URLag1 | | |  |  |  |  |  | -.0736 | *** |
| HSE*Age3034*19741991*URLag1 | | |  |  |  |  |  | -.0520 | *** |
| HSE*Age3034*19922010*URLag1 | | |  |  |  |  |  | -.0012 |  |
| HSE*Age3539*19601973*URLag1 | | |  |  |  |  |  | -.0290 |  |
| HSE*Age3539*19741991*URLag1 | | |  |  |  |  |  | -.0501 | *** |
| HSE*Age3539*19922010*URLag1 | | |  |  |  |  |  | .0370 | *** |
| HSE*Age4044*19601973*URLag1 | | |  |  |  |  |  | -.1326 | ** |
| HSE*Age4044*19741991*URLag1 | | |  |  |  |  |  | -.1178 | *** |
| HSE*Age4044*19922010*URLag1 | | |  |  |  |  |  | -.0256 |  |
| HSE*Age4549*19601973*URLag1 | | |  |  |  |  |  | -1.3106 | *** |
| HSE*Age4549*19741991*URLag1 | | |  |  |  |  |  | -.3607 | *** |
| HSE*Age4549*19922010*URLag1 | | |  |  |  |  |  | -.2827 | *** |
| HEST*Age1519*19601973*URLag1 | | |  |  |  |  |  | .0225 |  |
| HEST*Age1519*19741991*URLag1 | | |  |  |  |  |  | -.0568 | *** |
| HEST*Age1519*19922010*URLag1 | | |  |  |  |  |  | -.0930 | *** |
| HEST*Age2024*19601973*URLag1 | | |  |  |  |  |  | .0632 | *** |
| HEST*Age2024*19741991*URLag1 | | |  |  |  |  |  | -.0428 | *** |
| HEST*Age2024*19922010*URLag1 | | |  |  |  |  |  | -.1160 | *** |
| HEST*Age2529*19601973*URLag1 | | |  |  |  |  |  | -.0901 | *** |
| HEST*Age2529*19741991*URLag1 | | |  |  |  |  |  | -.0285 | *** |
| HEST*Age2529*19922010*URLag1 | | |  |  |  |  |  | -.0552 | *** |
| HEST*Age3034*19601973*URLag1 | | |  |  |  |  |  | -.2372 | *** |
| HEST*Age3034*19741991*URLag1 | | |  |  |  |  |  | -.0810 | *** |
| HEST*Age3034*19922010*URLag1 | | |  |  |  |  |  | -.0153 | *** |
| HEST*Age3539*19601973*URLag1 | | |  |  |  |  |  | -.1999 | *** |
| HEST*Age3539*19741991*URLag1 | | |  |  |  |  |  | -.0849 | *** |
| HEST*Age3539*19922010*URLag1 | | |  |  |  |  |  | .0309 | *** |
| HEST*Age4044*19601973*URLag1 | | |  |  |  |  |  | -.2956 | *** |
| HEST*Age4044*19741991*URLag1 | | |  |  |  |  |  | -.1059 | *** |
| HEST*Age4044*19922010*URLag1 | | |  |  |  |  |  | .0249 | * |
| HEST*Age4549*19601973*URLag1 | | |  |  |  |  |  | -.9478 | *** |
| HEST*Age4549*19741991*URLag1 | | |  |  |  |  |  | -.3069 | *** |
| HEST*Age4549*19922010*URLag1 | | |  |  |  |  |  | -.0406 |  |
| HELT*Age1519*19601973*URLag1 | | |  |  |  |  |  | .6421 | *** |
| HELT*Age1519*19741991*URLag1 | | |  |  |  |  |  | .0558 | *** |
| HELT*Age1519*19922010*URLag1 | | |  |  |  |  |  | -.0701 | *** |
| HELT*Age2024*19601973*URLag1 | | |  |  |  |  |  | .3647 | *** |
|  | | |  |  |  |  |  |  |  |
| *continued on next page* | | |  |  |  |  |  |  |  |

*Table A.1 (con’t) Models 0-12 using harmonized unemployment rate as macroeconomic indicator*

|  |  | **Model 9** | | **Model 10** | | **Model 11** | | **Model 12** | |
| --- | --- | --- | --- | --- | --- | --- | --- | --- | --- |
|  |  | **b** | **sig.** | **b** | **sig.** | **b** | **sig.** | **b** | **sig.** |
| HELT*Age2024*19741991*URLag1 | | |  |  |  |  |  | -.0230 | *** |
| HELT*Age2024*19922010*URLag1 | | |  |  |  |  |  | -.1882 | *** |
| HELT*Age2529*19601973*URLag1 | | |  |  |  |  |  | .0608 | *** |
| HELT*Age2529*19741991*URLag1 | | |  |  |  |  |  | -.0199 | *** |
| HELT*Age2529*19922010*URLag1 | | |  |  |  |  |  | -.0659 | *** |
| HELT*Age3034*19601973*URLag1 | | |  |  |  |  |  | -.3250 | *** |
| HELT*Age3034*19741991*URLag1 | | |  |  |  |  |  | -.0900 | *** |
| HELT*Age3034*19922010*URLag1 | | |  |  |  |  |  | -.0178 | *** |
| HELT*Age3539*19601973*URLag1 | | |  |  |  |  |  | -.4641 | *** |
| HELT*Age3539*19741991*URLag1 | | |  |  |  |  |  | -.1118 | *** |
| HELT*Age3539*19922010*URLag1 | | |  |  |  |  |  | .0091 |  |
| HELT*Age4044*19601973*URLag1 | | |  |  |  |  |  | -.2710 |  |
| HELT*Age4044*19741991*URLag1 | | |  |  |  |  |  | -.1229 | *** |
| HELT*Age4044*19922010*URLag1 | | |  |  |  |  |  | .0250 |  |
| HELT*Age4549*19601973*URLag1 | | |  |  |  |  |  | -1.8216 | ** |
| HELT*Age4549*19741991*URLag1 | | |  |  |  |  |  | -.3218 | *** |
| HELT*Age4549*19922010*URLag1 | | |  |  |  |  |  | -.1437 | * |
| LE*Age3034*19601973*URLag10 | | |  |  |  |  |  | .0230 | *** |
| LE*Age3034*19741991*URLag10 | | |  |  |  |  |  | .0181 | *** |
| LE*Age3034*19922010*URLag10 | | |  |  |  |  |  | -.0242 | ** |
| LE*Age3539*19601973*URLag10 | | |  |  |  |  |  | .0324 | *** |
| LE*Age3539*19741991*URLag10 | | |  |  |  |  |  | .0518 | *** |
| LE*Age3539*19922010*URLag10 | | |  |  |  |  |  | -.0154 |  |
| LE*Age4044*19601973*URLag10 | | |  |  |  |  |  | .0416 | *** |
| LE*Age4044*19741991*URLag10 | | |  |  |  |  |  | .0403 | * |
| LE*Age4044*19922010*URLag10 | | |  |  |  |  |  | -.0481 | * |
| LE*Age4549*19601973*URLag10 | | |  |  |  |  |  | .0249 |  |
| LE*Age4549*19741991*URLag10 | | |  |  |  |  |  | -.1279 |  |
| LE*Age4549*19922010*URLag10 | | |  |  |  |  |  | -.1544 |  |
| LSE*Age3034*19601973*URLag10 | | |  |  |  |  |  | .0130 | *** |
| LSE*Age3034*19741991*URLag10 | | |  |  |  |  |  | .0241 | *** |
| LSE*Age3034*19922010*URLag10 | | |  |  |  |  |  | -.0084 | * |
| LSE*Age3539*19601973*URLag10 | | |  |  |  |  |  | .0283 | *** |
| LSE*Age3539*19741991*URLag10 | | |  |  |  |  |  | .0327 | *** |
| LSE*Age3539*19922010*URLag10 | | |  |  |  |  |  | .0002 |  |
| LSE*Age4044*19601973*URLag10 | | |  |  |  |  |  | .0128 |  |
| LSE*Age4044*19741991*URLag10 | | |  |  |  |  |  | .0472 | ** |
| LSE*Age4044*19922010*URLag10 | | |  |  |  |  |  | -.0456 | * |
| LSE*Age4549*19601973*URLag10 | | |  |  |  |  |  | .0440 |  |
| LSE*Age4549*19741991*URLag10 | | |  |  |  |  |  | .0053 |  |
| LSE*Age4549*19922010*URLag10 | | |  |  |  |  |  | -.0814 |  |
| HSE*Age3034*19601973*URLag10 | | |  |  |  |  |  | -.0124 | ** |
| HSE*Age3034*19741991*URLag10 | | |  |  |  |  |  | .0177 | *** |
| HSE*Age3034*19922010*URLag10 | | |  |  |  |  |  | -.0110 | *** |
| HSE*Age3539*19601973*URLag10 | | |  |  |  |  |  | .0115 |  |
| HSE*Age3539*19741991*URLag10 | | |  |  |  |  |  | .0301 | *** |
| HSE*Age3539*19922010*URLag10 | | |  |  |  |  |  | -.0108 | * |
| HSE*Age4044*19601973*URLag10 | | |  |  |  |  |  | .0131 |  |
| HSE*Age4044*19741991*URLag10 | | |  |  |  |  |  | .0236 |  |
| HSE*Age4044*19922010*URLag10 | | |  |  |  |  |  | -.0068 |  |
| HSE*Age4549*19601973*URLag10 | | |  |  |  |  |  | -.0134 |  |
| HSE*Age4549*19741991*URLag10 | | |  |  |  |  |  | -.1645 |  |
| HSE*Age4549*19922010*URLag10 | | |  |  |  |  |  | -.0526 |  |
| HEST*Age3034*19601973*URLag10 | | |  |  |  |  |  | -.0435 | *** |
| HEST*Age3034*19741991*URLag10 | | |  |  |  |  |  | .0214 | *** |
| HEST*Age3034*19922010*URLag10 | | |  |  |  |  |  | -.0110 | *** |
|  | | |  |  |  |  |  |  |  |
| *continued on next page* | | |  |  |  |  |  |  |  |

*Table A.1 (con’t) Models 0-12 using harmonized unemployment rate as macroeconomic indicator*

|  |  | **Model 9** | | **Model 10** | | **Model 11** | | **Model 12** | |
| --- | --- | --- | --- | --- | --- | --- | --- | --- | --- |
|  |  | **b** | **sig.** | **b** | **sig.** | **b** | **sig.** | **b** | **sig.** |
| HEST*Age3539*19601973*URLag10 | | |  |  |  |  |  | -.0521 | *** |
| HEST*Age3539*19741991*URLag10 | | |  |  |  |  |  | .0423 | *** |
| HEST*Age3539*19922010*URLag10 | | |  |  |  |  |  | -.0147 | ** |
| HEST*Age4044*19601973*URLag10 | | |  |  |  |  |  | -.0105 |  |
| HEST*Age4044*19741991*URLag10 | | |  |  |  |  |  | .0397 | * |
| HEST*Age4044*19922010*URLag10 | | |  |  |  |  |  | -.0083 |  |
| HEST*Age4549*19601973*URLag10 | | |  |  |  |  |  | -.0311 |  |
| HEST*Age4549*19741991*URLag10 | | |  |  |  |  |  | .0492 |  |
| HEST*Age4549*19922010*URLag10 | | |  |  |  |  |  | -.1264 | * |
| HELT*Age3034*19601973*URLag10 | | |  |  |  |  |  | -.0486 | *** |
| HELT*Age3034*19741991*URLag10 | | |  |  |  |  |  | .0137 | *** |
| HELT*Age3034*19922010*URLag10 | | |  |  |  |  |  | -.0211 | *** |
| HELT*Age3539*19601973*URLag10 | | |  |  |  |  |  | -.0194 |  |
| HELT*Age3539*19741991*URLag10 | | |  |  |  |  |  | .0361 | *** |
| HELT*Age3539*19922010*URLag10 | | |  |  |  |  |  | -.0200 | *** |
| HELT*Age4044*19601973*URLag10 | | |  |  |  |  |  | -.1567 | * |
| HELT*Age4044*19741991*URLag10 | | |  |  |  |  |  | .0294 |  |
| HELT*Age4044*19922010*URLag10 | | |  |  |  |  |  | -.0196 |  |
| HELT*Age4549*19601973*URLag10 | | |  |  |  |  |  | .3412 |  |
| HELT*Age4549*19741991*URLag10 | | |  |  |  |  |  | .0354 |  |
| HELT*Age4549*19922010*URLag10 | | |  |  |  |  |  | .0047 |  |
| Constant | | -5.5480 | *** | -5.2640 | *** | -5.0513 | *** | -5.2804 | *** |
|  | |  |  |  |  |  |  |  |  |
| N | | 47,354,001 | | 47,354,001 | | 47,354,001 | | 47,354,001 | |
| Log Likelihood | | -8232784.9 | | -8230938.7 | | -8224307.1 | | -8208429.7 | |

*Table A.2 Models 0-12 using consumer price index (CPI) as macroeconomic indicator*

|  | |  | **Model 0** | | **Model 1** | | **Model 2** | | **Model 3** | | **Model 4** | |
| --- | --- | --- | --- | --- | --- | --- | --- | --- | --- | --- | --- | --- |
|  | |  | **b** | **sig.** | **b** | **sig.** | **b** | **sig.** | **b** | **sig.** | **b** | **sig.** |
| Baseline | | |  |  |  |  |  |  |  |  |  |  |
|  | time (linear) | | - |  | - |  | - |  | .8436 | *** | .9944 | *** |
|  | time (quadratic) | | - |  | - |  | - |  | -.0405 | *** | -.0575 | *** |
|  | time (cubic) | | - |  | - |  | - |  | .0005 | *** | .0008 | *** |
| Education | | |  |  |  |  |  |  |  |  |  |  |
|  | LSE | |  |  | - |  | - |  |  |  |  |  |
|  | HSE | |  |  | - |  | - |  |  |  |  |  |
|  | HEST | |  |  | - |  | - |  |  |  |  |  |
|  | HELT | |  |  | - |  | - |  |  |  |  |  |
| In education | | |  |  | - |  | - |  |  |  |  |  |
| Years graduated | | |  |  |  |  |  |  |  |  |  |  |
|  | Linear | |  |  | - |  | - |  |  |  |  |  |
|  | Quadratic | |  |  | - |  | - |  |  |  |  |  |
| Education*Baseline | | |  |  |  |  |  |  |  |  |  |  |
|  | LSE*time (linear) | | |  |  |  | - |  |  |  |  |  |
|  | LSE*time (quadratic) | | |  |  |  | - |  |  |  |  |  |
|  | HSE*time (linear) | | |  |  |  | - |  |  |  |  |  |
|  | HSE*time (quadratic) | | |  |  |  | - |  |  |  |  |  |
|  | HEST*time (linear) | | |  |  |  | - |  |  |  |  |  |
|  | HEST*time (quadratic) | | |  |  |  | - |  |  |  |  |  |
|  | HELT*time (linear) | | |  |  |  | - |  |  |  |  |  |
|  | HELT*time (quadratic) | | |  |  |  | - |  |  |  |  |  |
| Consumer Price Index (CPI, Lag 1) | | | |  |  |  |  |  | -.0013 | *** |  |  |
| Age*Consumer Price Index | | | |  |  |  |  |  |  |  |  |  |
|  | Age1519*CPI Lag1 | | |  |  |  |  |  |  |  | -.0024 | *** |
|  | Age2024*CPI Lag1 | | |  |  |  |  |  |  |  | -.0022 | *** |
|  | Age2529*CPI Lag1 | | |  |  |  |  |  |  |  | -.0011 | *** |
|  | Age3034*CPI Lag1 | | |  |  |  |  |  |  |  | .0002 | *** |
|  | Age3539*CPI Lag1 | | |  |  |  |  |  |  |  | .0012 | *** |
|  | Age4044*CPI Lag1 | | |  |  |  |  |  |  |  | .0011 | *** |
|  | Age4549*CPI Lag1 | | |  |  |  |  |  |  |  | -.0022 | *** |
| Constant | | | - |  | - |  | - |  | -6.5395 | *** | -6.5317 | *** |
|  | | |  |  |  |  |  |  |  |  |  |  |
| N | | | - | | - | | - | | 47,354,001 | | 47,354,001 | |
| Log Likelihood | | | - | | - | | - | | -8623349.3 | | -8562259.3 | |

*Table A.2 (con’t) Models 0-12 using consumer price index (CPI) as macroeconomic indicator*

|  | |  | **Model 5** | | | **Model 6** | | **Model 7** | | **Model 8** | |
| --- | --- | --- | --- | --- | --- | --- | --- | --- | --- | --- | --- |
|  | |  | **b** | | **sig.** | **b** | **sig.** | **b** | **sig.** | **b** | **sig.** |
| Baseline | | |  | |  |  |  |  |  |  |  |
|  | time (linear) | | .8350 | | *** | .6856 | *** | .8504 | *** | .8176 | *** |
|  | time (quadratic) | | -.0566 | | *** | -.0442 | *** | -.0568 | *** | -.0552 | *** |
|  | time (cubic) | | .0010 | | *** | .0007 | *** | .0010 | *** | .0010 | *** |
| Education | | |  | |  |  |  |  |  |  |  |
|  | LSE | | -.5646 | | *** | -.5016 | *** | -.4471 | *** | -.5779 | *** |
|  | HSE | | -2.0165 | | *** | -1.7860 | *** | -1.4446 | *** | -2.0429 | *** |
|  | HEST | | -4.5931 | | *** | -4.3111 | *** | -3.6927 | *** | -4.6226 | *** |
|  | HELT | | -7.4982 | | *** | -7.1328 | *** | -6.0231 | *** | -7.5141 | *** |
| In education | | | -.8470 | | *** | -.8864 | *** | -.7616 | *** | -.8540 | *** |
| Years graduated | | |  | |  |  |  |  |  |  |  |
|  | Linear | | .0615 | | *** | .0679 | *** | .0763 | *** | .0573 | *** |
|  | Quadratic | | -.0030 | | *** | -.0034 | *** | -.0036 | *** | -.0026 | *** |
| Education*Baseline | | |  | |  |  |  |  |  |  |  |
|  | LSE*time (linear) | | .0773 | | *** | .0643 | *** | .0624 | *** | .0793 | *** |
|  | LSE*time (quadratic) | | -.0025 | | *** | -.0019 | *** | -.0022 | *** | -.0025 | *** |
|  | HSE*time (linear) | | .2551 | | *** | .2256 | *** | .2033 | *** | .2599 | *** |
|  | HSE*time (quadratic) | | -.0073 | | *** | -.0057 | *** | -.0065 | *** | -.0074 | *** |
|  | HEST*time (linear) | | .5584 | | *** | .5081 | *** | .4809 | *** | .5629 | *** |
|  | HEST*time (quadratic) | | -.0151 | | *** | -.0128 | *** | -.0142 | *** | -.0151 | *** |
|  | HELT*time (linear) | | .8233 | | *** | .7708 | *** | .7140 | *** | .8258 | *** |
|  | HELT*time (quadratic) | | -.0204 | | *** | -.0179 | *** | -.0189 | *** | -.0203 | *** |
| Age*Consumer Price Index (Lag 1) | | | | |  |  |  |  |  |  |  |
|  | Age1519*CPI Lag1 | | -.0006 | | *** |  |  |  |  | -.0006 | *** |
|  | Age2024*CPI Lag1 | | -.0011 | | *** |  |  |  |  | -.0011 | *** |
|  | Age2529*CPI Lag1 | | -.0007 | | *** |  |  |  |  | -.0008 | *** |
|  | Age3034*CPI Lag1 | | -.0001 | | *** |  |  |  |  | -.0031 | *** |
|  | Age3539*CPI Lag1 | | .0009 | | *** |  |  |  |  | -.0031 | *** |
|  | Age4044*CPI Lag1 | | .0010 | | *** |  |  |  |  | -.0057 | *** |
|  | Age4549*CPI Lag1 | | -.0037 | | *** |  |  |  |  | -.0235 | *** |
| Education*Consumer Price Index (Lag 1) | | | | |  |  |  |  |  |  |  |
|  | LE*CPI Lag1 | |  | |  | -.0004 | *** |  |  |  |  |
|  | LSE*CPI Lag1 | |  | |  | -.0005 | *** |  |  |  |  |
|  | HSE*CPI Lag1 | |  | |  | -.0009 | *** |  |  |  |  |
|  | HEST*CPI Lag1 | |  | |  | -.0006 | *** |  |  |  |  |
|  | Helt*CPI Lag1 | |  | |  | -.0008 | *** |  |  |  |  |
| Education*Age*Consumer Price Index (Lag 1) | | | | | |  |  |  |  |  |  |
|  | LE*Age1519*CPI Lag1 | | |  |  |  |  | .0004 | *** |  |  |
|  | LE*Age2024*CPI Lag1 | | |  |  |  |  | -.0005 | *** |  |  |
|  | LE*Age2529*CPI Lag1 | | |  |  |  |  | -.0010 | *** |  |  |
|  | LE*Age3034*CPI Lag1 | | |  |  |  |  | -.0001 | ** |  |  |
|  | LE*Age3539*CPI Lag1 | | |  |  |  |  | .0008 | *** |  |  |
|  | LE*Age4044*CPI Lag1 | | |  |  |  |  | -.0006 | *** |  |  |
|  | LE*Age4549*CPI Lag1 | | |  |  |  |  | -.0112 | *** |  |  |
|  | LSE*Age1519*CPI Lag1 | | |  |  |  |  | -.0002 | *** |  |  |
|  | LSE*Age2024*CPI Lag1 | | |  |  |  |  | -.0006 | *** |  |  |
|  | LSE*Age2529*CPI Lag1 | | |  |  |  |  | -.0008 | *** |  |  |
|  | LSE*Age3034*CPI Lag1 | | |  |  |  |  | -.0001 | *** |  |  |
|  | LSE*Age3539*CPI Lag1 | | |  |  |  |  | .0007 | *** |  |  |
|  | LSE*Age4044*CPI Lag1 | | |  |  |  |  | -.0004 | *** |  |  |
|  | LSE*Age4549*CPI Lag1 | | |  |  |  |  | -.0071 | *** |  |  |
|  | HSE*Age1519*CPI Lag1 | | |  |  |  |  | -.0015 | *** |  |  |
|  | HSE*Age2024*CPI Lag1 | | |  |  |  |  | -.0012 | *** |  |  |
|  | HSE*Age2529*CPI Lag1 | | |  |  |  |  | -.0008 | *** |  |  |
|  | HSE*Age3034*CPI Lag1 | | |  |  |  |  | -.0001 | *** |  |  |
|  | HSE*Age3539*CPI Lag1 | | |  |  |  |  | .0009 | *** |  |  |
|  |  | | |  |  |  |  |  |  |  |  |
|  | *continued on next page* | | |  |  |  |  |  |  |  |  |

*Table A.2 (con’t) Models 0-12 using consumer price index (CPI) as macroeconomic indicator*

|  | |  | **Model 5** | | | **Model 6** | | **Model 7** | | **Model 8** | |
| --- | --- | --- | --- | --- | --- | --- | --- | --- | --- | --- | --- |
|  | |  | **b** | | **sig.** | **b** | **sig.** | **b** | **sig.** | **b** | **sig.** |
|  | | |  | |  |  |  |  |  |  |  |
|  | HSE*Age4044*CPI Lag1 | | |  |  |  |  | .0004 | *** |  |  |
|  | HSE*Age4549*CPI Lag1 | | |  |  |  |  | -.0037 | *** |  |  |
|  | HEST*Age1519*CPI Lag1 | | |  |  |  |  | -.0019 | *** |  |  |
|  | HEST*Age2024*CPI Lag1 | | |  |  |  |  | -.0016 | *** |  |  |
|  | HEST*Age2529*CPI Lag1 | | |  |  |  |  | -.0007 | *** |  |  |
|  | HEST*Age3034*CPI Lag1 | | |  |  |  |  | .0000 | *** |  |  |
|  | HEST*Age3539*CPI Lag1 | | |  |  |  |  | .0012 | *** |  |  |
|  | HEST*Age4044*CPI Lag1 | | |  |  |  |  | .0017 | *** |  |  |
|  | HEST*Age4549*CPI Lag1 | | |  |  |  |  | -.0003 | * |  |  |
|  | HELT*Age1519*CPI Lag1 | | |  |  |  |  | -.0019 | *** |  |  |
|  | HELT*Age2024*CPI Lag1 | | |  |  |  |  | -.0029 | *** |  |  |
|  | HELT*Age2529*CPI Lag1 | | |  |  |  |  | -.0011 | *** |  |  |
|  | HELT*Age3034*CPI Lag1 | | |  |  |  |  | -.0003 | *** |  |  |
|  | HELT*Age3539*CPI Lag1 | | |  |  |  |  | .0007 | *** |  |  |
|  | HELT*Age4044*CPI Lag1 | | |  |  |  |  | .0015 | *** |  |  |
|  | HELT*Age4549*CPI Lag1 | | |  |  |  |  | -.0001 |  |  |  |
| Age*Consumer Price Index (Lag 10) | | | | |  |  |  |  |  |  |  |
|  | Age3034*CPI Lag10 | | |  |  |  |  |  |  | .0035 | *** |
|  | Age3539*CPI Lag10 | | |  |  |  |  |  |  | .0046 | *** |
|  | Age4044*CPI Lag10 | | |  |  |  |  |  |  | .0077 | *** |
|  | Age4549*CPI Lag10 | | |  |  |  |  |  |  | .0241 | *** |
| Constant | | | -5.3660 | | *** | -5.0159 | *** | -5.6668 | *** | -5.2991 | *** |
|  | | |  | |  |  |  |  |  |  |  |
| N | | | 47,354,001 | | | 47,354,001 | | 47,354,001 | | 47,354,001 | |
| Log Likelihood | | | -8231377.7 | | | -8245391.7 | | -8221300.4 | | -8225689.5 | |

*Table A.2 (con’t) Models 0-12 using consumer price index (CPI) as macroeconomic indicator*

|  | |  | **Model 9** | | | **Model 10** | | **Model 11** | | **Model 12** | |
| --- | --- | --- | --- | --- | --- | --- | --- | --- | --- | --- | --- |
|  | |  | **b** | | **sig.** | **b** | **sig.** | **b** | **sig.** | **b** | **sig.** |
| Baseline | | |  | |  |  |  |  |  |  |  |
|  | time (linear) | | .8405 | | *** | .8142 | *** | .7720 | *** | .8375 | *** |
|  | time (quadratic) | | -.0560 | | *** | -.0549 | *** | -.0517 | *** | -.0570 | *** |
|  | time (cubic) | | .0010 | | *** | .0009 | *** | .0009 | *** | .0010 | *** |
| Education | | |  | |  |  |  |  |  |  |  |
|  | LSE | | -.4490 | | *** | -.5913 | *** | -.6384 | *** | -.4056 | *** |
|  | HSE | | -1.4410 | | *** | -2.0652 | *** | -2.1381 | *** | -1.6226 | *** |
|  | HEST | | -3.7104 | | *** | -4.6500 | *** | -4.7047 | *** | -4.4621 | *** |
|  | HELT | | -6.1394 | | *** | -7.5410 | *** | -7.5312 | *** | -7.6710 | *** |
| In education | | | -.7676 | | *** | -.8487 | *** | -.8544 | *** | -.7388 | *** |
| Years graduated | | |  | |  |  |  |  |  |  |  |
|  | Linear | | .0713 | | *** | .0560 | *** | .0542 | *** | .0728 | *** |
|  | Quadratic | | -.0032 | | *** | -.0026 | *** | -.0025 | *** | -.0032 | *** |
| Education*Baseline | | |  | |  |  |  |  |  |  |  |
|  | LSE*time (linear) | | .0617 | | *** | .0785 | *** | .0901 | *** | .0627 | *** |
|  | LSE*time (quadratic) | | -.0020 | | *** | -.0024 | *** | -.0029 | *** | -.0018 | *** |
|  | HSE*time (linear) | | .1997 | | *** | .2600 | *** | .2774 | *** | .2287 | *** |
|  | HSE*time (quadratic) | | -.0060 | | *** | -.0073 | *** | -.0081 | *** | -.0060 | *** |
|  | HEST*time (linear) | | .4799 | | *** | .5634 | *** | .5784 | *** | .5588 | *** |
|  | HEST*time (quadratic) | | -.0136 | | *** | -.0151 | *** | -.0158 | *** | -.0145 | *** |
|  | HELT*time (linear) | | .7306 | | *** | .8261 | *** | .8325 | *** | .8891 | *** |
|  | HELT*time (quadratic) | | -.0185 | | *** | -.0203 | *** | -.0207 | *** | -.0215 | *** |
| Age*Consumer Price Index (Lag 1) | | | | |  |  |  |  |  |  |  |
|  | Age1519*CPI Lag1 | |  | |  | -.0008 | *** |  |  |  |  |
|  | Age2024*CPI Lag1 | |  | |  | -.0012 | *** |  |  |  |  |
|  | Age2529*CPI Lag1 | |  | |  | -.0010 | *** |  |  |  |  |
|  | Age3034*CPI Lag1 | |  | |  | -.0035 | *** |  |  |  |  |
|  | Age3539*CPI Lag1 | |  | |  | -.0036 | *** |  |  |  |  |
|  | Age4044*CPI Lag1 | |  | |  | -.0063 | *** |  |  |  |  |
|  | Age4549*CPI Lag1 | |  | |  | -.0245 | *** |  |  |  |  |
| Education*Age*Consumer Price Index (Lag 1) | | | | | |  |  |  |  |  |  |
|  | LE*Age1519*CPI Lag1 | | | .0004 | *** |  |  |  |  |  |  |
|  | LE*Age2024*CPI Lag1 | | | -.0005 | *** |  |  |  |  |  |  |
|  | LE*Age2529*CPI Lag1 | | | -.0010 | *** |  |  |  |  |  |  |
|  | LE*Age3034*CPI Lag1 | | | -.0032 | *** |  |  |  |  |  |  |
|  | LE*Age3539*CPI Lag1 | | | -.0042 | *** |  |  |  |  |  |  |
|  | LE*Age4044*CPI Lag1 | | | -.0080 | *** |  |  |  |  |  |  |
|  | LE*Age4549*CPI Lag1 | | | -.0210 | *** |  |  |  |  |  |  |
|  | LSE*Age1519*CPI Lag1 | | | -.0002 | *** |  |  |  |  |  |  |
|  | LSE*Age2024*CPI Lag1 | | | -.0006 | *** |  |  |  |  |  |  |
|  | LSE*Age2529*CPI Lag1 | | | -.0008 | *** |  |  |  |  |  |  |
|  | LSE*Age3034*CPI Lag1 | | | -.0020 | *** |  |  |  |  |  |  |
|  | LSE*Age3539*CPI Lag1 | | | -.0021 | *** |  |  |  |  |  |  |
|  | LSE*Age4044*CPI Lag1 | | | -.0056 | *** |  |  |  |  |  |  |
|  | LSE*Age4549*CPI Lag1 | | | -.0232 | *** |  |  |  |  |  |  |
|  | HSE*Age1519*CPI Lag1 | | | -.0015 | *** |  |  |  |  |  |  |
|  | HSE*Age2024*CPI Lag1 | | | -.0012 | *** |  |  |  |  |  |  |
|  | HSE*Age2529*CPI Lag1 | | | -.0009 | *** |  |  |  |  |  |  |
|  | HSE*Age3034*CPI Lag1 | | | -.0028 | *** |  |  |  |  |  |  |
|  | HSE*Age3539*CPI Lag1 | | | -.0029 | *** |  |  |  |  |  |  |
|  | HSE*Age4044*CPI Lag1 | | | -.0052 | *** |  |  |  |  |  |  |
|  | HSE*Age4549*CPI Lag1 | | | -.0177 | *** |  |  |  |  |  |  |
|  | HEST*Age1519*CPI Lag1 | | | -.0019 | *** |  |  |  |  |  |  |
|  | HEST*Age2024*CPI Lag1 | | | -.0017 | *** |  |  |  |  |  |  |
|  | HEST*Age2529*CPI Lag1 | | | -.0008 | *** |  |  |  |  |  |  |
|  | HEST*Age3034*CPI Lag1 | | | -.0037 | *** |  |  |  |  |  |  |
|  |  | | |  |  |  |  |  |  |  |  |
|  | *continued on next page* | | |  |  |  |  |  |  |  |  |

*Table A.2 (con’t) Models 0-12 using consumer price index (CPI) as macroeconomic indicator*

|  | |  | **Model 9** | | | **Model 10** | | **Model 11** | | **Model 12** | |
| --- | --- | --- | --- | --- | --- | --- | --- | --- | --- | --- | --- |
|  | |  | **b** | | **sig.** | **b** | **sig.** | **b** | **sig.** | **b** | **sig.** |
|  | HEST*Age3539* CPI Lag1 | | | -.0033 | *** |  |  |  |  |  |  |
|  | HEST*Age4044* CPI Lag1 | | | -.0035 | *** |  |  |  |  |  |  |
|  | HEST*Age4549* CPI Lag1 | | | -.0093 | *** |  |  |  |  |  |  |
|  | HELT*Age1519* CPI Lag1 | | | -.0019 | *** |  |  |  |  |  |  |
|  | HELT*Age2024* CPI Lag1 | | | -.0031 | *** |  |  |  |  |  |  |
|  | HELT*Age2529* CPI Lag1 | | | -.0014 | *** |  |  |  |  |  |  |
|  | HELT*Age3034* CPI Lag1 | | | -.0055 | *** |  |  |  |  |  |  |
|  | HELT*Age3539* CPI Lag1 | | | -.0061 | *** |  |  |  |  |  |  |
|  | HELT*Age4044* CPI Lag1 | | | -.0067 | *** |  |  |  |  |  |  |
|  | HELT*Age4549* CPI Lag1 | | | -.0135 | *** |  |  |  |  |  |  |
| Age*Consumer Price Index (Lag 10) | | | | |  |  |  |  |  |  |  |
|  | Age3034*CPI Lag10 | | |  |  | .0039 | *** |  |  |  |  |
|  | Age3539*CPI Lag10 | | |  |  | .0049 | *** |  |  |  |  |
|  | Age4044*CPI Lag10 | | |  |  | .0082 | *** |  |  |  |  |
|  | Age4549*CPI Lag10 | | |  |  | .0252 | *** |  |  |  |  |
| Age*Consumer Price Index (Lag 10) | | | | |  |  |  |  |  |  |  |
|  | LE*Age3034*CPI Lag10 | | | .0041 | *** |  |  |  |  |  |  |
|  | LE*Age3539*CPI Lag10 | | | .0064 | *** |  |  |  |  |  |  |
|  | LE*Age4044*CPI Lag10 | | | .0095 | *** |  |  |  |  |  |  |
|  | LE*Age4549*CPI Lag10 | | | .0136 | *** |  |  |  |  |  |  |
|  | LSE*Age3034*CPI Lag10 | | | .0025 | *** |  |  |  |  |  |  |
|  | LSE*Age3539*CPI Lag10 | | | .0033 | *** |  |  |  |  |  |  |
|  | LSE*Age4044*CPI Lag10 | | | .0063 | *** |  |  |  |  |  |  |
|  | LSE*Age4549*CPI Lag10 | | | .0208 | *** |  |  |  |  |  |  |
|  | HSE*Age3034*CPI Lag10 | | | .0032 | *** |  |  |  |  |  |  |
|  | HSE*Age3539*CPI Lag10 | | | .0042 | *** |  |  |  |  |  |  |
|  | HSE*Age4044*CPI Lag10 | | | .0064 | *** |  |  |  |  |  |  |
|  | HSE*Age4549*CPI Lag10 | | | .0165 | *** |  |  |  |  |  |  |
|  | HEST*Age3034*CPI Lag10 | | | .0043 | *** |  |  |  |  |  |  |
|  | HEST*Age3539*CPI Lag10 | | | .0050 | *** |  |  |  |  |  |  |
|  | HEST*Age4044*CPI Lag10 | | | .0056 | *** |  |  |  |  |  |  |
|  | HEST*Age4549*CPI Lag10 | | | .0100 | *** |  |  |  |  |  |  |
|  | HELT*Age3034*CPI Lag10 | | | .0058 | *** |  |  |  |  |  |  |
|  | HELT*Age3539*CPI Lag10 | | | .0075 | *** |  |  |  |  |  |  |
|  | HELT*Age4044*CPI Lag10 | | | .0088 | *** |  |  |  |  |  |  |
|  | HELT*Age4549*CPI Lag10 | | | .0148 |  |  |  |  |  |  |  |
| Period*Consumer Price Index (Lag 1) | | | | |  |  |  |  |  |  |  |
|  | 19601973*CPI Lag1 | | |  |  | .0070 | *** |  |  |  |  |
|  | 19741991*CPI Lag1 | | |  |  | .0000 |  |  |  |  |  |
|  | 19922010*CPI Lag1 | | |  |  | .0000 |  |  |  |  |  |
| Period*Consumer Price Index (Lag 10) | | | | |  |  |  |  |  |  |  |
|  | 19601973*CPI Lag10 | | |  |  | -.0092 | *** |  |  |  |  |
|  | 19741991*CPI Lag10 | | |  |  | .0000 |  |  |  |  |  |
|  | 19922010*CPI Lag10 | | |  |  | .0001 |  |  |  |  |  |
| Period*Age*Consumer Price Index (Lag 1) | | | | | |  |  |  |  |  |  |
|  | 19601973*Age1519*CPI Lag1 | | | |  |  |  | -.0015 | *** |  |  |
|  | 19601973*Age2024*CPI Lag1 | | | |  |  |  | -.0003 | *** |  |  |
|  | 19601973*Age2529*CPI Lag1 | | | |  |  |  | -.0014 | *** |  |  |
|  | 19601973*Age3034*CPI Lag1 | | | |  |  |  | -.0034 | *** |  |  |
|  | 19601973*Age3539*CPI Lag1 | | | |  |  |  | -.0056 | *** |  |  |
|  | 19601973*Age4044*CPI Lag1 | | | |  |  |  | -.0091 | *** |  |  |
|  | 19601973*Age4549*CPI Lag1 | | | |  |  |  | -.0099 |  |  |  |
|  | 19741991*Age1519*CPI Lag1 | | | |  |  |  | -.0006 | *** |  |  |
|  | 19741991*Age2024*CPI Lag1 | | | |  |  |  | -.0009 | *** |  |  |
|  | 19741991*Age2529*CPI Lag1 | | | |  |  |  | -.0008 | *** |  |  |
|  | 19741991*Age3034*CPI Lag1 | | | |  |  |  | -.0045 | *** |  |  |
|  |  | | | |  |  |  |  |  |  |  |
|  | *continued on next page* | | | |  |  |  |  |  |  |  |

*Table A.2 (con’t) Models 0-12 using consumer price index (CPI) as macroeconomic indicator*

|  | |  | **Model 9** | | **Model 10** | | **Model 11** | | **Model 12** | |
| --- | --- | --- | --- | --- | --- | --- | --- | --- | --- | --- |
|  | |  | **b** | **sig.** | **b** | **sig.** | **b** | **sig.** | **b** | **sig.** |
|  | 19741991*Age3539*CPI Lag1 | | |  |  |  | -.0049 | *** |  |  |
|  | 19741991*Age4044*CPI Lag1 | | |  |  |  | -.0088 | *** |  |  |
|  | 19741991*Age4549*CPI Lag1 | | |  |  |  | -.0189 | *** |  |  |
|  | 19922010*Age1519*CPI Lag1 | | |  |  |  | -.0005 | *** |  |  |
|  | 19922010*Age2024*CPI Lag1 | | |  |  |  | -.0011 | *** |  |  |
|  | 19922010*Age2529*CPI Lag1 | | |  |  |  | -.0008 | *** |  |  |
|  | 19922010*Age3034*CPI Lag1 | | |  |  |  | -.0030 | *** |  |  |
|  | 19922010*Age3539*CPI Lag1 | | |  |  |  | -.0037 | *** |  |  |
|  | 19922010*Age4044*CPI Lag1 | | |  |  |  | -.0053 | *** |  |  |
|  | 19922010*Age4549*CPI Lag1 | | |  |  |  | -.0128 | *** |  |  |
| Period*Age*Consumer Price Index (Lag 10) | | | |  |  |  |  |  |  |  |
| 19601973*Age3034*CPI Lag10 | | | |  |  |  | .0019 | ** |  |  |
| 19601973*Age3539*CPI Lag10 | | | |  |  |  | .0068 | *** |  |  |
| 19601973*Age4044*CPI Lag10 | | | |  |  |  | .0085 | ** |  |  |
| 19601973*Age4549*CPI Lag10 | | | |  |  |  | -.0133 |  |  |  |
| 19741991*Age3034*CPI Lag10 | | | |  |  |  | .0055 | *** |  |  |
| 19741991*Age3539*CPI Lag10 | | | |  |  |  | .0067 | *** |  |  |
| 19741991*Age4044*CPI Lag10 | | | |  |  |  | .0113 | *** |  |  |
| 19741991*Age4549*CPI Lag10 | | | |  |  |  | .0139 | ** |  |  |
| 19922010*Age3034*CPI Lag10 | | | |  |  |  | .0032 | *** |  |  |
| 19922010*Age3539*CPI Lag10 | | | |  |  |  | .0049 | *** |  |  |
| 19922010*Age4044*CPI Lag10 | | | |  |  |  | .0067 | *** |  |  |
| 19922010*Age4549*CPI Lag10 | | | |  |  |  | .0112 | *** |  |  |
| Period*Age*Education*Consumer Price Index (Lag 1) | | | | | | | | | | |
| LE*Age1519*19601973*CPI Lag1 | | | |  |  |  |  |  | -.0000 |  |
| LE*Age1519*19741991*CPI Lag1 | | | |  |  |  |  |  | .0003 | *** |
| LE*Age1519*19922010*CPI Lag1 | | | |  |  |  |  |  | .0005 | *** |
| LE*Age2024*19601973*CPI Lag1 | | | |  |  |  |  |  | .0002 | * |
| LE*Age2024*19741991*CPI Lag1 | | | |  |  |  |  |  | -.0007 | *** |
| LE*Age2024*19922010*CPI Lag1 | | | |  |  |  |  |  | -.0003 | *** |
| LE*Age2529*19601973*CPI Lag1 | | | |  |  |  |  |  | -.0005 | *** |
| LE*Age2529*19741991*CPI Lag1 | | | |  |  |  |  |  | -.0010 | *** |
| LE*Age2529*19922010*CPI Lag1 | | | |  |  |  |  |  | -.0005 | *** |
| LE*Age3034*19601973*CPI Lag1 | | | |  |  |  |  |  | -.0067 | *** |
| LE*Age3034*19741991*CPI Lag1 | | | |  |  |  |  |  | -.0023 | *** |
| LE*Age3034*19922010*CPI Lag1 | | | |  |  |  |  |  | -.0036 | *** |
| LE*Age3539*19601973*CPI Lag1 | | | |  |  |  |  |  | -.0086 | *** |
| LE*Age3539*19741991*CPI Lag1 | | | |  |  |  |  |  | -.0032 | *** |
| LE*Age3539*19922010*CPI Lag1 | | | |  |  |  |  |  | -.0040 | *** |
| LE*Age4044*19601973*CPI Lag1 | | | |  |  |  |  |  | -.0163 | *** |
| LE*Age4044*19741991*CPI Lag1 | | | |  |  |  |  |  | -.0071 | *** |
| LE*Age4044*19922010*CPI Lag1 | | | |  |  |  |  |  | -.0091 | *** |
| LE*Age4549*19601973*CPI Lag1 | | | |  |  |  |  |  | -.0056 |  |
| LE*Age4549*19741991*CPI Lag1 | | | |  |  |  |  |  | -.0169 | *** |
| LE*Age4549*19922010*CPI Lag1 | | | |  |  |  |  |  | -.0209 | ** |
| LSE*Age1519*19601973*CPI Lag1 | | | |  |  |  |  |  | -.0014 | *** |
| LSE*Age1519*19741991*CPI Lag1 | | | |  |  |  |  |  | -.0003 | *** |
| LSE*Age1519*19922010*CPI Lag1 | | | |  |  |  |  |  | -.0002 | *** |
| LSE*Age2024*19601973*CPI Lag1 | | | |  |  |  |  |  | -.0005 | *** |
| LSE*Age2024*19741991*CPI Lag1 | | | |  |  |  |  |  | -.0008 | *** |
| LSE*Age2024*19922010*CPI Lag1 | | | |  |  |  |  |  | -.0007 | *** |
| LSE*Age2529*19601973*CPI Lag1 | | | |  |  |  |  |  | -.0010 | *** |
| LSE*Age2529*19741991*CPI Lag1 | | | |  |  |  |  |  | -.0008 | *** |
| LSE*Age2529*19922010*CPI Lag1 | | | |  |  |  |  |  | -.0007 | *** |
| LSE*Age3034*19601973*CPI Lag1 | | | |  |  |  |  |  | -.0037 | *** |
| LSE*Age3034*19741991*CPI Lag1 | | | |  |  |  |  |  | -.0028 | *** |
|  | | | |  |  |  |  |  |  |  |
| *continued on next page* | | | |  |  |  |  |  |  |  |

*Table A.2 (con’t) Models 0-12 using consumer price index (CPI) as macroeconomic indicator*

|  |  | **Model 9** | | **Model 10** | | **Model 11** | | **Model 12** | |
| --- | --- | --- | --- | --- | --- | --- | --- | --- | --- |
|  |  | **b** | **sig.** | **b** | **sig.** | **b** | **sig.** | **b** | **sig.** |
| LSE*Age3034*19922010*CPI Lag1 | | |  |  |  |  |  | -.0011 | ** |
| LSE*Age3539*19601973*CPI Lag1 | | |  |  |  |  |  | -.0068 | *** |
| LSE*Age3539*19741991*CPI Lag1 | | |  |  |  |  |  | -.0022 | *** |
| LSE*Age3539*19922010*CPI Lag1 | | |  |  |  |  |  | -.0011 |  |
| LSE*Age4044*19601973*CPI Lag1 | | |  |  |  |  |  | -.0073 |  |
| LSE*Age4044*19741991*CPI Lag1 | | |  |  |  |  |  | -.0085 | *** |
| LSE*Age4044*19922010*CPI Lag1 | | |  |  |  |  |  | -.0030 | * |
| LSE*Age4549*19601973*CPI Lag1 | | |  |  |  |  |  | -.0256 |  |
| LSE*Age4549*19741991*CPI Lag1 | | |  |  |  |  |  | -.0219 | *** |
| LSE*Age4549*19922010*CPI Lag1 | | |  |  |  |  |  | -.0016 |  |
| HSE*Age1519*19601973*CPI Lag1 | | |  |  |  |  |  | -.0014 | *** |
| HSE*Age1519*19741991*CPI Lag1 | | |  |  |  |  |  | -.0015 | *** |
| HSE*Age1519*19922010*CPI Lag1 | | |  |  |  |  |  | -.0014 | *** |
| HSE*Age2024*19601973*CPI Lag1 | | |  |  |  |  |  | -.0009 | *** |
| HSE*Age2024*19741991*CPI Lag1 | | |  |  |  |  |  | -.0012 | *** |
| HSE*Age2024*19922010*CPI Lag1 | | |  |  |  |  |  | -.0013 | *** |
| HSE*Age2529*19601973*CPI Lag1 | | |  |  |  |  |  | -.0024 | *** |
| HSE*Age2529*19741991*CPI Lag1 | | |  |  |  |  |  | -.0011 | *** |
| HSE*Age2529*19922010*CPI Lag1 | | |  |  |  |  |  | -.0010 | *** |
| HSE*Age3034*19601973*CPI Lag1 | | |  |  |  |  |  | .0025 | * |
| HSE*Age3034*19741991*CPI Lag1 | | |  |  |  |  |  | -.0046 | *** |
| HSE*Age3034*19922010*CPI Lag1 | | |  |  |  |  |  | -.0030 | *** |
| HSE*Age3539*19601973*CPI Lag1 | | |  |  |  |  |  | -.0050 | * |
| HSE*Age3539*19741991*CPI Lag1 | | |  |  |  |  |  | -.0047 | *** |
| HSE*Age3539*19922010*CPI Lag1 | | |  |  |  |  |  | -.0037 | *** |
| HSE*Age4044*19601973*CPI Lag1 | | |  |  |  |  |  | -.0095 |  |
| HSE*Age4044*19741991*CPI Lag1 | | |  |  |  |  |  | -.0082 | *** |
| HSE*Age4044*19922010*CPI Lag1 | | |  |  |  |  |  | -.0044 | *** |
| HSE*Age4549*19601973*CPI Lag1 | | |  |  |  |  |  | -.0149 |  |
| HSE*Age4549*19741991*CPI Lag1 | | |  |  |  |  |  | -.0120 | * |
| HSE*Age4549*19922010*CPI Lag1 | | |  |  |  |  |  | -.0095 | * |
| HEST*Age1519*19601973*CPI Lag1 | | |  |  |  |  |  | .0011 | *** |
| HEST*Age1519*19741991*CPI Lag1 | | |  |  |  |  |  | -.0011 | *** |
| HEST*Age1519*19922010*CPI Lag1 | | |  |  |  |  |  | -.0011 | *** |
| HEST*Age2024*19601973*CPI Lag1 | | |  |  |  |  |  | .0014 | *** |
| HEST*Age2024*19741991*CPI Lag1 | | |  |  |  |  |  | -.0010 | *** |
| HEST*Age2024*19922010*CPI Lag1 | | |  |  |  |  |  | -.0016 | *** |
| HEST*Age2529*19601973*CPI Lag1 | | |  |  |  |  |  | -.0015 | *** |
| HEST*Age2529*19741991*CPI Lag1 | | |  |  |  |  |  | -.0007 | *** |
| HEST*Age2529*19922010*CPI Lag1 | | |  |  |  |  |  | -.0008 | *** |
| HEST*Age3034*19601973*CPI Lag1 | | |  |  |  |  |  | .0109 | *** |
| HEST*Age3034*19741991*CPI Lag1 | | |  |  |  |  |  | -.0059 | *** |
| HEST*Age3034*19922010*CPI Lag1 | | |  |  |  |  |  | -.0038 | *** |
| HEST*Age3539*19601973*CPI Lag1 | | |  |  |  |  |  | .0134 | *** |
| HEST*Age3539*19741991*CPI Lag1 | | |  |  |  |  |  | -.0064 | *** |
| HEST*Age3539*19922010*CPI Lag1 | | |  |  |  |  |  | -.0038 | *** |
| HEST*Age4044*19601973*CPI Lag1 | | |  |  |  |  |  | -.0012 |  |
| HEST*Age4044*19741991*CPI Lag1 | | |  |  |  |  |  | -.0074 | *** |
| HEST*Age4044*19922010*CPI Lag1 | | |  |  |  |  |  | -.0022 | * |
| HEST*Age4549*19601973*CPI Lag1 | | |  |  |  |  |  | -.0204 |  |
| HEST*Age4549*19741991*CPI Lag1 | | |  |  |  |  |  | -.0157 | ** |
| HEST*Age4549*19922010*CPI Lag1 | | |  |  |  |  |  | -.0039 |  |
| HELT*Age1519*19601973*CPI Lag1 | | |  |  |  |  |  | .0101 | *** |
| HELT*Age1519*19741991*CPI Lag1 | | |  |  |  |  |  | .0009 | *** |
| HELT*Age1519*19922010*CPI Lag1 | | |  |  |  |  |  | -.0013 | *** |
| HELT*Age2024*19601973*CPI Lag1 | | |  |  |  |  |  | .0045 | *** |
|  | | |  |  |  |  |  |  |  |
| *continued on next page* | | |  |  |  |  |  |  |  |

*Table A.2 (con’t) Models 0-12 using consumer price index (CPI) as macroeconomic indicator*

|  |  | **Model 9** | | **Model 10** | | **Model 11** | | **Model 12** | |
| --- | --- | --- | --- | --- | --- | --- | --- | --- | --- |
|  |  | **b** | **sig.** | **b** | **sig.** | **b** | **sig.** | **b** | **sig.** |
| HELT*Age2024*19741991*CPI Lag1 | | |  |  |  |  |  | -.0012 | *** |
| HELT*Age2024*19922010*CPI Lag1 | | |  |  |  |  |  | -.0030 | *** |
| HELT*Age2529*19601973*CPI Lag1 | | |  |  |  |  |  | -.0007 | *** |
| HELT*Age2529*19741991*CPI Lag1 | | |  |  |  |  |  | -.0011 | *** |
| HELT*Age2529*19922010*CPI Lag1 | | |  |  |  |  |  | -.0013 | *** |
| HELT*Age3034*19601973*CPI Lag1 | | |  |  |  |  |  | .0127 | *** |
| HELT*Age3034*19741991*CPI Lag1 | | |  |  |  |  |  | -.0075 | *** |
| HELT*Age3034*19922010*CPI Lag1 | | |  |  |  |  |  | -.0050 | *** |
| HELT*Age3539*19601973*CPI Lag1 | | |  |  |  |  |  | .0065 |  |
| HELT*Age3539*19741991*CPI Lag1 | | |  |  |  |  |  | -.0092 | *** |
| HELT*Age3539*19922010*CPI Lag1 | | |  |  |  |  |  | -.0069 | *** |
| HELT*Age4044*19601973*CPI Lag1 | | |  |  |  |  |  | .0272 |  |
| HELT*Age4044*19741991*CPI Lag1 | | |  |  |  |  |  | -.0095 | *** |
| HELT*Age4044*19922010*CPI Lag1 | | |  |  |  |  |  | -.0061 | *** |
| HELT*Age4549*19601973*CPI Lag1 | | |  |  |  |  |  | -.0391 |  |
| HELT*Age4549*19741991*CPI Lag1 | | |  |  |  |  |  | -.0200 | * |
| HELT*Age4549*19922010*CPI Lag1 | | |  |  |  |  |  | -.0066 |  |
| LE*Age3034*19601973*CPI Lag10 | | |  |  |  |  |  | .0104 | *** |
| LE*Age3034*19741991*CPI Lag10 | | |  |  |  |  |  | .0034 | *** |
| LE*Age3034*19922010*CPI Lag10 | | |  |  |  |  |  | .0049 | ** |
| LE*Age3539*19601973*CPI Lag10 | | |  |  |  |  |  | .0160 | *** |
| LE*Age3539*19741991*CPI Lag10 | | |  |  |  |  |  | .0059 | *** |
| LE*Age3539*19922010*CPI Lag10 | | |  |  |  |  |  | .0064 | *** |
| LE*Age4044*19601973*CPI Lag10 | | |  |  |  |  |  | .0215 | *** |
| LE*Age4044*19741991*CPI Lag10 | | |  |  |  |  |  | .0094 | *** |
| LE*Age4044*19922010*CPI Lag10 | | |  |  |  |  |  | .0113 | *** |
| LE*Age4549*19601973*CPI Lag10 | | |  |  |  |  |  | -.0162 |  |
| LE*Age4549*19741991*CPI Lag10 | | |  |  |  |  |  | .0087 |  |
| LE*Age4549*19922010*CPI Lag10 | | |  |  |  |  |  | .0192 | * |
| LSE*Age3034*19601973*CPI Lag10 | | |  |  |  |  |  | .0052 | *** |
| LSE*Age3034*19741991*CPI Lag10 | | |  |  |  |  |  | .0040 | *** |
| LSE*Age3034*19922010*CPI Lag10 | | |  |  |  |  |  | .0014 | ** |
| LSE*Age3539*19601973*CPI Lag10 | | |  |  |  |  |  | .0119 | *** |
| LSE*Age3539*19741991*CPI Lag10 | | |  |  |  |  |  | .0037 | *** |
| LSE*Age3539*19922010*CPI Lag10 | | |  |  |  |  |  | .0022 | ** |
| LSE*Age4044*19601973*CPI Lag10 | | |  |  |  |  |  | .0091 |  |
| LSE*Age4044*19741991*CPI Lag10 | | |  |  |  |  |  | .0111 | *** |
| LSE*Age4044*19922010*CPI Lag10 | | |  |  |  |  |  | .0031 |  |
| LSE*Age4549*19601973*CPI Lag10 | | |  |  |  |  |  | .0073 |  |
| LSE*Age4549*19741991*CPI Lag10 | | |  |  |  |  |  | .0177 |  |
| LSE*Age4549*19922010*CPI Lag10 | | |  |  |  |  |  | -.0045 |  |
| HSE*Age3034*19601973*CPI Lag10 | | |  |  |  |  |  | -.0066 | *** |
| HSE*Age3034*19741991*CPI Lag10 | | |  |  |  |  |  | .0055 | *** |
| HSE*Age3034*19922010*CPI Lag10 | | |  |  |  |  |  | .0032 | *** |
| HSE*Age3539*19601973*CPI Lag10 | | |  |  |  |  |  | .0052 | * |
| HSE*Age3539*19741991*CPI Lag10 | | |  |  |  |  |  | .0062 | *** |
| HSE*Age3539*19922010*CPI Lag10 | | |  |  |  |  |  | .0047 | *** |
| HSE*Age4044*19601973*CPI Lag10 | | |  |  |  |  |  | .0085 |  |
| HSE*Age4044*19741991*CPI Lag10 | | |  |  |  |  |  | .0096 | *** |
| HSE*Age4044*19922010*CPI Lag10 | | |  |  |  |  |  | .0047 | ** |
| HSE*Age4549*19601973*CPI Lag10 | | |  |  |  |  |  | -.0087 |  |
| HSE*Age4549*19741991*CPI Lag10 | | |  |  |  |  |  | .0031 |  |
| HSE*Age4549*19922010*CPI Lag10 | | |  |  |  |  |  | .0058 |  |
| HEST*Age3034*19601973*CPI Lag10 | | |  |  |  |  |  | -.0207 | *** |
| HEST*Age3034*19741991*CPI Lag10 | | |  |  |  |  |  | .0072 | *** |
| HEST*Age3034*19922010*CPI Lag10 | | |  |  |  |  |  | .0042 | *** |
|  | | |  |  |  |  |  |  |  |
| *continued on next page* | | |  |  |  |  |  |  |  |

*Table A.2 (con’t) Models 0-12 using consumer price index (CPI) as macroeconomic indicator*

|  |  | **Model 9** | | **Model 10** | | **Model 11** | | **Model 12** | |
| --- | --- | --- | --- | --- | --- | --- | --- | --- | --- |
|  |  | **b** | **sig.** | **b** | **sig.** | **b** | **sig.** | **b** | **sig.** |
| HEST*Age3539*19601973*CPI Lag10 | | |  |  |  |  |  | -.0229 | *** |
| HEST*Age3539*19741991*CPI Lag10 | | |  |  |  |  |  | .0086 | *** |
| HEST*Age3539*19922010*CPI Lag10 | | |  |  |  |  |  | .0049 | *** |
| HEST*Age4044*19601973*CPI Lag10 | | |  |  |  |  |  | -.0042 |  |
| HEST*Age4044*19741991*CPI Lag10 | | |  |  |  |  |  | .0098 | *** |
| HEST*Age4044*19922010*CPI Lag10 | | |  |  |  |  |  | .0031 | * |
| HEST*Age4549*19601973*CPI Lag10 | | |  |  |  |  |  | .0064 |  |
| HEST*Age4549*19741991*CPI Lag10 | | |  |  |  |  |  | .0171 | * |
| HEST*Age4549*19922010*CPI Lag10 | | |  |  |  |  |  | .0023 |  |
| HELT*Age3034*19601973*CPI Lag10 | | |  |  |  |  |  | -.0268 | *** |
| HELT*Age3034*19741991*CPI Lag10 | | |  |  |  |  |  | .0085 | *** |
| HELT*Age3034*19922010*CPI Lag10 | | |  |  |  |  |  | .0050 | *** |
| HELT*Age3539*19601973*CPI Lag10 | | |  |  |  |  |  | -.0197 | ** |
| HELT*Age3539*19741991*CPI Lag10 | | |  |  |  |  |  | .0115 | *** |
| HELT*Age3539*19922010*CPI Lag10 | | |  |  |  |  |  | .0080 | *** |
| HELT*Age4044*19601973*CPI Lag10 | | |  |  |  |  |  | -.0457 | * |
| HELT*Age4044*19741991*CPI Lag10 | | |  |  |  |  |  | .0121 | *** |
| HELT*Age4044*19922010*CPI Lag10 | | |  |  |  |  |  | .0075 | *** |
| HELT*Age4549*19601973*CPI Lag10 | | |  |  |  |  |  | .0324 |  |
| HELT*Age4549*19741991*CPI Lag10 | | |  |  |  |  |  | .0235 |  |
| HELT*Age4549*19922010*CPI Lag10 | | |  |  |  |  |  | .0062 |  |
| Constant | | -5.6226 | *** | -5.2048 | *** | -5.1569 | *** | -5.5926 | *** |
|  | |  |  |  |  |  |  |  |  |
| N | | 47,354,001 | | 47,354,001 | | 47,354,001 | | 47,354,001 | |
| Log Likelihood | | -8214683.2 | | -8223922.8 | | -8221909.7 | | -8205214.7 | |

*Table A.3 Models 3-7 using gross domestic product (GDP) as macroeconomic indicator.*

|  | |  | **Model 0** | | **Model 1** | | **Model 2** | | **Model 3** | | **Model 4** | |
| --- | --- | --- | --- | --- | --- | --- | --- | --- | --- | --- | --- | --- |
|  | |  | **b** | **sig.** | **b** | **sig.** | **b** | **sig.** | **b** | **sig.** | **b** | **sig.** |
| Baseline | | |  |  |  |  |  |  |  |  |  |  |
|  | time (linear) | | - |  | - |  | - |  | .8172 | *** | .8357 | *** |
|  | time (quadratic) | | - |  | - |  | - |  | -.0392 | *** | -.0403 | *** |
|  | time (cubic) | | - |  | - |  | - |  | .0004 | *** | .0005 | *** |
| Education | | |  |  |  |  |  |  |  |  |  |  |
|  | LSE | |  |  | - |  | - |  |  |  |  |  |
|  | HSE | |  |  | - |  | - |  |  |  |  |  |
|  | HEST | |  |  | - |  | - |  |  |  |  |  |
|  | HELT | |  |  | - |  | - |  |  |  |  |  |
| In education | | |  |  | - |  | - |  |  |  |  |  |
| Years graduated | | |  |  |  |  |  |  |  |  |  |  |
|  | Linear | |  |  | - |  | - |  |  |  |  |  |
|  | Quadratic | |  |  | - |  | - |  |  |  |  |  |
| Education*Baseline | | |  |  |  |  |  |  |  |  |  |  |
|  | LSE*time (linear) | | |  |  |  | - |  |  |  |  |  |
|  | LSE*time (quadratic) | | |  |  |  | - |  |  |  |  |  |
|  | HSE*time (linear) | | |  |  |  | - |  |  |  |  |  |
|  | HSE*time (quadratic) | | |  |  |  | - |  |  |  |  |  |
|  | HEST*time (linear) | | |  |  |  | - |  |  |  |  |  |
|  | HEST*time (quadratic) | | |  |  |  | - |  |  |  |  |  |
|  | HELT*time (linear) | | |  |  |  | - |  |  |  |  |  |
|  | HELT*time (quadratic) | | |  |  |  | - |  |  |  |  |  |
| Grosss Domestic Product (GDP, Lag 1) | | | | | | |  |  | .0001 | *** |  |  |
| Age*Gross Domestic Product (GDP, Lag 1) | | | | | | |  |  |  |  |  |  |
|  | Age1519*GDP Lag1 | | |  |  |  |  |  |  |  | .0001 | *** |
|  | Age2024*GDP Lag1 | | |  |  |  |  |  |  |  | .0001 | *** |
|  | Age2529*GDP Lag1 | | |  |  |  |  |  |  |  | .0000 | *** |
|  | Age3034*GDP Lag1 | | |  |  |  |  |  |  |  | -.0000 | * |
|  | Age3539*GDP Lag1 | | |  |  |  |  |  |  |  | .0000 | *** |
|  | Age4044*GDP Lag1 | | |  |  |  |  |  |  |  | -.0001 | *** |
|  | Age4549*GDP Lag1 | | |  |  |  |  |  |  |  | -.0010 | *** |
| Constant | | | - |  | - |  | - |  | -6.9321 | *** | -7.0448 | *** |
|  | | |  |  |  |  |  |  |  |  |  |  |
| N | | | - | | - | | - | | 46,137,850 | | 46,137,850 | |
| Log Likelihood | | | - | | - | | - | | -8429733.6 | | -8428600.3 | |

*Table A.3 (con’t) Models 3-7 using gross domestic product (GDP) as macroeconomic indicator.*

|  | |  | **Model 5** | | | **Model 6** | | **Model 7** | |  | |
| --- | --- | --- | --- | --- | --- | --- | --- | --- | --- | --- | --- |
|  | |  | **b** | | **sig.** | **b** | **sig.** | **b** | **sig.** |  |  |
| Baseline | | |  | |  |  |  |  |  |  |  |
|  | time (linear) | | .6662 | | *** | .6543 | *** | .6901 | *** |  |  |
|  | time (quadratic) | | -.0442 | | *** | -.0433 | *** | -.0457 | *** |  |  |
|  | time (cubic) | | .0007 | | *** | .0007 | *** | .0008 | *** |  |  |
| Education | | |  | |  |  |  |  |  |  |  |
|  | LSE | | -.5521 | | *** | -.5441 | *** | -.5290 | *** |  |  |
|  | HSE | | -1.9809 | | *** | -1.9084 | *** | -1.9240 | *** |  |  |
|  | HEST | | -4.4520 | | *** | -4.2735 | *** | -4.2370 | *** |  |  |
|  | HELT | | -7.3924 | | *** | -7.1940 | *** | -7.4023 | *** |  |  |
| In education | | | -.9219 | | *** | -.9508 | *** | -.8944 | *** |  |  |
| Years graduated | | |  | |  |  |  |  |  |  |  |
|  | Linear | | .0893 | | *** | .0859 | *** | .0924 | *** |  |  |
|  | Quadratic | | -.0039 | | *** | -.0038 | *** | -.0041 | *** |  |  |
| Education*Baseline | | |  | |  |  |  |  |  |  |  |
|  | LSE*time (linear) | | .0668 | | *** | .0637 | *** | .0618 | *** |  |  |
|  | LSE*time (quadratic) | | -.0021 | | *** | -.0019 | *** | -.0019 | *** |  |  |
|  | HSE*time (linear) | | .2295 | | *** | .2173 | *** | .2169 | *** |  |  |
|  | HSE*time (quadratic) | | -.0061 | | *** | -.0056 | *** | -.0055 | *** |  |  |
|  | HEST*time (linear) | | .5216 | | *** | .4963 | *** | .4875 | *** |  |  |
|  | HEST*time (quadratic) | | -.0135 | | *** | -.0126 | *** | -.0122 | *** |  |  |
|  | HELT*time (linear) | | .7887 | | *** | .7597 | *** | .7786 | *** |  |  |
|  | HELT*time (quadratic) | | -.0188 | | *** | -.0177 | *** | -.0181 | *** |  |  |
| Age*Gross Domestic Product (GDP, Lag 1) | | | | | | |  |  |  |  |  |
|  | Age1519*GDP Lag1 | | .0001 | | *** |  |  |  |  |  |  |
|  | Age2024*GDP Lag1 | | .0001 | | *** |  |  |  |  |  |  |
|  | Age2529*GDP Lag1 | | .0000 | |  |  |  |  |  |  |  |
|  | Age3034*GDP Lag1 | | -.0000 | | *** |  |  |  |  |  |  |
|  | Age3539*GDP Lag1 | | .0002 | | *** |  |  |  |  |  |  |
|  | Age4044*GDP Lag1 | | .0001 | | *** |  |  |  |  |  |  |
|  | Age4549*GDP Lag1 | | -.0012 | | *** |  |  |  |  |  |  |
| Education*Gross Domestic Product (GDP, Lag 1) | | | | | | |  |  |  |  |  |
|  | LE*GDP Lag1 | |  | |  | .0001 | *** |  |  |  |  |
|  | LSE*GDP Lag1 | |  | |  | .0001 | *** |  |  |  |  |
|  | HSE*GDP Lag1 | |  | |  | .0000 | *** |  |  |  |  |
|  | HEST*GDP Lag1 | |  | |  | -.0000 |  |  |  |  |  |
|  | Helt*GDP Lag1 | |  | |  | .0000 |  |  |  |  |  |
| Education*Age*Gross Domestic Product (GDP, Lag 1) | | | | | | | |  |  |  |  |
|  | LE*Age1519*GDP Lag1 | | |  |  |  |  | .0001 | *** |  |  |
|  | LE*Age2024*GDP Lag1 | | |  |  |  |  | .0001 | *** |  |  |
|  | LE*Age2529*GDP Lag1 | | |  |  |  |  | -.0001 | *** |  |  |
|  | LE*Age3034*GDP Lag1 | | |  |  |  |  | .0002 | *** |  |  |
|  | LE*Age3539*GDP Lag1 | | |  |  |  |  | .0006 | *** |  |  |
|  | LE*Age4044*GDP Lag1 | | |  |  |  |  | .0003 | *** |  |  |
|  | LE*Age4549*GDP Lag1 | | |  |  |  |  | -.0015 | *** |  |  |
|  | LSE*Age1519*GDP Lag1 | | |  |  |  |  | .0001 | *** |  |  |
|  | LSE*Age2024*GDP Lag1 | | |  |  |  |  | .0001 | *** |  |  |
|  | LSE*Age2529*GDP Lag1 | | |  |  |  |  | -.0000 | *** |  |  |
|  | LSE*Age3034*GDP Lag1 | | |  |  |  |  | .0001 | *** |  |  |
|  | LSE*Age3539*GDP Lag1 | | |  |  |  |  | .0005 | *** |  |  |
|  | LSE*Age4044*GDP Lag1 | | |  |  |  |  | .0002 | *** |  |  |
|  | LSE*Age4549*GDP Lag1 | | |  |  |  |  | -.0013 | *** |  |  |
|  | HSE*Age1519*GDP Lag1 | | |  |  |  |  | .0000 | ** |  |  |
|  | HSE*Age2024*GDP Lag1 | | |  |  |  |  | .0001 | *** |  |  |
|  | HSE*Age2529*GDP Lag1 | | |  |  |  |  | -.0000 | ** |  |  |
|  | HSE*Age3034*GDP Lag1 | | |  |  |  |  | .0000 | * |  |  |
|  | HSE*Age3539*GDP Lag1 | | |  |  |  |  | .0002 | *** |  |  |
|  |  | | |  |  |  |  |  |  |  |  |
|  | *continued on next page* | | |  |  |  |  |  |  |  |  |

*Table A.3 (con’t) Models 0-12 using gross domestic product (GDP) as macroeconomic indicator*

|  | |  | **Model 5** | | | **Model 6** | | **Model 7** | |  | |
| --- | --- | --- | --- | --- | --- | --- | --- | --- | --- | --- | --- |
|  | |  | **b** | | **sig.** | **b** | **sig.** | **b** | **sig.** |  |  |
|  | | |  | |  |  |  |  |  |  |  |
|  | HSE*Age4044* GDP Lag1 | | |  |  |  |  | -.0000 |  |  |  |
|  | HSE*Age4549* GDP Lag1 | | |  |  |  |  | -.0011 | *** |  |  |
|  | HEST*Age1519* GDP Lag1 | | |  |  |  |  | -.0000 |  |  |  |
|  | HEST*Age2024* GDP Lag1 | | |  |  |  |  | -.0000 | *** |  |  |
|  | HEST*Age2529* GDP Lag1 | | |  |  |  |  | .0000 | *** |  |  |
|  | HEST*Age3034* GDP Lag1 | | |  |  |  |  | -.0001 | *** |  |  |
|  | HEST*Age3539* GDP Lag1 | | |  |  |  |  | .0000 | ** |  |  |
|  | HEST*Age4044* GDP Lag1 | | |  |  |  |  | .0000 |  |  |  |
|  | HEST*Age4549* GDP Lag1 | | |  |  |  |  | -.0009 | *** |  |  |
|  | HELT*Age1519* GDP Lag1 | | |  |  |  |  | .0010 | *** |  |  |
|  | HELT*Age2024* GDP Lag1 | | |  |  |  |  | -.0000 |  |  |  |
|  | HELT*Age2529* GDP Lag1 | | |  |  |  |  | .0001 | *** |  |  |
|  | HELT*Age3034* GDP Lag1 | | |  |  |  |  | -.0001 | *** |  |  |
|  | HELT*Age3539* GDP Lag1 | | |  |  |  |  | -.0000 | ** |  |  |
|  | HELT*Age4044* GDP Lag1 | | |  |  |  |  | .0000 |  |  |  |
|  | HELT*Age4549* GDP Lag1 | | |  |  |  |  | -.0007 | *** |  |  |
| Constant | | | -5.1267 | | *** | -5.0608 | *** | -5.2337 | *** |  |  |
|  | | |  | |  |  |  |  |  |  |  |
| N | | | 46,137,850 | | | 46,137,850 | | 46,137,850 | |  | |
| Log Likelihood | | | -8004017.1 | | | -8005762.7 | | -8001978.3 | |  | |
